# An updated Bioconductor workflow for correlation profiling subcellular proteomics

**DOI:** 10.12688/f1000research.165543.1

**Published:** 2025-07-21

**Authors:** Charlotte Hutchings, Thomas Krueger, Oliver M Crook, Laurent Gatto, Kathryn S Lilley, Lisa M Breckels

**Affiliations:** 1 Cambridge Centre for Proteomics, Department of Biochemistry, University of Cambridge, Cambridge, CB2 1QR, UK; 2 Department of Biochemistry, University of Cambridge, Cambridge, CB2 1QR, UK; 3 Department of Chemistry, University of Oxford, Oxford, OX1 3QU, UK; 4 Computational Biology and Bioinformatics (CBIO), de Duve Institute - UCLouvain, Avenue Hippocrate, 74 - B1.74.10, 1200 Brussels, Belgium

**Keywords:** Subcellular spatial proteomics, correlation profiling, mass spectrometry, protein localisation, LOPIT, QFeatures, pRoloc, bandle

## Abstract

**Background:**

Subcellular localisation is a determining factor of protein function. Mass spectrometry-based correlation profiling experiments facilitate the classification of protein subcellular localisation on a proteome-wide scale. In turn, static localisations can be compared across conditions to identify differential protein localisation events.

**Methods:**

Here, we provide a workflow for the processing and analysis of subcellular proteomics data derived from mass spectrometry-based correlation profiling experiments. This workflow utilises open-source R software packages from the Bioconductor project and provides extensive discussion of the key processing steps required to achieve high confidence protein localisation classifications and differential localisation predictions. The workflow is applicable to any correlation profiling data and supplementary code is provided to help users adapt the workflow to DDA and DIA data processed with different database softwares.

**Results:**

The workflow is divided into three sections. First we outline data processing using the QFeatures infrastructure to generate high quality protein correlation profiles. Next, protein subcellular localisation classification is carried out using machine learning. Finally, prediction of differential localisation events is covered for dynamic correlation profiling experiments.

**Conclusions:**

A comprehensive start-to-end workflow for correlation profiling subcellular proteomics experiments is presented.

**R version**: R version 4.5.0 (2025-04-11)

**Bioconductor version**: 3.21

## Introduction

**Figure 1.**
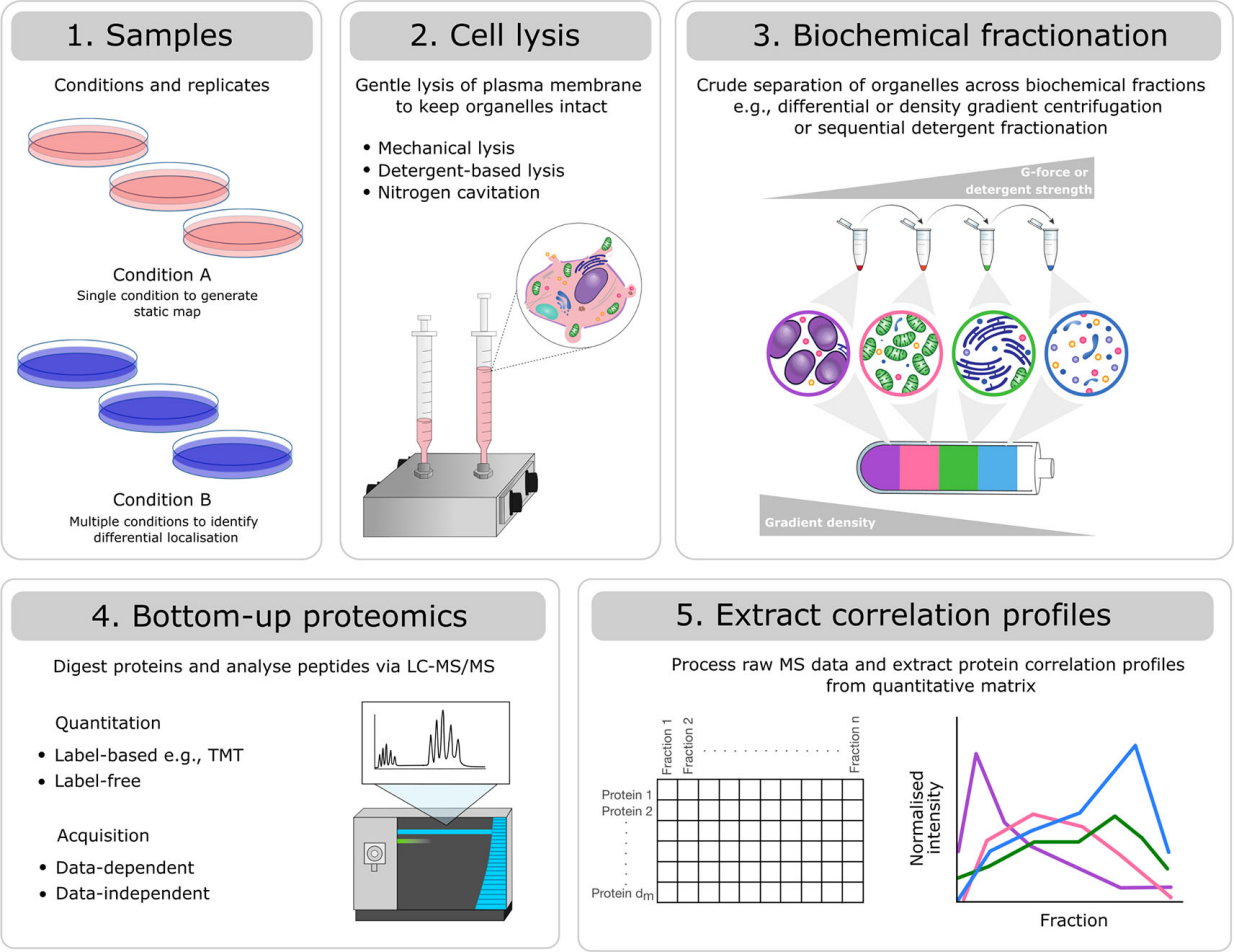
Modular experimental design of a mass spectrometry-based correlation profiling experiment. Experiments will differ with respect to: 1) the number of samples (replicates and conditions), 2) the method for gentle cell lysis, 3) the biochemical fractionation approach selected to generate crude separation of organelles, 4) the peptide quantitation method (label-based vs. label-free) and mass spectrometry acquisition method (data-dependent vs. data-independent), and 5) the processing software used to produce a quantitative matrix of pepide spectrum matches (PSMs), peptides or proteins across biochemical fractions. Despite these differences, all types of correlation profiling-based subcellular proteomics experiments can be analysed using the presented workflow.

The field of subcellular spatial proteomics is centered on the determination of protein localisation within the cell. Importantly, protein localisation is intimately linked to protein function and is vital for research into basic biological mechanisms impacting the health and disease of all organisms. Quantitative mass spectrometry (MS)-based proteomics methods have been developed to study protein localisation at the proteome-wide level and generate subcellular “maps”. Several high-throughput MS-based correlation profiling methods have been established and include the LOPIT family of methods, ^1– 3^ dynamic organellar maps (DOMs), ^4^ protein correlation profiling (PCP), ^5^ sequential detergent-based solubilisation, ^6^ COLA, ^7^ Prolocate ^8^ and SubCellBarCode. ^9^ A summary of the overall experimental steps taken in a correlation profiling experiment and how these may differ between methods is provided in Figure 1. Generation of protein correlation profiling data requires considerable effort, expense and well thought-out experimental designs for successful implementation. Equally, data analysis involves considerable time and investment to produce high quality, robust datasets which enable meaningful biological interpretation and utility to the research community.

Here, we provide an updated workflow for the processing and analysis of correlation profiling-based subcellular spatial proteomics data. We cover the key steps required to extract spatial information from quantitative correlation profiling MS data. The first step requires data importation, processing, and quality control followed by the definition of subcellular markers. We discuss how to curate such markers using approaches including unsupervised clustering and dimensionality reduction. Next, we show how to use subcellular markers to generate subcellular spatial proteome maps. This involves the classification of protein localisation through classical or Bayesian semi-supervised machine learning algorithms using the pRoloc package. Finally, we demonstrate the use of Bayesian Analysis of Differential Localisation Experiments (BANDLE) ^10^ through the bandle package to discover proteins which are differentially localised in the cell under different conditions. This workflow differs from our previous two workflows for spatial proteomics ^11, 12^ with respect to (i) the infrastructure used for data storage and processing and (ii) the analysis of dynamic spatial proteomics experiments in which the goal is to discover differentially localised proteins. We also provide a complete end-to-end workflow following a real-life use case.

In this workflow we use R packages from Bioconductor, namely, the QFeatures package for upstream data processing and the pRoloc and bandle packages for downstream machine learning. In our first F1000 workflow for spatial proteomics ^11^ we used the MSnbase package and the MSnSet infrastructure for storing and manipulating our spatial proteomics data. In this workflow, we have transitioned to working with the QFeatures package for data processing. Whilst MSnbase provides functionality for manipulating and storing quantitative proteomics data, the QFeatures infrastructure makes it possible to store all levels of the data together in one object with explicit links maintained between hierarchical levels. For example, it is possible to store the peptide spectrum matches (PSMs), peptides and proteins (among other types of data) derived from all replicates of an experiment together, in one single data container.

As a use case, we analyse a dynamic LOPIT-DC proteomics dataset. ^13^ We first demonstrate how to capture the localisation of thousands of proteins in A549 cells and generate a spatial map from this information. We then extend the workflow to identify proteins which are differentially localised upon perturbation of the cells, specifically a 12-hour exposure to 6 Gy x-ray radiation to induce DNA damage. Experiments were conducted in triplicate and the samples were analysed and collected when cells were (1) unstimulated and (2) at 12 hours following stimulation with x-ray radiation. Although the use case data is derived from a LOPIT-DC experiment, the steps of this workflow can be applied to any subcellular proteomics experiment which generates data in the form of protein correlation profiles across multiple fractions.

### Package installation

The installation of Bioconductor packages is well-documented, please see the Bioconductor Installation page for more details. The main packages we will use in this workflow are QFeatures, pRoloc and bandle. The below code chunk shows how to install these R Bioconductor packages.

if (!require("BiocManager", quietly = TRUE))
    install.packages("BiocManager")

BiocManager::install(c("QFeatures",
                       "pRoloc",
                       "bandle"))



We also make use of several other R packages, to install them we can again use BiocManager::install.

BiocManager::install(c("tidyverse",
                       "dbscan",
                       "clusterProfiler",
                       "org.Hs.eg.db",
                       "pRolocGUI",
                       "pRolocdata",
                       "colorspace",
                       "ggpubr",
                       "gridExtra",
                       "ggpubr",
                       "pheatmap"))



This procedure is applicable to any packages, from CRAN and Bioconductor, as well as GitHub. Once a package has been installed, it needs to be loaded for its functionality to become available in the R session. This is done with the library function e.g., to load the QFeatures package one would type library("QFeatures") after installation.

library("QFeatures")
library("pRoloc")
library("bandle")
library("tidyverse")
library("dbscan")
library("clusterProfiler")
library("org.Hs.eg.db")
library("pRolocGUI")
library("pRolocdata")
library("gridExtra")
library("ggpubr")
library("pheatmap")



If you have questions about this workflow, or about other Bioconductor packages in general, they are best asked on the Bioconductor support site following the posting guidelines. Questions can be tagged with specific package names or keywords.

### Navigating this workflow

Here, we present a start-to-finish workflow for the analysis of subcellular spatial proteomics data derived from an MS-based correlation profiling experiment. Depending on the experimental goal (e.g., generation of static maps versus discovery of differentially localised proteins) and level of data available (e.g., PSM, peptide or protein), users may only need to follow part of the workflow. For simplicity, we have divided the workflow into three parts:

1. Data processing and generation of protein correlation profiles using QFeatures
2. Protein localisation prediction for static maps using pRoloc
3. Differential localisation prediction for dynamic experiments using bandle

**Figure 2.**
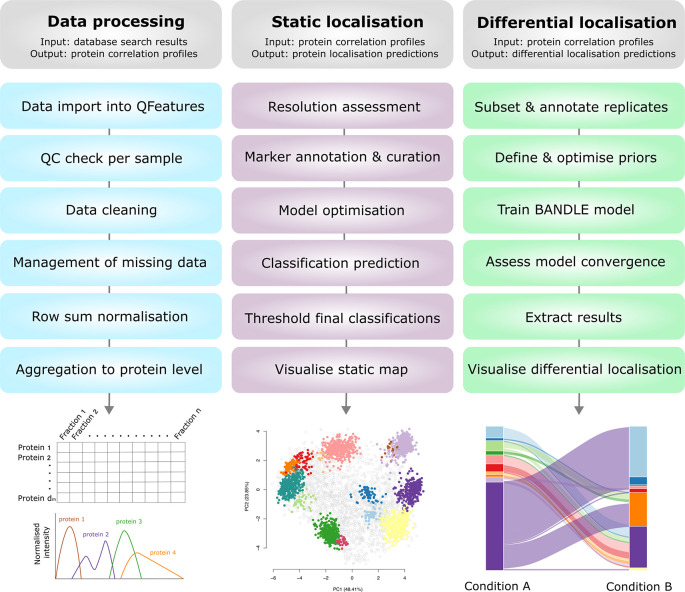
A schematic summary of the presented workflow. The input and output of each part of the workflow are outlined and visualised. Users may only need to complete a portion of this workflow depending upon their initial data input and experimental question(s).

The main steps taken in each part of the workflow are outlined in Figure 2.

## Part 1: Data processing and quality control within the QFeatures infrastructure

The first part of this workflow will demonstrate how to import quantitative MS data into R using the QFeatures infrastructure. We will then discuss the key data cleaning and quality control steps required to ensure that only high-quality data is retained. Finally, the quantitative data will be transformed into protein correlation profiles which hold the spatial information required for protein localisation prediction, as covered in part 2 of the workflow.

### Use case: Dynamic spatial proteomics of A549 human adenocarcinoma cells

Using the LOPIT-DC proteomics platform ^2^ we obtained spatial maps that capture the subcellular localisation of thousands of proteins in the A549 human lung adenocarcinoma cell line under two separate conditions. ^13^ The aim of this experiment was to elucidate changes in protein subcellular localisation during x-ray radiation to determine potential radioresistance mechanisms.

Experiments were conducted in triplicate and the samples were collected and analysed under two conditions; when cells were (1) unstimulated (unstim) and (2) at 12 hours following stimulation with 6 Gy x-ray radiation (xray). Replicates are denoted 1, 2 and 3. The LOPIT-DC method ^2^ combines biochemical cellular fractionation with multiplexed high-resolution mass spectrometry (MS). The full protocol and experimental design can be found in Christopher et al. ^13^ Briefly, TMT labelling was conducted as described in Christopher et al. and eight biochemical fractions were labelled. ^13^ Each replicate consisted of quantitative data derived from a single TMTpro ^TM^ 16plex with eight labels for the unstimulated and xray conditions, respectively. This resulted in a single raw dataset per replicate, and three across the complete dynamic LOPIT experiment. For clarity, within this workflow the term “sample” refers to one LOPIT-DC gradient carried out on a single sample of cells and the term “experiment” refers to two LOPIT-DC gradients - one carried out on unstimulated cells and one on xray stimulated cells. Hence, there were three replicate experiments, each comprising two samples, giving a total of six samples.

In any MS-based proteomics experiment the first step involves running an identification search using the raw mass spectrometry data. Most commonly, searches are performed using third party software. Here, the raw data was processed using the Proteome Discoverer software version 3.1 with the SequestHT search engine. Such software has powerful algorithms that can match raw MS spectra to peptides by comparing the experimental spectra to a large database of theoretical spectra generated via an *in-silico* digestion of the proteome. All TMT-labelled samples were analysed using the same processing and consensus workflows, as provided at Zenodo under the doi: 10.5281/zenodo.15100485. The MS data was searched against a Swiss-Prot *Homo sapiens* database (no isoforms, downloaded 20/01/2024) and the Protein Contaminant Libraries for DIA and DDA Proteomics. ^14^ Once all replicates had been collected, all samples within the experiment were analysed using a multi-consensus workflow (i.e., individual processing workflows were subsequently analysed in one consensus workflow) to ensure consistent protein grouping. This gives the output of all samples as a single .txt file. We export the data at the PSM level and perform data filtering and aggregation to proteins in R using QFeatures. This allows for maximum understanding and flexibility of data processing.

Of note, we have selected this use-case as it represents a real-life dataset with multiple conditions and replicates. Consequently, Part 1 of the workflow presented here appears more complex than our previous workflows ^11, 12^ which do not go into detail regarding the processing of multiple samples. Nevertheless, we hope that users will appreciate the discussion and demonstration of how to deal with complete multi-replicate, multi-condition experiments.

### Adapting the workflow to other protein correlation profiling methods

As MS-based protein correlation profiling has grown in popularity the methods used to obtain such profiles have diversified. In particular, methods differ with respect to cell lysis, biochemical fractionation approach (e.g., differential centrifugation, density gradient centrifugation, detergent-based methods), quantitation methods (label-based vs. label-free) and MS acquisition method (data-dependent vs -independent acquisition). ^15^ As a result of these experimental differences, there are variations in the structure of data generated.

As well as differences in experimental methodology, various third-party software can be used to carry out a database search of the raw MS files. These include but are not limited to Proteome Discoverer, MaxQuant, FragPipe and DIA-NN. Since the use-case data was processed using Proteome Discoverer v3.1, part 1 of this workflow will use specific columns and files output by this software. Nevertheless, other third-party software will output similar files containing comparable information, thus making this workflow easy to adapt. An appendix is available with supplementary information on how to adapt this workflow for other use-cases and is deposited on the GitHub repository https://github.com/CambridgeCentreForProteomics/f1000_subcellular_proteomics and Zenodo with the doi: 10.5281/zenodo.15100485. An example is provided on how to modify Part 1 of this workflow for data processed by MaxQuant. We also discuss key considerations for DIA protein correlation profiling in the appendix and provide a sample workflow for DIA data processed using DIA-NN.

### Downloading the data

The files used in this workflow can all be found deposited to Zenodo with the doi: 10.5281/zenodo.15100485 and at the associated GitHub repository. We recommend that users first set their working directory in R using the setwd function or by navigating to Session -> Set Working Directory menu if using RStudio. Users should then download the necessary files into their working directory to follow the workflow. Alternatively, RStudio users could benefit from generating an RStudio project. The advantage of using an RStudio project is that the project itself acts as a base working directory and all files that are read in or written out can be done so using paths relative to the project. For more details on the use of RStudio projects users are referred to “Using RStudio Projects”. The raw MS files for the use-case data have been deposited to the ProteomeXchange Consortium via PRIDE ^16, 17^ under the identifier PXD055123. Of note, raw files were re-processed with an updated Swiss-Prot *Homo sapiens* database and newer version of Proteome Discoverer (v3.1) to generate the use-case data for this workflow. As a result, the data used here differ slightly from those used by Christopher et al. ^13^

### Importing the data into R and creating a QFeatures object

In this workflow we make use of the R Bioconductor QFeatures package for storing and processing quantitative high-throughput MS data. ^18^ The QFeatures package provides an elegant infrastructure to store and manage quantitative MS data. Data is loaded into R and imported into a QFeatures object which is based on the Bioconductor SummarizedExperiment and MultiAssayExperiment classes.

### Structure of a QFeatures object

**Figure 3.**
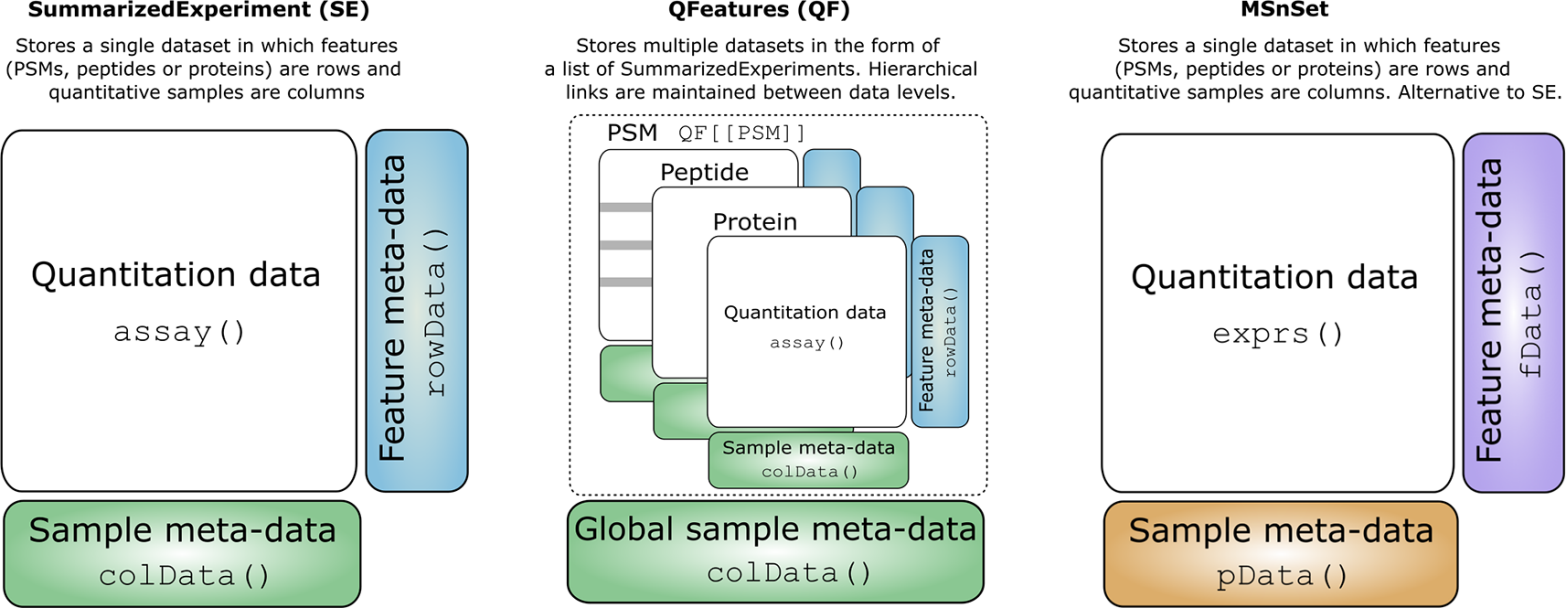
Conceptual representation of SummarizedExperiment, QFeatures and MSnSet objects. Each data structure has several data slots which can be accessed with the corresponding functions.

Data in a QFeatures object often have hierarchical relation. For example, proteins are composed of peptides, and peptides are composed of PSMs. In QFeatures users can store these data levels together in one dedicated object and the relation between the data levels is preserved. This makes it straightforward for users to navigate across all levels of the data, for example, tracking peptides and proteins of interest in all data levels. Specifically, under the QFeatures infrastructure each quantitative dataset (referred to as an *experimental set*) is stored within a SummarizedExperiment object. ^19^ SummarizedExperiment objects are matrix-like containers where the rows represent features of interest derived from the identification search and the columns represent quantitative channels. In our case, the rows represent information about PSMs, peptides or proteins and columns represent the quantitation of biochemical fractions of a correlation profiling experiment. Within each SummarizedExperiment there are 3 main slots: (1) the assay slot for storing the quantitation data (2) the colData slot for sample (quantitative column) meta data (3) the rowData to store the feature data (rows) derived from the identification search. The structure of QFeatures and SummarizedExperiment objects are shown in Figure 3.

There will be many users who are familiar with MSnbase and later in this workflow we will make use of the MSnSet class. Like QFeatures objects, MSnSets are also data containers for MS data and use similar accessors and functions. For convenience, Table 1 shows the equivalent functions between the two packages to help MSnbase and QFeatures users work between the two data structures. This comparison is also illustrated in Figure 3.

**Table 1.** Equivalent accessors/slot names between MSnbase and QFeatures.

Description	MSnbase function	QFeatures function
Extract quantitative data	exprs	assay
Extract feature data	fData	rowData
Extract experiment data	pData	colData
The quantitative column names	sampleNames	colnames
Column names of feature data	fvarLabels	rowDataNames
Row names of the dataset	featureNames	rownames

### Initial import of the data

We begin our data analysis by importing the PSM-level .txt file which we have generated from Proteome Discoverer (PD). Generally, it is possible to output the data at any quantitation level e.g. PSMs, peptides or proteins, using PD, MaxQuant, or other common third-party MS software. We prefer and advise users to output the data at the lowest possible level so that they have full control over how their data is filtered and aggregated. For TMT data this corresponds to the PSM level. For users analysing label-free quantitative (LFQ) data the lowest data level output is the PSM, precursor or peptide level, depending on which third party software was used. We discuss the differences between TMT and LFQ data analysis in more detail in our sister workflow. ^20^

We begin by importing the data as a data.frame. We use the read.delim function to read the PSM-level .txt file into R.

## Tell R the file location
f <- "a549_uv_lopit_PSMs.txt"

## Import into a dataframe
df <- read.delim(f)



### Preparing the data for conversion to QFeatures

Now we need to do some housekeeping in order to transfer our data from a data.frame into a QFeatures object. There are a number of ways in which to create a QFeatures object, and this is described in detail in the QFeatures vignettes. Two main scenarios for data import are proposed; the single-set case and multi-set case. The difference here is that single-set data import will generate a QFeatures object with all data from the given data.frame stored in a single SummarizedExperiment (or experimental set) whilst multi-set data import allows users to generate a single QFeatures object with the data split into multiple experimental sets. For example, users can split their data by replicate, condition or sample during the import such that these data can be processed and analysed separately. The most appropriate method for data import into a QFeatures object will depend on the experimental design and data structure. For users with DDA data (in a wide format), the three main scenarios are outlined below. Users with DIA data (in a long format) are referred to the appendix for details on data import into QFeatures.

1. *Users with a single sample (LFQ or TMT): No need to split data*
Users with a single correlation profiling sample (i.e., one set of biochemical fractions from one cell sample) are advised to use single-set import. As mentioned above, single-set import will generate a QFeatures object with one experimental set containing all data. This experimental set will contain features (PSMs, peptides or proteins) down the rows and quantitative channels (biochemical fractions) across the columns. Single-set import is described in the QFeatures vignette and demonstrated in Hutchings et al. ^20^ Briefly, users should 1) load their data into a data.frame, 2) identify the columns containing quantitative data (here biochemical fractions), and 3) pass the data.frame object to the assayData argument of readQFeatures and the indices of quantitative columns to the quantCols argument. Provide a name to the experimental set using the name argument. The colData can be added during import or annotated after import.
2. *Users with multiple samples analysed by LFQ: Split data by columns*
Users with multiple correlation profiling samples (i.e., multiple replicates or conditions) analysed by LFQ will likely have the results of an identification search output with one row per feature (PSM, peptide or protein) and one quantitative column per MS run (biochemical fraction). In this case, users will need to split the data by columns to get one dataset per sample. An example of how to import this type of data file into a QFeatures object and split the columns into individual sets is provided in the appendix which has been deposited on the GitHub repository and Zenodo with the doi: 10.5281/zenodo.15100485.
3. *Users with multiple samples analysed by label-based multiplexing (e.g., TMT): Split data by rows*
Users with multiple correlation profiling samples (i.e., multiple replicates or conditions) analysed across several multiplexed MS runs (e.g., several TMTplexes) will also need to split the data into multiple sets to analyse samples separately. When processing such data using third party software such as PD, the results across multiple MS runs are concatenated into a single data table. For example, after carrying out an identification search on data derived from multiple TMTplexes, the data are concatenated such that there is one quantitative column per TMT label, but not one per TMT label per TMTplex. This means that each quantitative column contains data for more than one sample. In this case, the user will need to split the data by rows to get one dataset per sample. This is the case for the use-case data and is discussed and exemplified below.

As already discussed above, our use-case data are triplicate experiments from three independent multiplexed TMT MS analyses, and the output PSM-level .txt file from the PD search is one single tabular file. Therefore, we follow the guidelines for data import for the multi-set case (C) such that we generate a single QFeatures object with each experimental replicate stored in a separate experimental set. This will allow us to look at and process the data on a per-replicate basis.

To use the readQFeatures function to convert our single data.frame object containing all replicates of all conditions into a single QFeatures object with one experimental set per replicate, we first must prepare the data. Specifically, for multi-set import, we need to (1) locate the columns in the data.frame that contain the quantitation data, (2) create a column in the data.frame that contains information on which replicate the data was derived from (this is the column we will use to split the rows into multiple experimental sets), and lastly (3) create a DataFrame object combining this information and describing the experimental design.

1. **Locating the quantitation information.** By examining the column names of the data.frame object, df, we can readily see that the quantitation data is found in columns 46 through to 61. These 16 columns represent the 16 labels from our TMTpro ^TM^ 16plexes. As we can see by the fact that we only have 16 columns, the quantitation across our three TMTplexes is currently combined in the output from PD. This is why we want to split the data using a multi-set import.

## Check column names of imported file
df %>%
  names()


##  [1] "Checked"                           "Tags"
##  [3] "Confidence"                        "Identifying.Node.Type"
##  [5] "Identifying.Node"                  "Search.ID"
##  [7] "Identifying.Node.No"               "PSM.Ambiguity"
##  [9] "Sequence"                          "Annotated.Sequence"
## [11] "Modifications"                     "Contaminant"
## [13] "Number.of.Proteins"                "Master.Protein.Accessions"
## [15] "Master.Protein.Descriptions"       "Protein.Accessions"
## [17] "Protein.Descriptions"              "Number.of.Missed.Cleavages"
## [19] "Charge"                            "Original.Precursor.Charge"
## [21] "Delta.Score"                       "Delta.Cn"
## [23] "Rank"                              "Search.Engine.Rank"
## [25] "Concatenated.Rank"                 "mz.in.Da"
## [27] "MHplus.in.Da"                      "Theo.MHplus.in.Da"
## [29] "Delta.M.in.ppm"                    "Delta.mz.in.Da"
## [31] "Ions.Matched"                      "Matched.Ions"
## [33] "Total.Ions"                        "Intensity"
## [35] "Activation.Type"                   "MS.Order"
## [37] "Isolation.Interference.in.Percent" "SPS.Mass.Matches.in.Percent"
## [39] "Average.Reporter.SN"               "Ion.Inject.Time.in.ms"
## [41] "RT.in.min"                         "First.Scan"
## [43] "Last.Scan"                         "Master.Scans"
## [45] "File.ID"                           "Abundance.126"
## [47] "Abundance.127N"                    "Abundance.127C"
## [49] "Abundance.128N"                    "Abundance.128C"
## [51] "Abundance.129N"                    "Abundance.129C"
## [53] "Abundance.130N"                    "Abundance.130C"
## [55] "Abundance.131N"                    "Abundance.131C"
## [57] "Abundance.132N"                    "Abundance.132C"
## [59] "Abundance.133N"                    "Abundance.133C"
## [61] "Abundance.134N"                    "Quan.Info"
## [63] "Peptides.Matched"                  "XCorr"
## [65] "Number.of.Protein.Groups"          "Spectral.Angle"
## [67] "q.Value"                           "PEP"
## [69] "SVM.Score"



We can also use the grep function to extract this information since the quantitative columns in our PD file contain the prefix “Abundance”. We save this information in a numerical vector called quantID.

## Create vector with indices of quantitative columns
quantID <- grep("^Abundance", colnames(df))



If desired at this stage, we can also simplify the column names of the data. For ease of plotting downstream we remove the prefix “Abundance.” from the quantitation columns.

## Current names
colnames(df[quantID])


##  [1] "Abundance.126"  "Abundance.127N" "Abundance.127C" "Abundance.128N"
##  [5] "Abundance.128C" "Abundance.129N" "Abundance.129C" "Abundance.130N"
##  [9] "Abundance.130C" "Abundance.131N" "Abundance.131C" "Abundance.132N"
## [13] "Abundance.132C" "Abundance.133N" "Abundance.133C" "Abundance.134N"


## Change column names
colnames(df)[quantID] <- gsub("Abundance.", "", colnames(df[quantID]))

## Verify
colnames(df[quantID])


##  [1] "126"  "127N" "127C" "128N" "128C" "129N" "129C" "130N" "130C" "131N"
## [11] "131C" "132N" "132C" "133N" "133C" "134N"



2. **Defining factor information to split the data by (replicate or sample)**. The data we have imported contains samples of all replicates and conditions together in one file. Data is commonly output in this format from third party proteomics software to ensure that protein grouping is consistent across experiments. Of note, in our use-case experiment the two conditions of each replicate were analysed in a single TMTpro ^TM^ 16plex with eight labels for the unstimulated and x-ray conditions, respectively. The quantitation for all 16 TMT reporter ions (representing the 16 labelled biochemical fractions) is derived from a single PSM. Consequently, it is necessary to carry out quality control on a per TMTplex/replicate basis rather than individual samples. In theory, we could split the data into individual samples, but the QC for both samples of a given replicate would be exactly the same (as they are derived from the same PSMs). Therefore, we will here import the three replicates as separate experimental sets within a QFeatures object. For users who have experimental designs that include each sample in a separate TMTplex (e.g., six samples labelled across six TMTplexes) or are label-free, it may be useful to adapt this code to define the sample information and generate a separate experimental set for each sample rather than just each replicate.

Each PSM in the dataset is already annotated in the File.ID column to indicate which raw MS file it was derived from. Each pooled TMTplex underwent offline pre-fractionation via HPLC to generate 18 fractions for MS analysis, with one raw file removed from the analysis of replicate 1 due to low quality. Hence, the data included 53 MS runs and raw files; six samples comprising three replicates across three TMTpro ^TM^ 16plexes, each with 17 or 18 fractions.

Let’s examine the File.ID column in df.

## Check how many raw file IDs there are
df %>%
  pull(File.ID) %>%
  unique() %>%
  length()


## [1] 53


## Take a look at the first 6 raw file IDs
df %>%
  pull(File.ID) %>%
  unique() %>%
  head()


## [1] "F1.1" "F1.2" "F1.3" "F1.4" "F1.5" "F1.6"



As expected, we see that there are 53 raw files in this experiment. The raw file name in File.ID is an identifier given to each raw file during the Proteome Discoverer identification search rather than the raw file name itself. Other software may store this information differently, using a different column identifier, for example. We urge users to familarise themselves with the third-party software that they have used for the identification search and check the output settings.

Table 2 outlines how the File.IDs correspond to the raw MS files used in the experiment. The value of ‘x’ represents the HPLC fraction number 1-17 or 18, as discussed above.

**Table 2.** Raw Spectrum File naming scheme for use-case LOPIT data.

File Name	File.ID	Replicate
TMT_Pro_1.x	F1.x	rep1
TMT_Pro_2.x	F2.x	rep2
TMT_Pro_3.x	F3.x	rep3

To make the separation of the dataset by replicate easier and explicit, we will add a column called Replicate to the data.frame to indicate the replicate from which the PSM was derived. We do this based on the value found in the File.ID column, since this tells us the raw file ID and we know which files corresponded to which replicate. If samples from each condition were analysed across separate TMTplexes or using a label-free experimental design, the File.ID column could also be used to create a Sample column for users to separate the data based on both condition and replicate, as discussed above. Here, we use the File.ID column to create a Replicate column indicating which experimental replicate or TMTplex the PSM was derived from.

## Use the sub command to first substitute "F" with "rep"
## Use the sub command to second substitute everything but the rep in File.ID
df$Replicate <- sub("F", "rep", sub("\\.[0-9]+$", "", df$File.ID))

## Verify
df %>%
  pull(Replicate) %>%
  table()


## .
##   rep1   rep2   rep3
## 115302 135169 120343



3. **Creating a** DataFrame **of experimental design information**. So far, we have identified the quantitative columns in our raw data file, edited the names of these columns for convenience of downstream plotting, and added a column to indicate which replicate (i.e., TMTplex) the PSM belongs to. Now we need to summarise this information into a DataFrame that can be used by the readQFeatures function.

Following the documentation from QFeatures, we create a DataFrame object containing columns called runCol and quantCols to allow the function to identify our quantitative data per replicate. The runCol column should contain the identifiers by which we wish to split the data, here the replicate. The quantCols column should contain the names of the quantitative columns. In the QFeatures object we will generate there will be a total of 48 quantitative columns as we have 16 TMT channels multiplied by three replicates. This means that our DataFrame should have 48 rows, one per quantitative column per replicate. We also create a third column called condition to indicate which quantitative columns correspond to which condition (unstimulated or x-ray treated) in each replicate/TMTplex. In QFeatures this data is called the colData ( Figure 3).

## Extract replicate identifiers
rep_ids <- unique(df$Replicate)

## Extract quantitative column names
quant_cols <- colnames(df)[quantID]

## Define conditions
conditions <- c("unstim", "xray")

## Create global colData
samplesInfo <- DataFrame(
  runCol = rep(rep_ids, each = 16),       # repeat rep per quant channel (16)
  quantCols = rep(quant_cols, times = 3), # repeat quant channel per rep (3)
  condition = rep(conditions, each = 8)
)

## Verify
samplesInfo


## DataFrame with 48 rows and 3 columns
##          runCol   quantCols   condition
##     <character> <character> <character>
## 1          rep1         126      unstim
## 2          rep1        127N      unstim
## 3          rep1        127C      unstim
## 4          rep1        128N      unstim
## 5          rep1        128C      unstim
## …             …           …           …
## 44         rep3        132N        xray
## 45         rep3        132C        xray
## 46         rep3        133N        xray
## 47         rep3        133C        xray
## 48         rep3        134N        xray



### Creating the QFeatures object

Now we have all the information we need to use the readQFeatures function to convert the data.frame of our total PSM data into a QFeatures object with three experimental sets, one per replicate.

We pass our data.frame object to the assayData argument, the indices of the quantitative columns (stored in quantID) to the quantCols argument, and the experimental design (stored in samplesInfo) to the colData argument. Finally, we specify which column in our experimental design DataFrame the data should be split by, here "Replicate".

## Now read QF as a multi-set case a per QFeatures documentation
qf <- readQFeatures(assayData = df,          # data.frame containing the data
                    quantCols = quantID,     # location of quant data
                    colData = samplesInfo,   # experimental design
                    runCol = "Replicate")    # column defining replicates

## Verify
qf


## An instance of class QFeatures containing 3 set(s):
##  [1] rep1: SummarizedExperiment with 115302 rows and 16 columns
##  [2] rep2: SummarizedExperiment with 135169 rows and 16 columns
##  [3] rep3: SummarizedExperiment with 120343 rows and 16 columns



By typing qf into the R console we see we have created a QFeatures object which contains three PSM-level datasets ( SummarizedExperiments called *experimental sets*). The summary printed to the screen tells us that we have 115302, 135169, 120343 PSMs for replicates 1, 2 and 3, respectively. We also see we have 16 quantitation columns (TMT channels) per replicate. By default, the multi-set import has resulted in each experimental set being named based on the unique identifiers stored in the column we passed to runCol. We rename these experimental sets to reflect the stage of the data analysis, here the PSM level. This will make it easier to keep track as we aggregate and create new data levels.

## Re-name the sets to reflect the stage of the analysis
names(qf) <- paste0("psms_raw_", names(qf))

## Verify
qf


## An instance of class QFeatures containing 3 set(s):
##  [1] psms_raw_rep1: SummarizedExperiment with 115302 rows and 16 columns
##  [2] psms_raw_rep2: SummarizedExperiment with 135169 rows and 16 columns
##  [3] psms_raw_rep3: SummarizedExperiment with 120343 rows and 16 columns



We can access the first dataset in the QFeatures object by index or by name.

## Access the first experimental set in the 'qf' QFeatures object
qf[[1]]


## class: SummarizedExperiment
## dim: 115302 16
## metadata(0):
## assays(1): ''
## rownames(115302): 1 2 … 115301 115302
## rowData names(54): Checked Tags … SVM.Score Replicate
## colnames(16): rep1_126 rep1_127N … rep1_133C rep1_134N
## colData names(0):


qf[["psms_raw_rep1"]]


## class: SummarizedExperiment
## dim: 115302 16
## metadata(0):
## assays(1): ''
## rownames(115302): 1 2 … 115301 115302
## rowData names(54): Checked Tags … SVM.Score Replicate
## colnames(16): rep1_126 rep1_127N … rep1_133C rep1_134N
## colData names(0):



To access the quantitation data of the first experiment in the QFeatures object we would execute assay(qf[[1]]) or assay(qf[["psms_raw_rep1"]]). To access the PSM feature data, we would use the rowData function in the same way. Finally, the colData function can be used to access the experimental design information we specified earlier in the workflow.

Please see the Quantative Features for Mass Spectrometry Vignette and SummarizedExperiment Vignette for more details on how to access the slots and manipulate QFeatures and SummarizedExperiments objects.

### Data cleaning and quality control

Now we have imported and annotated our PSM-level data we can progress to some quality control assessment and data cleaning. For readers wishing to complete more thorough initial quality control checks prior to processing, we refer to our sister workflow ^20^ where we discuss in detail how to perform (1) an initial quality control of the raw MS data and (2) for experiments which use label-based strategies, how to check the labelling efficiency.

### Quality control checks

We start by examining the quality of the data. It is important to do this for each experimental replicate since these were processed in separate TMTplexes via independent MS analyses. For demonstration we only show evaluation of the first replicate (TMTplex). In reality, users should generate these QC plots for every TMTplex.

# Begin by extracting the rep1 data as a tibble for plotting
obj <- rowData(qf[[1]]) %>%
  as_tibble()

## Plot quality control graphs for that object
ggplot(obj, aes(x = Ion.Inject.Time.in.ms)) +
  geom_density(fill = "grey50") +
  xlab("Ion injection time [ms]")

ggplot(obj, aes(x = log(Delta.M.in.ppm))) +
  geom_density(fill = "grey50", alpha = 0.8) +
  xlab("Mass deviation [ppm]")

ggplot(obj, aes(x = Isolation.Interference.in.Percent)) +
  geom_density(fill = "grey50", alpha = 0.8) +
  xlab("Isolation interference in %")

ggplot(obj, aes(x = log(Average.Reporter.SN))) +
  geom_density(fill = "grey50", alpha = 0.8) +
  xlab("log10 (Average reporter S/N)")

ggplot(obj, aes(x = SPS.Mass.Matches.in.Percent)) +
  geom_histogram(fill = "grey50", colour = "grey20",
                 binwidth = 10, alpha = 0.8) +
  xlab("SPS Mass Matches in %")

dat <- assay(qf[[1]]) %>% longFormat()
dat$names <- gsub("Abundance", "TMT", dat$colname)

ggplot(dat, aes(x = names, y = log(value))) +
  geom_boxplot(fill = "grey50") +
  theme(axis.text.x = element_text(angle = 45, hjust = 1)) +
  ylab("log10 intensity") +
  xlab("TMT channel")



**Figure 4.**
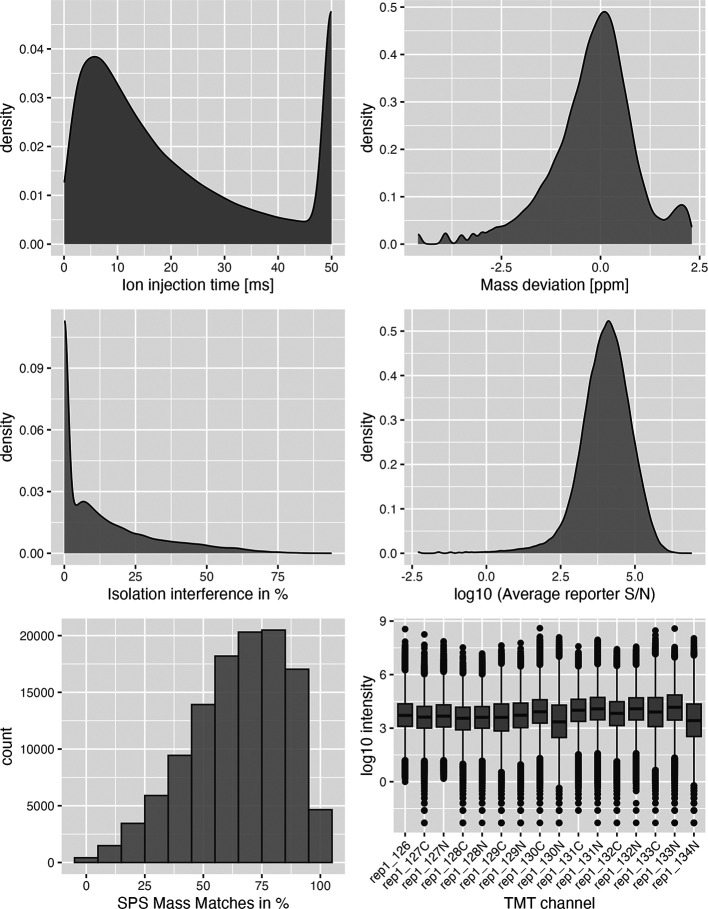
PSM level quality control plots as generated according to Hutchings et al., ^20^ for replicate 1 of the use-case data.

The first two plots ( Figure 4, top left, top right) displaying ion injection time and delta precursor mass can be used to infer whether the mass spectrometry run(s) went as expected. More details on this are provided in Hutchings et al. ^20^ The next three plots ( Figure 4 middle panel, bottom left) show key quality control parameters for TMT data processed using Proteome Discoverer. Importantly, quality control parameters will vary depending on the data type (DDA vs. DIA and TMT vs. LFQ) and processing software used. Users should alter the code to visualise the parameters that are appropriate for their data. Finally, the boxplot of PSM intensity per channel ( Figure 4 bottom right) can be used to verify that approximately the same amount of material from each biochemical fraction was analysed. This is important for the success of the following spatial proteomics workflow as the analysis of unequal peptide quantities from each fraction could skew the protein correlation profiles and lead to a reduction in spatial resolution.

### Making a copy of the raw data

Having checked that the raw data is of sufficiently high quality, we can proceed with the workflow. Before removing any data, we make a copy of our three raw experimental sets. We do this so that we can retain the raw data without overriding it. The getWithColData function can be used to extract an experimental set from a QFeatures object, using i = to specify the name of the set we want. As the name of this function suggests, the colData of this experimental set is automatically extracted too. We name the second copies of our datasets psms_filtered_repX , where X refers to the replicate number. In the subsequent filtering stages will only remove data from these experimental sets.

Users who have completed single-set import and only have one set to copy can use getWithColData to extract a single experimental set and then add this to directly to their QFeatures object using addAssay. A name can be provided directly to the name argument of addAssay, as shown in Hutchings et al. ^20^

## Create copies which we will filter upon
## Use getWithColData to pull the experimental set along with the colData
raw_copy <- list(
  getWithColData(qf, i = "psms_raw_rep1"),
  getWithColData(qf, i = "psms_raw_rep2"),
  getWithColData(qf, i = "psms_raw_rep3")
)

## Specify names for newly generated experimental sets (different from raw)
names(raw_copy) <- paste0("psms_filtered_rep", 1:3)

## Add copy of raw data
qf <- addAssay(x = qf,
               y = raw_copy)

qf


## An instance of class QFeatures containing 6 set(s):
##  [1] psms_raw_rep1: SummarizedExperiment with 115302 rows and 16 columns
##  [2] psms_raw_rep2: SummarizedExperiment with 135169 rows and 16 columns
##  [3] psms_raw_rep3: SummarizedExperiment with 120343 rows and 16 columns
##  [4] psms_filtered_rep1: SummarizedExperiment with 115302 rows and 16 columns
##  [5] psms_filtered_rep2: SummarizedExperiment with 135169 rows and 16 columns
##  [6] psms_filtered_rep3: SummarizedExperiment with 120343 rows and 16 columns



We now have six experimental sets in our QFeatures object. To keep a copy of the raw data we will filter only on the latter three.

### Non-specific data cleaning

The first step towards the removal of unwanted and low quality PSMs is non-specific data cleaning. This includes several cleaning steps that are standard for most quantitative proteomics datasets. Details about these cleaning steps are discussed in our sister workflow. ^20^ Whilst the removal steps themselves are common, users should be aware that the names of parameters may differ in the outputs from different identification search software and may be updated over time. Users should update the presented code accordingly.

Here, we remove:

1. PSMs corresponding to contaminant proteins
2. PSMs which lack a master protein accession
3. PSMs which are not rank 1
4. PSMs which are not unique to a single protein within a single protein group
5. PSMs which are ambiguous

The QFeatures infrastructure provides a convenient function called filterFeatures which removes features (here PSMs) based on conditions derived from the rowData. To use this function we provide a condition for which we only wish to keep features that are evaluated as TRUE. We can also provide the i = argument to limit this filter to specific experimental sets within the QFeatures object, since we only wish to remove data from latter three sets i.e. in this case sets in positions 4, 5 and 6 so we specify i = 4:6. We can also use grep to find the indices of experimental sets containing filtered in their name.

# Define sets to filter upon
sets_to_filter <- grep("filtered", names(qf))

## Basic data cleaning using filterFeatures
qf <- qf %>%
  filterFeatures(~ Contaminant == "False", i = sets_to_filter) %>%
  filterFeatures(~ Master.Protein.Accessions != "", i = sets_to_filter) %>%
  filterFeatures(~ Rank == 1, i = sets_to_filter) %>%
  filterFeatures(~ Search.Engine.Rank == 1, i = sets_to_filter) %>%
  filterFeatures(~ Concatenated.Rank == 1, i = sets_to_filter) %>%
  filterFeatures(~ Number.of.Proteins == 1, i = sets_to_filter) %>%
  filterFeatures(~ Number.of.Protein.Groups == 1, i = sets_to_filter) %>%
  filterFeatures(~ PSM.Ambiguity == "Unambiguous", i = sets_to_filter)



Users who have completed single-set import and only have one experimental set to filter can provide the name of this experimental set directly to the i = argument, as was done in Hutchings et al. ^20^

**An additional note on unique proteins:** As discussed in Hutchings et al., ^20^ the definition of a unique protein will depend on the specific parameters and database(s) used for the identification search. Here, the use-case data was searched against two databases: (1) the SwissProt human proteome without isoforms, and (2) a contaminant database. PSMs corresponding to peptide sequences found in both databases would be filtered out using the code above, which is what we want since we cannot tell if the peptide came from a protein of interest or a contaminant. The same would be true if we had searched against two databases representing different organisms - PSMs matched to sequences in proteins from both organisms would be removed, which would again be favourable. However, there are also cases where filtering for Number.of.Proteins == 1 may not be appropriate. For example, if data are searched against proteomic databases which include isoforms, then it is likely that a much higher proportion of PSMs and peptides would be matched to multiple protein entries (isoforms). As a result, using filterFeatures(~ Number.of.Proteins == 1, i = sets_to_filter) could lead to excessive data loss. Users should consider the implications of filtering for their specific dataset.

### Controlling the false discovery rate

Another key data cleaning step is the control of false discovery rate (FDR). As discussed in Hutchings et al., ^20^ it is necessary to control the FDR at protein level, and this requires the import of the protein-level output from Proteome Discoverer. We read in the protein-level data and extract a vector of all protein accessions (from the Accession column) that have a Protein.FDR.Confidence.Combined value of "High". This corresponds to proteins with an FDR < 0.01 and we call this vector confidentProteins. Next, we use the filterFeatures function to keep only PSMs that have a Master.Protein.Accessions value which matches to our confidentProteins vector. Alternative software may provide information about the protein-level FDR at a lower data level. Users completing a more exploratory analysis may wish to use a less stringent protein-level FDR of 0.05 (corresponding to 5% false discovery rate).

## Import protein-level data
protein_data <- read.delim(file = "a549_uv_lopit_proteins.txt")

## Extract highly confident proteins
confidentProteins <- protein_data %>%
  filter(Protein.FDR.Confidence.Combined == "High") %>%
  pull(Accession)

## Filter to remove PSMs corresponding to proteins with an FDR > 0.01
qf <- filterFeatures(qf,
                     ~ Master.Protein.Accessions %in% confidentProteins,
                     i = sets_to_filter)



### Data-dependent quality control filtering

To further improve the quality of the data we complete some additional data-dependent quality control filtering. The quality control parameters that are available to filter here on will vary depending upon the type of experiment completed (DDA vs. DIA and label-based vs. label-free), the third-party software used for the identification search, and the data level (PSM vs. peptide vs. protein).

Here we follow the guidelines from Hutchings et al. ^20^ for TMT data processed using Proteome Discoverer. We control the (1) average reporter ion signal-to-noise (S/N) ratio, (2) percentage co-isolation interference, and (3) percentage SPS mass match. The thresholds set during these quality control steps will be dependent on the initial data quality, as well as the end goal being conservative vs. exploratory analysis. Further, exploration of quality may reveal differences in the data quality across samples, replicates or MS runs. In this case, users may consider setting different thresholds across samples to ensure that only high quality PSMs are retained. Users are encouraged to visualise the distribution of quality control parameters in their data before determining an appropriate threshold (see Quality Control Checks above).

As above, we use filterFeatures in QFeatures to filter the data. Here we chose to retain data with an isolation interference <= 75%, S/N >= 10, and an a SPS-MM >= 40%. Since all experimental replicates showed similar data quality, we here apply the same filters to all replicates.

## Data-dependent quality control filtering using filterFeatures
qf <- qf %>%
  filterFeatures(~ Isolation.Interference.in.Percent < 75, i = sets_to_filter) %>%
  filterFeatures(~ Average.Reporter.SN > 10, i = sets_to_filter) %>%
  filterFeatures(~ SPS.Mass.Matches.in.Percent > 40, i = sets_to_filter)



### Summary



qf


## An instance of class QFeatures containing 6 set(s):
##  [1] psms_raw_rep1: SummarizedExperiment with 115302 rows and 16 columns
##  [2] psms_raw_rep2: SummarizedExperiment with 135169 rows and 16 columns
##  [3] psms_raw_rep3: SummarizedExperiment with 120343 rows and 16 columns
##  [4] psms_filtered_rep1: SummarizedExperiment with 79117 rows and 16 columns
##  [5] psms_filtered_rep2: SummarizedExperiment with 90226 rows and 16 columns
##  [6] psms_filtered_rep3: SummarizedExperiment with 80446 rows and 16 columns



We started with 115302, 135169, 120343 PSMs for replicates 1, 2, 3, respectively, for both conditions prior to any filtering. Following cleaning and data-specific filtering we are left with 79117, 90226, 80446 PSMs across the three replicates.

### Subsetting individual samples

The rest of the data processing steps in this workflow need to be completed independently on each sample. This means that all steps must be done once per biochemical fractionation gradient. Thus, it is necessary to format the data into individual samples/gradients as we currently have both samples from each replicate stored in a single SummarizedExperiment. To split each replicate into its two corresponding samples ( unstim and xray) we need to subset the corresponding quantitative columns (TMT labels). The TMT labelling strategy is detailed below in Table 3.

**Table 3.** TMT labelling strategy for the use-case data.

TMT tag	Fraction	Condition
126	F1	Unstimulated
127N	F2	Unstimulated
127C	F3	Unstimulated
128N	F4	Unstimulated
128C	F5	Unstimulated
129N	F6	Unstimulated
129C	F7	Unstimulated
130N	SN	Unstimulated
130C	F1	Xray
131N	F2	Xray
131C	F3	Xray
132N	F4	Xray
132C	F5	Xray
133N	F6	Xray
133C	F7	Xray
134N	SN	Xray

We can split the data simply by subsetting the filtered experimental sets based on the indices of the required quantitative columns per sample. The information regarding which quantitative columns correspond to which condition per replicate (i.e., which sample) is stored in the colData that we generated earlier on. Since this experiment used the same TMT labelling strategy across all three replicate experiments, we can take the indices for unstimulated and x-ray treated fractions from the first replicate and used these to index all three of our experimental sets. We can then use this to construct a list of SummarizedExperiments corresponding to the individual conditions in each experiment before adding these back to the QFeatures object using the addAssay function.

## Get indices of quant columns per condition from colData
cond1 <- grep("unstim", colData(qf[["psms_filtered_rep1"]])$condition)
cond2 <- grep("xray", colData(qf[["psms_filtered_rep1"]])$condition)

## Subset individual samples (split replicates by condition)
conds <- list(
  psms_rep1_unstim = qf[["psms_filtered_rep1"]][, cond1],
  psms_rep2_unstim = qf[["psms_filtered_rep2"]][, cond1],
  psms_rep3_unstim = qf[["psms_filtered_rep3"]][, cond1],
  psms_rep1_xray = qf[["psms_filtered_rep1"]][, cond2],
  psms_rep2_xray = qf[["psms_filtered_rep2"]][, cond2],
  psms_rep3_xray = qf[["psms_filtered_rep3"]][, cond2]
)

## Add back to the QFeatures object
qf <- addAssay(qf, conds)



Next, we use filterNA to remove any PSMs which were not quantified in the given samples. This would be the case if a PSM was quantified in only one of the conditions within the TMTplex. We set a threshold pNA of 7/8 to remove PSMs with 8/8 missing values. Importantly, this is **not** the final missing value filtering of the dataset and we would not use PSMs with 7/8 missing values. The reason for completing this filtering is so that the dimensions of each experimental set (number of rows/PSMs) are representative of the sample. If we did not carry out this filtering then the experimental sets for samples of each condition within a replicate would have the same number of rows/PSMs, but some of these may not actually have been identified and quantified in both samples.

## Define sets to filter upon
to_filter <- grep("psms_rep", names(qf))

## Filter to remove PSMs not found at all in sample – correct dims
qf <- filterNA(qf, i = to_filter, pNA = 7/8)



When dealing with a Qfeatures object containing > 6 experimental sets, using qf to print a summary will display only the first and last three experimental sets by default. To get a summary for all experimental sets we can use the experiments function.

## Check experimental sets in qf object
experiments(qf)


## ExperimentList class object of length 12:
##  [1] psms_raw_rep1: SummarizedExperiment with 115302 rows and 16 columns
##  [2] psms_raw_rep2: SummarizedExperiment with 135169 rows and 16 columns
##  [3] psms_raw_rep3: SummarizedExperiment with 120343 rows and 16 columns
##  [4] psms_filtered_rep1: SummarizedExperiment with 79117 rows and 16 columns
##  [5] psms_filtered_rep2: SummarizedExperiment with 90226 rows and 16 columns
##  [6] psms_filtered_rep3: SummarizedExperiment with 80446 rows and 16 columns
##  [7] psms_rep1_unstim: SummarizedExperiment with 79100 rows and 8 columns
##  [8] psms_rep2_unstim: SummarizedExperiment with 90203 rows and 8 columns
##  [9] psms_rep3_unstim: SummarizedExperiment with 80412 rows and 8 columns
##  [10] psms_rep1_xray: SummarizedExperiment with 79101 rows and 8 columns
##  [11] psms_rep2_xray: SummarizedExperiment with 90203 rows and 8 columns
##  [12] psms_rep3_xray: SummarizedExperiment with 80412 rows and 8 columns



We see that the last six experimental sets now represent individual samples or correlation profiling experiments, here with eight biochemical fractions each.

We could also use the ncols, nrows and dims functions on our Qfeatures object to return the number of columns (quantitative channels), rows (PSMs), or both for each of the experimental sets. For example, let’s look at the number of rows (PSMs) per experimental set.

## Check number of rows (PSMs) per experimental set in qf
nrows(qf)


##      psms_raw_rep1       psms_raw_rep2     psms_raw_rep3  psms_filtered_rep1
##             115302              135169            120343               79117
## psms_filtered_rep2  psms_filtered_rep3  psms_rep1_unstim    psms_rep2_unstim
##              90226               80446             79100               90203
##   psms_rep3_unstim      psms_rep1_xray    psms_rep2_xray      psms_rep3_xray
##              80412               79101             90203               80412



### Management of missing data

An important aspect of processing quantitative proteomics data is how to deal with missing data. Missing values are a recurrent issue in quantitative proteomics, and it is important to address the reasons why this missing data occurs. The reasons for incomplete data generally fall into two categories, (1) biological or (2) technical. The most common biological reason for missing data is simply that the peptide/protein is absent or exists in an intensity below the limit of MS detection. The pattern of missingness of these data is missing not at random (MNAR) but rather due to the intensity, for example, due to suppression in a particular biological system or condition. Technical reasons are also common and can be complex. Experiments that use data-dependent acquisition (DDA) mass spectrometry do not analyse all peptides in a sample. Peptides that are less abundant than some of their co-eluting ions, do not ionize well or do not get identified might be sporadically missing in the final quantitation table, despite their presence in the biological samples. Their absence patterns are missing (completely) at random (MAR or MCAR) in such cases.

In addition to understanding the types of missing values observed in general proteomics experiments, it is also useful to consider correlation profiling experiments specifically. Having carried out biochemical fractionation, missing values can still occur at random for the reasons outlined above but also tend to display patterns corresponding to the biochemical fractionation method used. In general, it is unlikely for a peptide to have a biological missing value (i.e., MNAR) in the middle of a fractionation gradient if it has been successfully quantified in the neighbouring fractions. However, it is possible, especially in experiments with a greater number of biochemical fractions, that peptides only be quantified in a subset of adjacent fractions and have MNAR values across the remainder of the gradient. This is because the organelle to which the corresponding protein localises may only be present in a few biochemical fractions. In LOPIT-DC datasets, for example, it is common to see an increasing proportion of missing values towards the end of the differential centrifugation gradient as most subcellular compartments have already pelleted, and only soluble cytoplasmic proteins are still quantified in the later fractions. Overall, whilst it is challenging to determine whether missing values are MAR or MNAR, visualation of missing values, as demonstrated below, should show a consistent pattern of missingness across samples and make sense given the biology of the biochemical fractionation method which was employed.

Importantly, missing values should be reported truthfully when processing data. We sometimes find that third party software used to generate quantitative data introduce zeros instead of properly reporting missing values. In Qfeatures there is a function to explicitly handle this situation called zeroIsNA() which finds all values which are 0 and replaces them with NA values. Similarly, infIsNA() can be used to replace infinite values by NA, if for example, third party software has divided expression data by zero values during custom normalisation.

### Exploring missing data

The first step in dealing with missing data is to explore the patterns of missing data in the experiment. To do so, we first convert any zero values to NA using zeroIsNA. We wish to apply this to all of the experimental sets in our Qfeatures object, even the raw data.

## Store the names of all experimental sets
all_sets <- qf %>%
  names()

## Convert zero values to NA
qf <- zeroIsNA(qf,
                i = all_sets)



Next, we use the nNA function in Qfeatures to examine missing values. We pass the name of the experimental set we are interested in looking at to the i = argument. We advise users to explore and deal with missing data on a per sample basis. To take a look at the presence and distribution of missing values per sample, we use lapply to apply the nNA function to each of our samples. The output of running nNA on each experimental set is stored in the object na_stats.

## Extract indices of experimental sets with names "psms_rep[any number]_"
ind <- grep("psms_rep[0-9]_", names(qf))

## Extract missing value information for each of these sets
na_stats <- lapply(ind, function(z) nNA(qf[[z]]))



Let’s take a look at replicate 1 of the unstimulated experiments.

na_stats[[1]]


## $nNA
## DataFrame with 1 row and 2 columns
##         nNA        pNA
##   <integer>  <numeric>
## 1      4775 0.00754583
##
## $nNArows
## DataFrame with 79100 rows and 3 columns
##              name       nNA       pNA
##       <character> <integer> <numeric>
## 1               2         0     0.000
## 2               8         3     0.375
## 3               9         0     0.000
## 4              12         1     0.125
## 5              13         0     0.000
## …               …         …         …
## 79096      115276         5     0.625
## 79097      115282         0     0.000
## 79098      115283         0     0.000
## 79099      115284         0     0.000
## 79100      115287         1     0.125
##
## $nNAcols
## DataFrame with 8 rows and 3 columns
##          name       nNA        pNA
##   <character> <integer>  <numeric>
## 1    rep1_126        96 0.00121365
## 2   rep1_127N       155 0.00195954
## 3   rep1_127C       132 0.00166877
## 4   rep1_128N       101 0.00127686
## 5   rep1_128C       293 0.00370417
## 6   rep1_129N       769 0.00972187
## 7   rep1_129C       843 0.01065740
## 8   rep1_130N      2386 0.03016435



The output of nNA contains several useful summary statistics. Firstly, looking at the nNA DataFrame gives us information about the global missing values for this sample. We get information about the total number ( nNA) and proportion ( pNA) of missing values. We see that we have 4775 PSMs which contain >= 1 NA value. Similarly, pNA tells us this equates to 0.0075 of the replicate 1 unstimulated data. If we look below, nNArows gives us information about the rows i.e. the PSMs. It tells us the number ( nNA) and proportion ( pNA) of NAs per PSM. Finally, nNAcols gives us information on the columns i.e. the TMT channels/fractions. As illustrated below, we have 8 channels, and the nNA column tells us the number of PSMs per channel with an NA, whilst pNA indicates the proportion of PSMs per channel with missing values.

We can examine the MVs and see if there is any pattern(s).

## Generate mv barplot for each of the 6 experimental sets
for(i in seq_along(na_stats)) {

  # Re-name pNA column so we have this information ready for plotting
  names(na_stats[[i]]$nNAcols$nNA) <- na_stats[[i]]$nNAcols$name

  # Plot the data
  print(
    na_stats[[i]]$nNAcols %>%
    as_tibble() %>%
    ggplot(aes(x = name, y = (pNA * 100))) +
      geom_col() +
      ylim(c(0, 100)) +
      labs(x = "Fraction", y = "Missing values (%)") +
      ggtitle(names(qf)[ind][i]))
}



**Figure 5.**
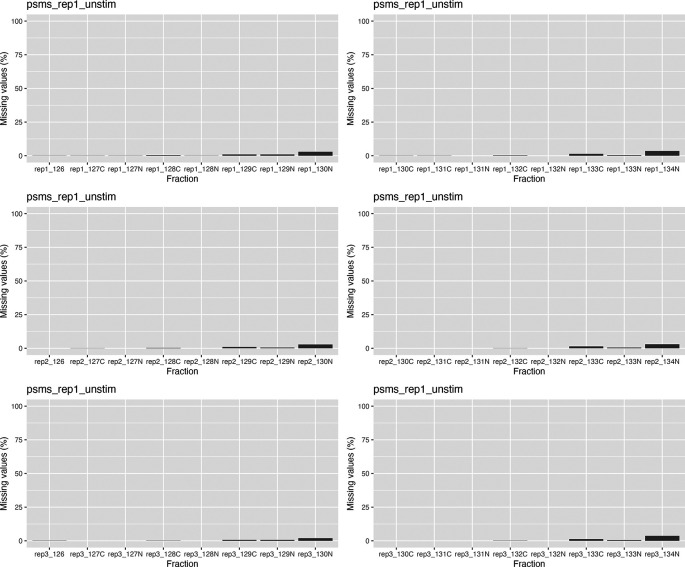
Barplots of missing values for each replicate in each condition across the fractionation gradient.

We can see from the barplots ( Figure 5) that we have very low percentage of missing values in general and that missing values tend to appear at the tail end of the gradient in the cytosolic fractions. This indicates that these values may be biological, as mentioned previously.

Importantly, no fractions/channels have an unusually high percentage of missing values and the pattern of missingness is consistent across samples. If users with find that a particular fraction or sample has a large percentage of missing values, and that this does not seem to represent a biological pattern, this could indicate that something went wrong during sample preparation. For example, labeling of one fraction may have failed, the fraction could have had less material or, for label-free datasets, a single MS run may have experienced problems. In such cases, it is possible to remove individual fractions at this point. However, it is advisable to remove the same fractions from all samples, especially if the downstream goal is to carry out differential localisation analysis. Removing fractions from only a subset of datasets will ultimately result in differential resolution of the samples and could bias downstream analyses.

**An additional note on label-based vs. label-free missing values:** The use-case data presented here used TMT-based peptide quantitation. When using a DDA TMT approach, TMT co-isolation interference often results in very low abundance values where there should really be a missing value. As a result, despite the potential for MNAR values due to biochemical fractionation, TMT correlation profiling based experiments tend to have a low proportion of missing data. As demonstrated below, this means that the datasets require minimal filtering and imputation. By contrast, label-free datasets have a much high proportion of missingness. This is the case for several reasons. Firstly, label-free samples are each run independently on the MS rather than being multiplexed as is done for label-based methods. For DDA label-free experiments this means that different peptides are measured across different samples, thus leading to lots of missing values. This effect is seen in all DDA label-free proteomics experiments but is even more dramatic for correlation profiling data as most approaches rely on selecting the top N most abundant precursor peptides for analysis, but the most abundant peptides are expected to be different across fractions of the biochemical gradient. In DIA label-free correlation profiling experiments missingness is not due to the stochastic selection of precursors ions but rather due to the fact that missing values present as truly missing, with no co-isolation interference to buffer the effect of biochemical fractionation. As a result of greater missingness, users should spend more time testing combinations of filtering and imputation for label-free correlation profiling experiment data, as discussed further in the sample DIA workflow (“Using this workflow with DIA-NN data”) presented in the appendix.

### Removing of data with missing values

We choose to allow up to two missing values per PSM. Typically, for label-based DDA correlation profiling datasets we recommend removing features (PSMs, peptides or proteins) with > 20-30% MVs. The exact number of acceptable MVs will depend upon the number of biochemical fractions. Across our eight biochemical fractions, we will allow two channels to contain NA values. We use the filterNA function on each experimental set and specify the threshold proportion of missing values whereby rows (PSMs) with a proportion above this threshold are removed from the data. Here, we use pNA = 2/8. The missing value threshold is ultimately to be decided by the user.

## Extract indices of experimental sets with names "psms_rep[any number]_"
ind <- grep("psms_rep[0-9]_", names(qf))

## Filter and allow 2 MV using filterNA
qf <- filterNA(qf,
               i = ind,        # the indices of our final subset samples
               pNA = 2/8)



Let’s check we have properly filtered out any PSMs with > 2 MV’s per replicate.

## Extract updated missing values information with nNA function
na_stats_updated <- lapply(ind, function(z) nNA(qf[[z]]))

## Check we have a maximum of only 2 MVs
sapply(na_stats_updated, function(z) max(z$nNArows$nNA)) %>%
  head()


## [1] 2 2 2 2 2 2



As expected, we can see that we have a maximum of two NA values remaining. We are now left with our final list of PSMs per sample.

### Data transformation and aggregation

In our previous expression proteomics workflow, we described the processing steps required to prepare quantitative proteomics data for differential abundance analysis. ^20^ Such preparation involved log2 transformation of the PSM-level quantitation data before aggregating to the protein level and normalising. These steps differ from those required when processing subcellular spatial proteomics data.

Quantitative proteomics data often undergoes logarithmic transformation prior to statistical analysis. The reason for this is that the majority of statistical tests require the data to display a normal distribution. Whilst raw quantitative MS-based proteomics data is dramatically skewed towards 0, the log2 transformed data is approximately normal. In this subcellular spatial proteomics workflow, however, the goal is not to prepare our data for downstream statistical differential abundance analysis. Instead, we are preparing the data for a machine learning classifier for which the input is protein correlation profiles across a biochemical gradient. The machine learning algorithm which we will use does not make the assumption that our data has a normal distribution, therefore we do not need to perform logarithmic transformation. In fact, logarithmic transformation would reduce the subcellular resolution by flattening our abundance distribution profiles.

The second data processing step which differs between expression and spatial proteomics analysis is that of normalisation. In most proteomics experiments the goal of normalisation is to remove any technological variation and reverse experimental error. As a result, normalised data should contain near-identical samples in which most variation is of biological importance. In spatial proteomics normalisation plays a different role because we are interested in the shape of protein abundance profiles rather than the magnitude of the intensity in each channel. Hence, the aim of normalisation here is to scale all our quantitation data into the same space (within the range of 0 and 1) whilst maintaining the shape of our abundance profiles.

### Normalisation

Based on De Duve’s principle ^21^ we know that proteins which are co-localised within the cell will exhibit similar abundance distribution profiles across a fractionation gradient. This means that we we can infer the location of proteins by matching their abundance profiles to profiles of well-known organelle residents, termed ‘markers’. In order to get information about the abundance distribution of each protein across the fractionation gradient we need to carry out row sum normalisation. To do this we make use of the normalize function within the QFeatures infrastructure.

## Extract indices of final psm sets we wish to normalise
ind <- grep("psms_rep", names(qf))

## Define the names of new normalised sets
n <- paste0(names(qf)[ind], "_norm")

## Apply normalisation to psm experimental set of each sample
qf <- normalize(qf,
                i = ind,
                name = n,
                method = "sum")

## verify
qf


## An instance of class QFeatures containing 18 set(s):
##  [1] psms_raw_rep1: SummarizedExperiment with 115302 rows and 16 columns
##  [2] psms_raw_rep2: SummarizedExperiment with 135169 rows and 16 columns
##  [3] psms_raw_rep3: SummarizedExperiment with 120343 rows and 16 columns
##  …
##  [16] psms_rep1_xray_norm: SummarizedExperiment with 78794 rows and 8 columns
##  [17] psms_rep2_xray_norm: SummarizedExperiment with 89910 rows and 8 columns
##  [18] psms_rep3_xray_norm: SummarizedExperiment with 80099 rows and 8 columns



We can see what this row sum normalisation has done to the quantitative data by looking at the assay data.

## Pre-normalisation
qf[["psms_rep1_unstim"]] %>%
  assay() %>%
  head()


##    rep1_126  rep1_127N  rep1_127C  rep1_128N  rep1_128C  rep1_129N  rep1_129C
## 2      19.4       52.0       67.2      105.1       86.6       31.5        6.7
## 9      24.2       28.7       20.4       14.5       20.2        6.6        6.3
## 12     49.6       71.3       98.9      145.6      102.9       44.3        4.0
## 13     21.2       33.6       28.0       34.4       39.3       71.1       61.1
## 17     55.8       65.0       37.8       29.2       25.5       60.5       70.4
## 18     33.3       37.4       27.2       24.2       34.8       65.1       66.7
##    rep1_130N
## 2       14.3
## 9       48.9
## 12        NA
## 13       3.8
## 17      34.3
## 18       5.7


## Post-normalisation
qf[["psms_rep1_unstim_norm"]] %>%
  assay() %>%
  head()


##      rep1_126 rep1_127N rep1_127C  rep1_128N rep1_128C rep1_129N    rep1_129C
## 2  0.05067921 0.1358412 0.1755486 0.27455590 0.2262278 0.08228840 0.017502612
## 9  0.14252061 0.1690224 0.1201413 0.08539458 0.1189635 0.03886926 0.037102473
## 12 0.09601239 0.1380178 0.1914441 0.28184282 0.1991870 0.08575300 0.007742935
## 13 0.07247863 0.1148718 0.0957265 0.11760684 0.1343590 0.24307692 0.208888889
## 17 0.14742404 0.1717305 0.0998679 0.07714663 0.0673712 0.15984148 0.185997358
## 18 0.11311141 0.1270380 0.0923913 0.08220109 0.1182065 0.22112772 0.226562500
##     rep1_130N
## 2  0.03735632
## 9  0.28798587
## 12         NA
## 13 0.01299145
## 17 0.09062087
## 18 0.01936141



Through the process of row sum normalisation we have now converted our raw quantitative values into a correlation profile across the fractionation gradient. The abundance values represent the proportion of total quantification for each PSM that comes from each fraction, with each row sum being equal to 1.

### Imputation

As we have discovered above, our filtered normalised PSM datasets still contain some missing (NA) values. This is to be expected since we only removed PSMs that had >2 missing values. The number and proportion of remaining PSMs which are missing quantitation values across the gradient will depend on the exact experimental design. For example, LFQ methods will generate a greater extent of missing values than many label-based technologies. Similarly, DDA label-free datasets are expected to display more missing values than their DIA equivalents.

Imputation has been discussed in our previous workflow ^20^ and we have been careful to not make too many recommendations but instead allow users to make their own decisions on whether to allow missing values. It is also important to consider if missing data is biological or technical. When exploring the pattern of missing values in our datasets we noted that we have very few missing values and those that do exist tend to appear at the end of each gradient, thus suggesting that they are missing for biological reasons. Hence, if we were to remove these PSMs from our data we could be losing biologically relevant information. Therefore, we here demonstrate how to impute the remaining missing values for users who wish to take this approach. Alternatively, users with a low proportion of missing values may also choose to simply remove features (here PSMs) with missing data.

Imputation of the data can be achieved using the impute function within the QFeatures infrastructure. A range of imputation methods are available within this function, and it is even possible to use multiple methods to generate a mixed imputation strategy.

## Which methods are available for imputation within impute()?
MsCoreUtils::imputeMethods()


##  [1]  "bpca"  "knn"   "QRILC"  "MLE"    "MLE2"  "MinDet"  "MinProb"
##  [8]  "min"   "zero"  "mixed"  "nbavg"  "with"  "RF"      "none"



As discussed in Hutchings et al., ^20^ the best imputation method to use will depend on the exact reason that the data are missing. Here, we demonstrate the application of a simple k-Nearest Neighhbours (k-NN) imputation approach.

## Extract indices of normalised sets we wish to impute
ind <- grep("norm", names(qf))

## Define the names of new imputed sets
n <- gsub("_norm", "_imputed", names(qf)[ind])

## Apply kNN imputation to normalised psm experimental set of each sample
set.seed(12345)
for (z in seq_along(ind)) {
  set.seed(1)
  qf <- QFeatures::impute(qf,
                          i = ind[z],
                          method = "knn",
                          name = n[z])
}


## Verify
qf


## An instance of class QFeatures containing 24 set(s):
##  [1] psms_raw_rep1: SummarizedExperiment with 115302 rows and 16 columns
##  [2] psms_raw_rep2: SummarizedExperiment with 135169 rows and 16 columns
##  [3] psms_raw_rep3: SummarizedExperiment with 120343 rows and 16 columns
##  …
##  [22] psms_rep1_xray_imputed: SummarizedExperiment with 78794 rows and 8 columns
##  [23] psms_rep2_xray_imputed: SummarizedExperiment with 89910 rows and 8 columns
##  [24] psms_rep3_xray_imputed: SummarizedExperiment with 80099 rows and 8 columns



**An additional note on imputing before or after normalisation:** As discussed above, the use-case data has a relatively low proportion of missing values. As a result, after an initial filtering step, there are not many values to impute. Here, we demonstrate a simple k-NN imputation method. Since we have previously found k-NN imputation to be more successful when the data are in a similar space, we here applied imputation after normalisation. We note that by doing this, the final imputed profiles will no longer sum to exactly 1. However, since we have a maximum of two imputed values per PSM, the sum should not greatly exceed 1. By contrast, where datasets have a greater proportion of missing data to impute, row sum normalisation of a limited number of fractions would generate a short profile summed to one and subsequent imputation could result in a correlation profile with an undesirably high sum value. This is the case for the data used in the sample DIA workflow (“Using this workflow with DIA-NN data”) presented in the appendix. As discussed in the appendix, here it would likely be necessary to impute prior to normalisation to ensure the generation of sensible correlation profiles in the same final space (between 0 and 1).

### Aggregation

Now that we have our final PSM-level dataset we can aggregate this upward to the protein level. The aim of this step is to combine data from all component PSMs into a single protein-level entry. This entry will have one master protein accession and one quantitative value per biochemical fraction per sample.

Within the QFeatures infrastructure aggregation is carried out using the aggregateFeatures function. This function provides users with several options on how to aggregate multiple quantitation values into a single value. The methods differ with respect to how they deal with missing data as well as whether they require log2 or raw quantitation data as their input.

In our sister workflow ^20^ we aggregated using robustSummary, a state-of-the-art aggregation method which is notably robust to outliers and missing values. ^22, 23^ However, our data is not currently suitable for robustSummary aggregation as this method requires log2 transformed data. Whilst it would be relatively simple to log2 transform our quantitative data using the logTransform function within the QFeatures infrastructure, we would need to reverse this transformation by taking the exponential of the resulting protein data, which is not so simple. Instead, we will aggregate PSMs straight to protein level using colMedians method from the matrixStats package. We specify this method using the fun = argument, as well as telling the function which rowData column we wish to use for aggregation.

Here, we demonstrate one-step aggregation, from PSM directly to protein. This means aggregating all PSMs with the same value of "Master.Protein.Accession". For an example of two-step aggregation, from PSM to peptide followed by peptide to protein, users are directed to Hutchings et al. ^20^

## Extract indices of imputed normalised psm sets we wish to aggregate
ind <- grep("imputed", names(qf))

## Define the names of new protein-level sets
n <- c(paste0("prots_rep", 1:3, "_unstim"),
       paste0("prots_rep", 1:3, "_xray"))

## Aggregate from psm to protein
for (z in seq_along(ind)) {
  qf <- QFeatures::aggregateFeatures(qf,
                                     i = ind[z],
                                     fun = matrixStats::colMedians,
                                     name = n[z],
                                     fcol = "Master.Protein.Accessions")
}

qf


## An instance of class QFeatures containing 30 set(s):
##  [1] psms_raw_rep1: SummarizedExperiment with 115302 rows and 16 columns
##  [2] psms_raw_rep2: SummarizedExperiment with 135169 rows and 16 columns
##  [3] psms_raw_rep3: SummarizedExperiment with 120343 rows and 16 columns
##  …
##  [28] prots_rep1_xray: SummarizedExperiment with 6446 rows and 8 columns
##  [29] prots_rep2_xray: SummarizedExperiment with 6689 rows and 8 columns
##  [30] prots_rep3_xray: SummarizedExperiment with 6374 rows and 8 columns



We have now populated the QFeatures object with protein-level data containing the row normalised protein abundance profiles. From looking at the summary in experiments(qf ) we have between 6000 and 7000 proteins in each sample. These protein correlation profiles contain spatial information and are the input to downstream machine learning classifers.

### Concatenating datasets for machine learning

Previous subcellular proteomics experiments utilising protein correlation profiles have benefited from the combining of datasets, ^3, 24, 25^ that is the concatenation of replicates or experiments that utilise different gradients. ^26^ Since the use-case experiment included three biological replicates each with 8 biochemical fractions we can concatenate each condition into a single dataset with 24 columns. In order to do this, the column names must be different in each replicate. By looking at the colnames of each experimental set per condition we verify we indeed have unique names (annotated by the prefix “repx” appended during import). If the colnames were not unique to each replicate we would have to use the renameColname function, specifying which experimental set we wish to change the column names of ( i = argument) and then providing a vector of new column names ( value = argument).

colnames(qf[["prots_rep1_unstim"]])


## [1] "rep1_126"  "rep1_127N" "rep1_127C" "rep1_128N" "rep1_128C" "rep1_129N"
## [7] "rep1_129C" "rep1_130N"


colnames(qf[["prots_rep2_unstim"]])


## [1] "rep2_126"  "rep2_127N" "rep2_127C" "rep2_128N" "rep2_128C" "rep2_129N"
## [7] "rep2_129C" "rep2_130N"


colnames(qf[["prots_rep3_unstim"]])


## [1] "rep3_126"  "rep3_127N" "rep3_127C" "rep3_128N" "rep3_128C" "rep3_129N"
## [7] "rep3_129C" "rep3_130N"



The experimental sets are concatenated using the joinAssays function in QFeatures.

## Combine replicates - each with unique column names
qf <- joinAssays(x = qf,
                 i = c("prots_rep1_unstim",
                       "prots_rep2_unstim",
                       "prots_rep3_unstim"),
                  name = "prots_unstim")

qf <- joinAssays(x = qf,
                 i = c("prots_rep1_xray",
                       "prots_rep2_xray",
                       "prots_rep3_xray"),
                  name = "prots_xray")

## Keep only proteins found across all three replicates
qf <- filterNA(qf, i = "prots_unstim")
qf <- filterNA(qf, i = "prots_xray")

## Verify
qf


## An instance of class QFeatures containing 32 set(s):
##  [1] psms_raw_rep1: SummarizedExperiment with 115302 rows and 16 columns
##  [2] psms_raw_rep2: SummarizedExperiment with 135169 rows and 16 columns
##  [3] psms_raw_rep3: SummarizedExperiment with 120343 rows and 16 columns
##  …
##  [30] prots_rep3_xray: SummarizedExperiment with 6374 rows and 8 columns
##  [31] prots_unstim: SummarizedExperiment with 5701 rows and 24 columns
##  [32] prots_xray: SummarizedExperiment with 5700 rows and 24 columns



We see that we have two new experimental sets, one for each condition. The dataset prots_unstim has 24 quantitation channels and contains 5701 proteins common across the three replicates. The dataset prots_xray has 5700 proteins across the three replicates for the 12hr-xray stimulated dataset.

### Concatenation gives equal weight to all replicates

By concatenating the datasets we give each biological replicate an equal weight for the downstream machine learning classification. This means that any variability between experiments will be accounted for. For example, if a protein has an abundance distribution profile similar to that of nuclear protein markers in one of the three replicates but is more similar to cytoplasmic markers in the remaining two replicates, this will be reflected by the fact that the protein will either be classified with a lower confidence or not classified at all. If each replicate was analysed separately, the protein may receive conflicting classifications in each replicate which would require a user-defined resolution.

Another potential advantage of concatenating datasets is the inclusion of data derived from different gradient variations. ^24, 26^ Although many subcellular compartments are well resolved using a single gradient, some compartments remain more challenging to resolve due to their similarities or connections with other compartments. For example, compartments within the endomembrane system can generate similar fractionation profiles. Where no single gradient will provide optimal separation of all organelles of interest, it is possible to design different gradients which provide optimal separation different organelles. These data can then be concatenated to generate a combined dataset with greater final resolution of all desired compartments.

Overall, data concatenation has been shown to have benefits for downstream machine learning in many cases. Ultimately, however, the decision to concatenate replicates will depend on the goal of the experiment and type of data analysis required to answer the experimental question. Where the experimental aim is to identify differential localisation between conditions it is recommended to repeat the same biochemical gradient across all three replicates to avoid complicating downstream analysis.

## Part 2: Protein localisation via supervised machine learning

Now that we have generated our final dataset containing protein correlation profiles, we can use this data to generate spatial maps. Specifically, in part 2 of this workflow we will discuss how to determine the steady-state localisation of proteins utilising classical or Bayesian supervised machine learning algorithms. To do so we will focus on the concatenated unstimulated A549 dataset and show how to generate a static spatial map from this data. In reality, we advise users to carry out classification on each individual replicate, as well as any concatenated maps.

All protein correlation profiling methods, including LOPIT, are based on the fractionation patterns of different organelles across a biochemical gradient. Patterns of proteins which co-fractionate together are indicative of proteins which co-localise together within the cell. ^27^ The computational analysis of such methodologies requires us to (1) identify distinct patterns of protein co-fractionation, and (2) associate each identified fractionation pattern with marker proteins known to reside in a particular subcellular compartment. Hence, we predict protein localisation by comparing the correlation profiles of query proteins to those of marker proteins with a known subcellular localisation.

We will first discuss the definition of marker proteins, that is proteins which are known to consistently localise to a single subcellular niche in a given cell type *and* under given conditions. The correlation profiles of the selected marker proteins will then be used to train a supervised machine learning classifier. The output of this algorithm will be a predicted subcellular localisation for each of our proteins. Finally, we show how to visualise the predicted protein localisations to generate a spatial map of the cell.

### Extract protein-level data into MSnset infrastructure for use in pRoloc

This section of the workflow requires specialised functions for data visualisation and machine learning. Such functions exist within the pRoloc package. ^28^ To use the functions within pRoloc we need our data to be stored in an MSnSet rather than a QFeatures object. Although there is currently no function available to directly convert a QFeatures object into an MSnSetList, we can use the as function to coerce each experimental set (or SummarizedExperiment) of our QFeatures object into an MSnSet.

Since this part of the workflow will demonstrate how to determine protein localisastion using the control unstimulated condition, we here coerce our prots_unstim experimental set into a MSnSet.

## Coerce each experimental set of interest into an MSnSet
unstim_msn <- as(object = qf[["prots_unstim"]], Class = "MSnSet")

## Verify
unstim_msn


## MSnSet (storageMode: lockedEnvironment)
## assayData: 5701 features, 24 samples
##   element names: exprs
## protocolData: none
## phenoData
##   sampleNames: rep1_126 rep1_127N … rep3_130N (24 total)
##   varLabels: runCol quantCols condition
##   varMetadata: labelDescription
## featureData
##   featureNames: A0A0B4J2F0 A0A0U1RRE5 … Q9Y6Y8 (5701 total)
##   fvarLabels: Checked Tags … Number.of.Protein.Groups (22 total)
##   fvarMetadata: labelDescription
## experimentData: use 'experimentData(object)'
## Annotation:
## - - - Processing information - - -
##  MSnbase version: 2.34.0



As a reminder for readers, the structure of an MSnSet object is very similar to that of a SummarizedExperiment, or QFeatures experimental set. The assay slot of a SummarizedExperiment is equivalent to that of the MSnSet exprs slot, the rowData is equivalent to the MSnSet fData, and the colData is the pData of an MSnSet. Hence, the remainder of this workflow will use the MSnbase exprs, fData and pData functions rather than the QFeatures assay, rowData and colData functions. The relationship between these QFeatures and MSnSet functions are summarised in Table 1.

**Peptide-level subcellular spatial mapping:** The majority of correlation profiling experiments aim to generate one or more protein-level cell maps. Therefore, previous data analysis workflows, including our first F1000 workflow for spatial proteomics, ^11^ started with the protein-level data output from a database search. However, it is also possible to generate cell maps at the peptide or even PSM/precursor level. In particular, peptide-level spatial maps have the potential to provide information about the differential localisation of proteoforms by revealing cases where peptides corresponding to the same master protein accession behave differently. For instance, the modified and unmodified versions of peptide sequences can be mapped separately rather than being aggregating into a single protein-level entry. Further, peptide-level maps can be used for quality control to investigate how consistent the profiles and predicted localisations of peptides are for proteins with limited peptide support.

One of the advantages of processing the data from the lowest possible level (PSM-level for the use-case) within the QFeatures infrastructure is the ability to store and access all of these data levels in a single object. In part 1 of this workflow we aggregated our PSM data directly to protein by using the aggregateFeatures function to group PSMs based on their Master.Protein.Accessions. To get peptide-level information, we could have included an additional intermediate aggregation step, as demonstrated in Hutchings et al. ^20^ Here, we would first aggregate all PSMs into peptides by passing either Sequence (ignore modifications) or Modified.Sequence (include modifications) to the fcol argument in aggregateFeatures, and then all peptides into proteins by passing Master.Protein.Accessions. The peptide-level data could then be extracted into an MSnSet in the same way as demonstrated above. All functions used throughout the rest of the workflow could be equally applied to an MSnSet containing peptide-level data, although some functions may require longer to run given that the peptide-level data is larger than the corresponding protein-level dataset.

To generate peptide-level spatial maps using this workflow:

1. Aggregate from PSM to peptide within the QFeatures infrastructure
2. Extract peptide-level experimental set from QFeatures into an MSnSet
3. Continue to use the rest of the workflow in the same way as demonstrated for protein-level data

An example workflow for peptide-level mapping is provided in the appendix, which is available from Zenodo under the identifier doi: 10.5281/zenodo.15100485.

### Initial assessment of data resolution

Prior to the annotation of marker proteins, it is first necessary to assess the resolution of the data. The aim of this assessment is to verify that subcellular niches have been successfully separated along the biochemical fractionation gradient and that there are distinct patterns of co-fractionation present in the data.

### Visualisation using dimensionality reduction

One way in which the initial assessment of resolution can be made is by using dimensionality reduction methods to summarise and visualise the dataset. Rather than looking directly at protein correlation profiles, here comprised of 24 dimensions (24 biochemical fractions), we simplify the data and visualise it in a two-dimensional space. In this case, proteins which are localised to the same subcellular niche should have similar correlation profiles and cluster together within a two-dimensional space. For a more in-depth discussion regarding the interpretation of dimensionality reduction plots for correlation profiling data users are directed to Gatto et al. ^29^

Principal Component Analysis (PCA) and t-distributed Stochastic Neighbour Embedding (t-SNE) are two dimensionality reduction methods which are commonly used for the visualisation of spatial proteomics data. The plot2D and plot3D functions within pRoloc can be used to generate and visualise PCA and t-SNE data, along with other approaches to dimensionality reduction (all methods are listed in plot2Dmethods). We pass our unstim_msn object to plot2D and specify "PCA" or "t-SNE" as our method. We also specify fcol = NULL to ignore any feature data and prevent the colouring of features (proteins) based on any annotation. Finally, when using any method which requires random number generation, we include a set.seed function to ensure reproducibility. Without setting a seed we would get a different t-SNE plot each time the code is executed.

par(mfrow = c(1, 2))

## Visualise unlabelled data using PCA and t-SNE
unstim_msn %>%
   plot2D(fcol = NULL, col = "black", method = "PCA", main = "PCA")

set.seed(399)
unstim_msn %>%
  plot2D(fcol = NULL, col = "black", method = "t-SNE", main = "t-SNE")



**Figure 6.**
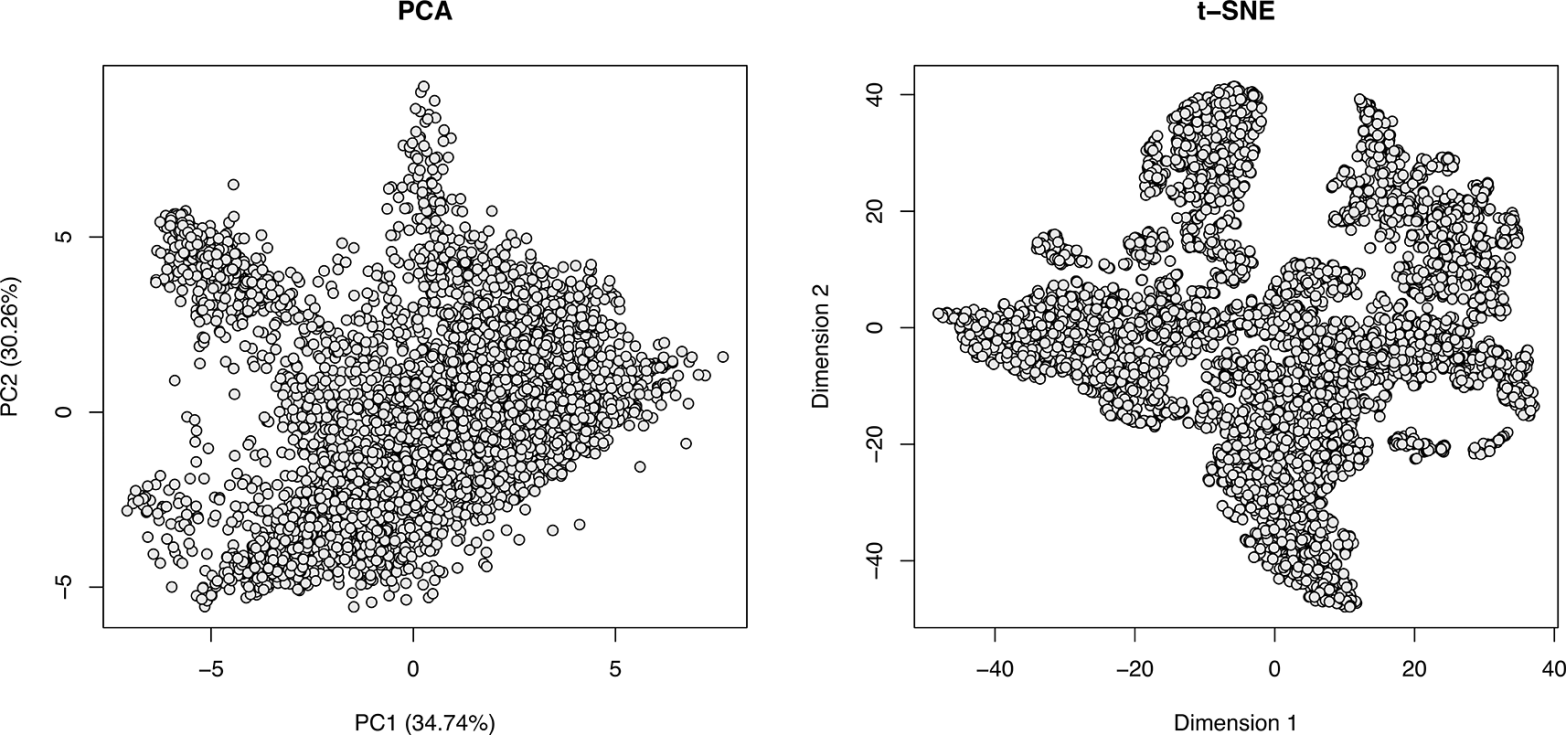
PCA (left) and t-SNE (right) plots of the unstimulated use-case data. Each point represents one protein.

By visualising the data without any annotation ( Figure 6) we can see evidence for the presence of clusters within our unstimulated A549 dataset. This is our first indication that the experiment has been successful and that different subcellular compartments have been separated along the fractionation gradient. At this point users are looking for the data to have shape and structure. If dimensionality reduction results in a mass of features in the middle of the plot with minimal shape or clusters, this indicates that (1) data pre-processing was not performed correctly e.g., a log2 transformation was applied, or (2) the experiment itself lacks resolution, potentially due to over-lysis of cells resulting in loss of subcellular structures prior to biochemical fractionation.

**An additional note on dimensionality reduction:** Dimensionality reduction methods are excellent visualisation tools for subcellular proteomics data. However, users should be careful when interpreting such figures since they only represent a simplified snapshot of the data. PCA, for example, uses a linear data transformation which means that distance can be directly interpreted (proteins closer together have more similar fractionation profiles). However, only two or three principal components can be visualised in a given plot meaning that clusters resolved in alternative principal components (i.e., dimensions) cannot be seen. The t-SNE and Uniform Manifold Approximation and Projection for Dimension Reduction (UMAP) methods, on the other hand, require non-linear transformation of the data which prevents interpretation of distance and can result in clustering artefacts ^30^ but tends to show more of the overall variation. Only visualisation of the protein correlation profiles themselves can provide a true picture of the data.

### Visualisation using heatmaps

In addition to dimensionality reduction, heatmaps are also a popular tool for visualising patterns and relationships in large datasets. To gain a snapshot of protein behaviour across different fractions and replicates we can use the pheatmap function from the pheatmap package to generate a clustered heatmap of the normalised protein correlation profiles. The input to pheatmap is the matrix of correlation profiles stored in the exprs slot of the MsnSet. By default, a hierarchical clustering is performed on the correlation profiles and the heatmap is ordered according to this clustering.

Since we wish to visualize the correlation profile of each protein in an intuitive order (that is the order of the biochemical fractionation gradient), we tell pheatmap not to cluster based on columns (quantitative channels) by setting cluster_cols to FALSE. This will retain the column order we have in our MsnSet rather than clustering similar columns together. In order to get some idea of which proteins (rows of the MsnSet) exhibit similar normalised abundance distributions and, therefore, could reside in the same subcellular compartment, we set cluster_rows to TRUE. This means that proteins (rows) with similar correlation profiles will be grouped together.

## Plot heatmap of protein correlation profiles – row clustering only
pheatmap(mat = exprs(unstim_msn),
         cluster_rows = TRUE,
         cluster_cols = FALSE,
         show_rownames = FALSE)



**Figure 7.**
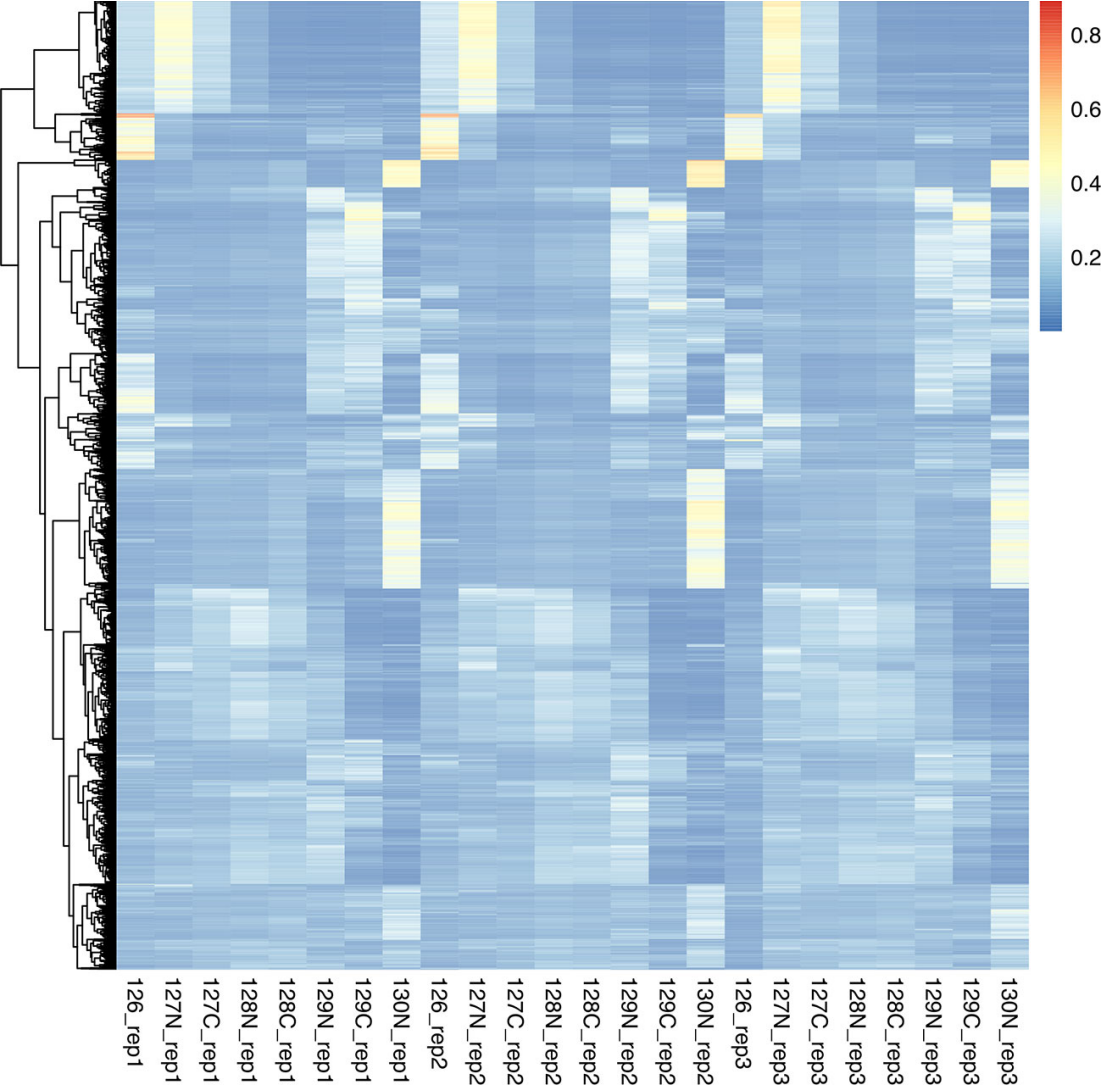
Heatmap of the correlation profiles from all replicates in the unstimulated data.

We can quickly see patterns of similarity emerging for proteins across fractions, and also across replicates, indicated by coloured blocks, as displayed in Figure 7. The colours reflect the normalised relative abundance for each protein in each fraction and replicate; blue indicating a low recorded normalised abundance and yellow, orange and red indicating a high normalised abundance value for a given protein. Protein (row) names are omitted for clarity. Blocks with different colours across columns are indicative of structured correlation profiles. For example, the top-most cluster is comprised of several hundred rows with a peak abundance in the second biochemical fraction. This pattern is unique to this cluster, providing confidence that these profiles will be resolved from others in the dataset and that this set of proteins could represent a subcellular niche. Depending on the experimental design we expect to see a similar trend between replicates in where proteins peak in abundance across the same fractions.

### Defining markers for machine learning

In the context of spatial proteomics data analysis, a marker is a protein known to reside in a single subcellular niche within a given system *and* under given conditions. In other words, markers are proteins which we can confidently assign to a known single localisation based on prior knowledge. In the context of protein localisation prediction, marker proteins constitute the labelled training data provided to the classification algorithm. Defining marker proteins can be challenging and time consuming, but it is critical to obtain a list of markers which are representative of the experiment and dataset of interest.

**Figure 8.**
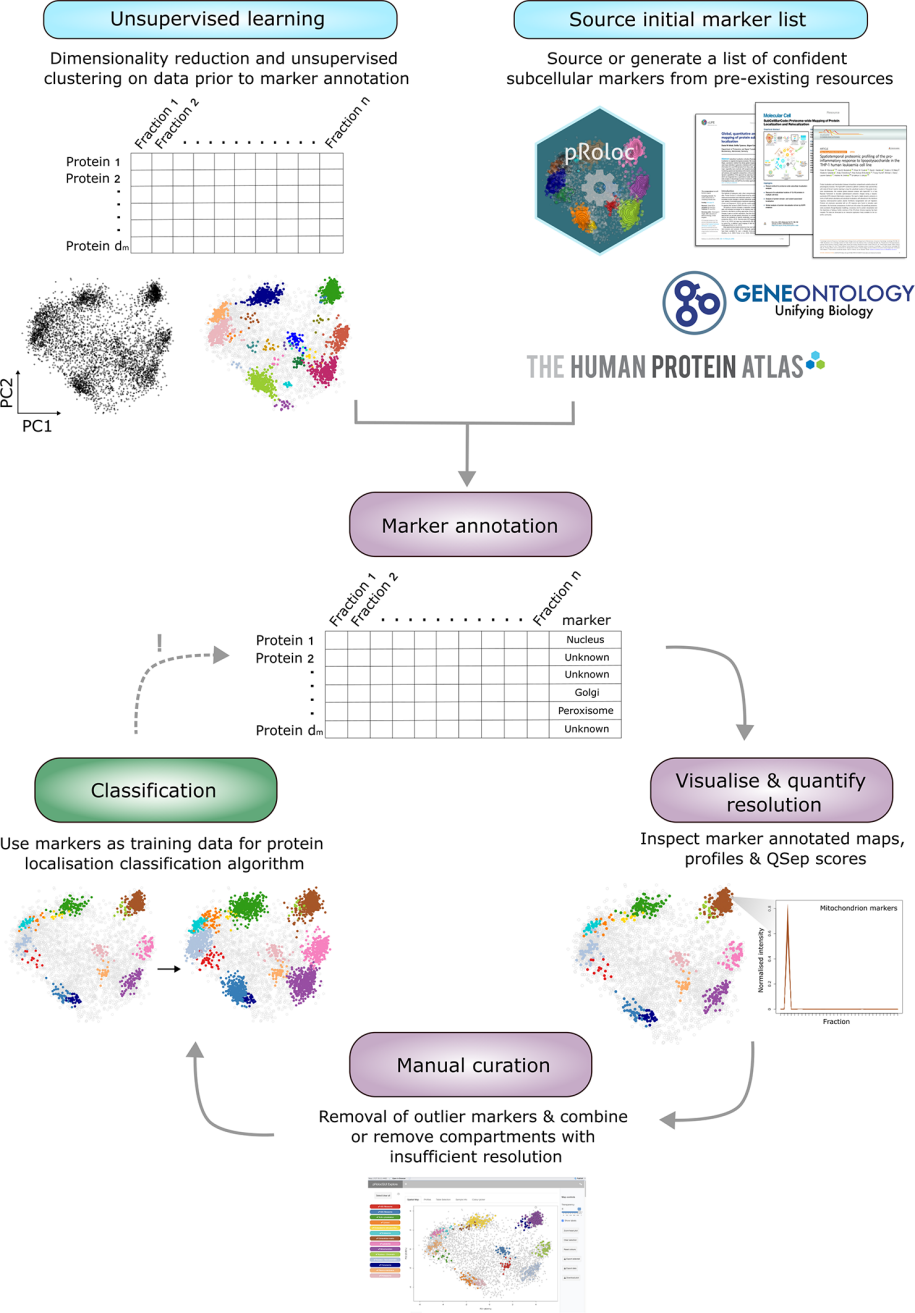
A graphical representation of the process of marker curation. Markers are proteins which are known to localise to a single subcellular compartment in the given cell type and condition of interest. The selection of confident markers is critical for successful prediction of protein localisation and differential localisation. (!) After using curated markers for protein localisation classification, users might still need to do some final curation e.g., if localisation prediction results do not look sensible (over- or under-classification of a particular organelle). However, users should be cautious when repeatedly refining markers lists as to ensure that the final markers remain representative of the literature and data structure.

The definition of subcellular markers can be broadly divided into five steps, with steps 3, 4 and 5 representing an iterative process, as outlined in Figure 8.

1. Determine which subcellular organelles have been sufficiently resolved in the data
2. Generate or source an initial marker list for the selected compartments in your organism
3. Evaluate the suitability of the marker list for your specific dataset
4. Update the marker list by removing outliers, combining compartments or expanding markers
5. Carrying out protein localisation classification using curated markers

### Step 1: Determining organelle resolution

Dimensionality reduction allows us to visualise our dataset and confirm the presence of clusters, thus indicating resolution of subcellular compartments. However, this information alone cannot tell us *which* subcellular niches we have resolved in our data.

When applying supervised machine learning to predict protein localisation the classifier is only able to allocate proteins to one of the subcellular locations included in the training data. Hence, the final classification results are intrinsically related to which subcellular compartments are provided to the classifier. Providing training data for subcellular compartments which are not sufficiently resolved or excluding compartments which are well resolved could lead to incorrect protein localisation allocations. Hence, it is necessary to carefully consider which subcellular compartments are included in the analysis.

**Unsupervised clustering: Hierarchical clustering with** hclust

Unsupervised learning via clustering analysis can be used to gain insight into which subcellular niches we have the ability to resolve in our data. At a basic level, clustering methods simply seek to find structure in an unlabelled dataset via grouping data points whose members are similar in some way. This step is particularly useful for those studying the spatial proteome of non-model organisms or aiming to discover new subcellular structures. Several unsupervised machine learning algorithms, including classic methods such as hierarchical clustering and *k*-means, are available in pRoloc via the MLInterfaces package, as outlined in the vignette Using pRoloc fo spatial protemics data analysis.

In the section above we carried out hierarchical clustering and visualisation using the pheatmap function. This allowed us to cluster rows and see groups of rows (proteins) with similar abundance profiles (correlation profiles). We can take this a step further by annotating the discovered clusters on our data using dimensionality reduction.

First, we regenerate the same heatmap as above with some additional annotations to make the individual clusters easier to see. Here, we choose to do the hierarchical clustering separately from the plotting so that we have all the data we need in an accessible object. The same hierarchical clustering carried out within the pheatmap function can also be completed using the hclust function from the stats package. We first pass the quantitative exprs data of our unstim_msn to the dist function to generate a distance matrix and then pass this distance matrix to hclust. We will store the results in an object called hc. Next, we use the cutree function to specify how many clusters we wish to split the data into. The most appropriate number will vary depending on the data size and resolution, so users should test a few options. Here, we demonstrate using 12 clusters since most high-quality correlation profiling experiments resolve 10-15 subcellular compartments.

## Carry out hierarchical clustering with hclust
hc <- unstim_msn %>%
  exprs() %>%
  dist() %>%
  hclust()

## Specify number of clusters (k) and extract proteins in each cluster
k <- 12
cl <- cutree(hc, k)



**Figure 9.**
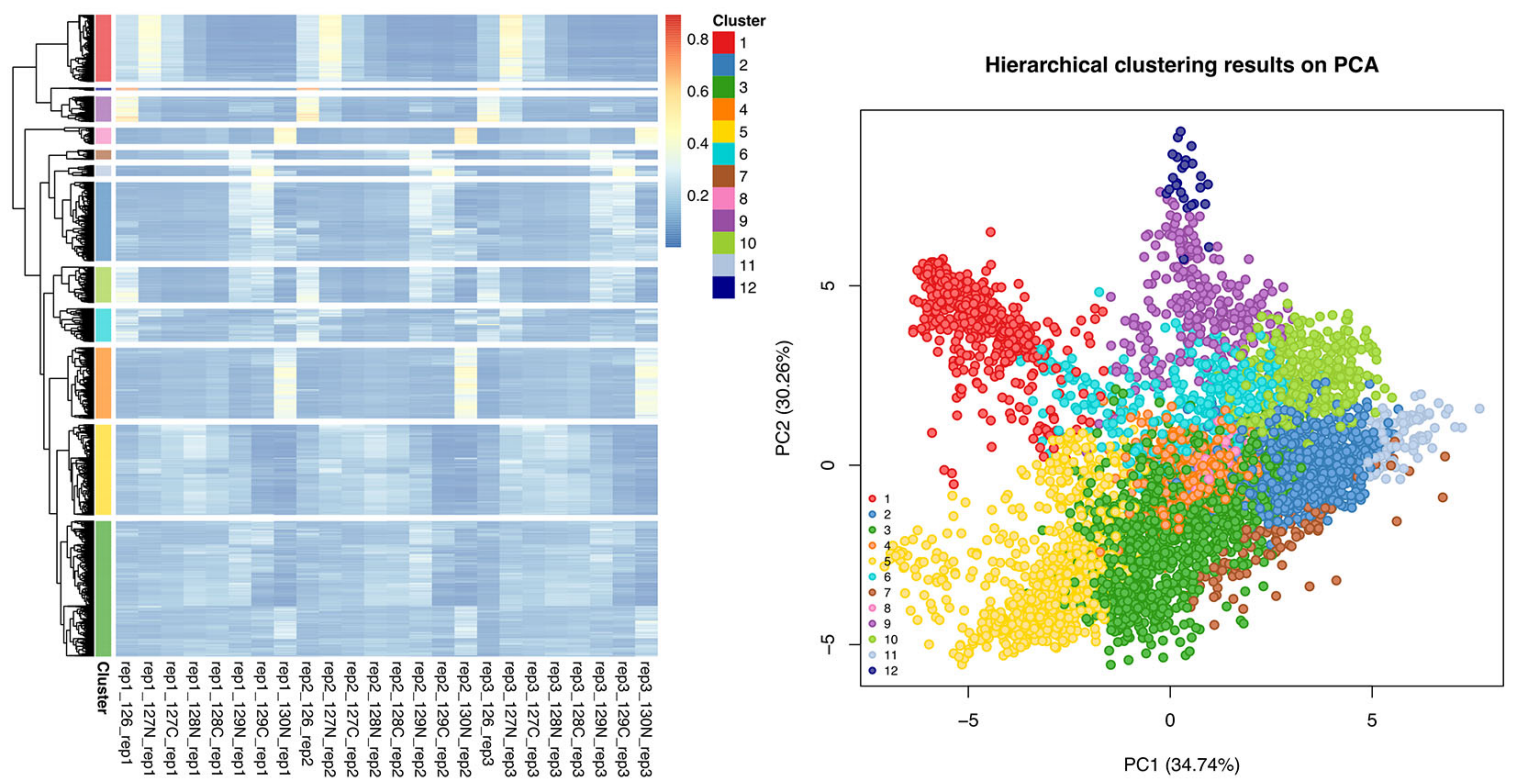
Results of hierarchical clustering carried out on protein correlation profiles using hclust.

Now that we have a named vector containing all our proteins with an associated hierarchical cluster number, we can regenerate our heatmap and annotate this information ( Figure 9, left). To do so, we generate a data.frame with a column called Cluster containing the cluster number and make sure that the rownames match the featureNames of our unstim_msn object. We also define our own colours to match the default colours used by plot2D such that our clusters have the same colour annotations in our heatmap and dimensionality reduction visualisation. Finally, we use the pheatmap function as above but pass our row annotation data.frame to annotation_row and our pre-defined colours to annotation_colors.

## Create a dataframe containing cluster annotations
cl_annot <- data.frame("Cluster" = factor(cl))

## Define colours (to be same as PCA below = default plot2D)
my_cols <- list("Cluster" = getStockcol()[1:k] %>% set_names(1:k))

## Plot heatmap with rows annotated by cluster number
pheatmap(mat = exprs(unstim_msn),
         cluster_rows = TRUE,
         cluster_cols = FALSE,
         show_rownames = FALSE,
         cutree_rows = k,
         annotation_row = cl_annot,
         annotation_colors = my_cols)

To plot these hierarchical clusters on a dimensionality reduction map, we first add the cluster information to the fData of our MSnSet. We can then use the plot2D function to generate PCA plots with the discovered clusters annotated ( Figure 9, right). As above, we pass our unstim_msn object and specify the dimensionality reduction method to use. We also tell the function which column of our fData to be used for colouring the points - here we use the newly created hc column.

## Add hierarchical cluster information to MSnSet fData
fData(unstim_msn)$hc <- cl

## Visualise hierarchical clusters on PCA
unstim_msn %>%
  plot2D(fcol = "hc", method = "PCA", main = "Hierarchical clustering results on PCA")

## Add legend
unstim_msn %>%
  addLegend(fcol = "hc", cex = 0.7)



**Unsupervised clustering: Hierarchical clustering with** hdbscan

Hierarchical DBSCAN (HDBSCAN) is another form of clustering which can be executed in R using the dbscan package. ^31^ This algorithm has been used to successfully uncover and validate clusters in subcellular spatial proteomics data generated from *Toxoplasma gondii* ^24^ and African trypanosomes. ^25^ Extensive documentation on the use of HDBSCAN and related algorithms can be found at https://hdbscan.readthedocs.io/en/latest/basic_hdbscan.html. The main benefit of the hdbscan function is its simplicity. Users are only required to optimise a single parameter; the minimum number of points to comprise a cluster, termed minPts. The optimal value for this parameter will depend upon the structure and resolution of the data, so some initial exploration is required. Given that a subcellular compartment will need to comprise at least 10-15 marker proteins for use in machine learning (discussed later in this workflow), we recommend testing values between 10 and 20 for minPts, as demonstrated below.

## Test a cluster size between 10 and 20 proteins per cluster
min_N <- c(10:20)
cluster_N <- vector(mode = "numeric", length = 10L)

## Run HDBSCAN
for (i in seq_along(min_N)) {
  result <- unstim_msn %>%
    exprs() %>%
    hdbscan(minPts = min_N[i])
  cluster_N[i] <- max(result$cluster)
}

## Structure results into a data.frame
hdb_opt <- data.frame(min_N, cluster_N)

## Plot minimum cluster size against resulting number of clusters
hdb_opt %>%
  ggplot(aes(x = min_N, y = cluster_N)) +
  geom_col() +
  theme_bw() +
  labs(x = "Min proteins per cluster",
       y = "Number of clusters")



**Figure 10.**
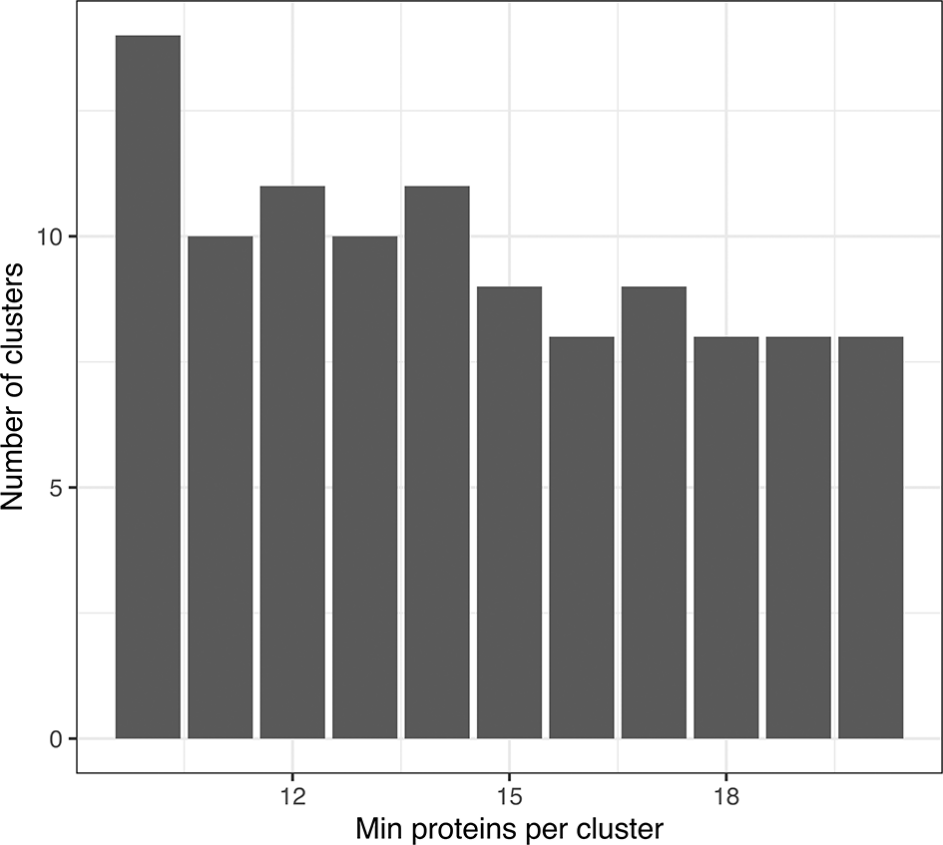
Barplot showing the number of clusters detected when specifying a minimum cluster size on the unstimulated data.


Favourably, the unsupervised algorithm has identified a reasonable number of clusters within our correlation profiling data. Further, as we expect, the total number of discovered clusters decreases as we increase the value of minPts ( Figure 10). Whilst it may be worth exploring the results of clustering with various minPts, we will here demonstrate how to look at the results derived from a minPts value of 10. We first run the HDBSCAN algorithm with our selected minPts and append these results to the fData of our unstim_msn object.

## Run HDBSCAN using optimal minimum cluster size - here 10
hdb_results <- unstim_msn %>%
  exprs() %>%
  hdbscan(minPts = 10)

## Add the results of HDBSCAN to our MSnSet
fData(unstim_msn)$hdb_cluster_id <- hdb_results$cluster
fData(unstim_msn)$hdb_cluster_prob <- hdb_results$membership_prob

## Check how many proteins are in each cluster
unstim_msn %>%
  fData() %>%
  pull(hdb_cluster_id) %>%
  table()


## .
##    0   1   2   3   4   5   6   7    8   9  10   11  12   13   14
## 3496  43  10  31  48  36  15  99  584  10  14  103  14  650  548



As we can see, hdbscan has allocated the proteins to one of the 14 clusters it has found in the data. This information is found in hdb_results$cluster and and we have added it to our MSnSet where it can be accessed via fData(unstim_msn)$hdb_cluster_id . If we check the column names of the fData we can verify the results have been stored in the MSnSet.

## Check columns in fData
unstim_msn %>%
  fvarLabels()


##  [1] "Checked"                     "Tags"
##  [3] "Confidence"                  "Identifying.Node.Type"
##  [5] "PSM.Ambiguity"               "Contaminant"
##  [7] "Number.of.Proteins"          "Master.Protein.Accessions"
##  [9] "Master.Protein.Descriptions" "Protein.Accessions"
## [11] "Protein.Descriptions"        "Delta.Cn"
## [13] "Rank"                        "Search.Engine.Rank"
## [15] "Concatenated.Rank"           "Ions.Matched"
## [17] "Matched.Ions"                "Total.Ions"
## [19] "Activation.Type"             "MS.Order"
## [21] "Quan.Info"                   "Number.of.Protein.Groups"
## [23] "hc"                          "hdb_cluster_id"
## [25] "hdb_cluster_prob"



Notice that some proteins are allocated to cluster “0”. The “0” category denotes proteins which did not fit into one of the 14 discovered clusters.

**Figure 11.**
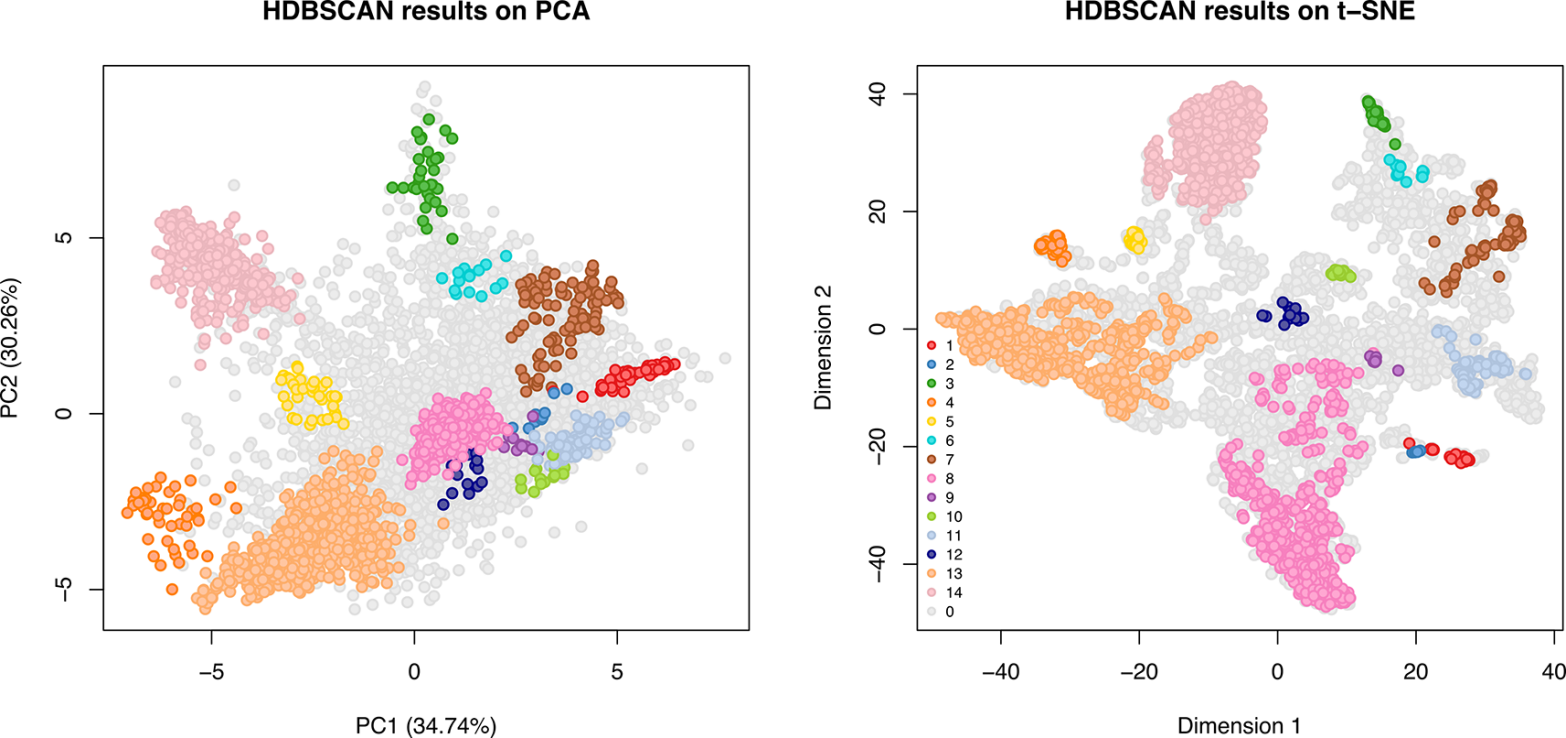
PCA and t-SNE plots of the untimulated data showing the results of unsupervised clustering with HDBSCAN. One point represents one protein and proteins assigned to one of the 14 found HDBSCAN clusters are highlighted by colours. Grey points represent proteins which were not assigned to an HDBSCAN cluster during unsupervised learning.

We can use the plot2D function to generate PCA and t-SNE plots with the discovered clusters annotated ( Figure 11). As before, we pass our unstim_msn object and specify the dimensionality reduction method we wish to use. We also tell the function which column of our fData to be used for colouring the points - here we use the newly created hdb_cluster column. Finally, we pass the argument unknown = "0" so that the large cluster of uncertain proteins remains unlabelled.

par(mfrow = c(1, 2))

## Visualise HDBSCAN unsupervised clustering using PCA
unstim_msn %>%
  plot2D(fcol = "hdb_cluster_id", method = "PCA", unknown = "0",
         main = "HDBSCAN results on PCA")

## Visualise HDBSCAN unsupervised clustering using t-SNE
set.seed(399)
unstim_msn %>%
  plot2D(fcol = "hdb_cluster_id", method = "t-SNE", unknown = "0",
         main = "HDBSCAN results on t-SNE")

## Add legend
unstim_msn %>%
  addLegend(fcol = "hdb_cluster_id", unknown = "0", cex = .7)

**Validation of clusters**: Visualisation confirms that the clustering looks real and sensible. Therefore, we will use gene ontology (GO) enrichment analyses to determine which subcellular niches each of the discovered clusters represent. Many packages exist to facilitate GO enrichment analysis in R, including topGO , ^32^ GOfuncR , ^33^ and clusterProfiler . ^34^ Here we will use the enrichGO function in the clusterProfiler package. The aim of this gene ontology enrichment analysis is to determine whether the proteins assigned to a given cluster are enriched for any cellular component (CC) GO terms. That is, we ask whether the proteins in a cluster are annotated with a CC GO term more than expected by chance based on the entire dataset.

To use the enrichGO function we will pass a vector containing the protein accessions of interest, here those within a given HDBSCAN cluster. We also need to provide a background, or ‘ universe’, which is a vector containing the accessions of all proteins within our spatial map. Other arguments include the keyType to tell the function that we are using UniProt accessions, OrgDb to pass a database object for the species of interest (here org.Hs.eg.db for human samples), and ont to indicate that we wish to consider only GO terms annotated as cellular component (CC). For users studying other model organisms, the org.Hs.eg.db object can be swapped out for a database corresponding to the species of interest e.g., org.Mm.eg.db for mouse. Users mapping the spatial proteome of a non-model organism may need to manually assess the proteins comprising each cluster to determine their identities.

Below we demonstrate how to carry out GO enrichment on cluster number 1.

## Extract protein accessions in the cluster
protein_id <- unstim_msn %>%
  fData() %>%
  filter(hdb_cluster_id == 1) %>%
  pull(Master.Protein.Accessions)

## Extract all protein accessions in the data
background_proteins <- unstim_msn %>%
  fData() %>%
  pull(Master.Protein.Accessions)

## Carry out GO enrichment searching for CC terms
cluster_1_go <- enrichGO(gene = protein_id,
                         universe = background_proteins,
                         OrgDb = org.Hs.eg.db,
                         keyType = "UNIPROT",
                         ont = "CC",
                         pAdjustMethod = "BH",
                         readable = TRUE,
                         pvalueCutoff = 0.01,
                         qvalueCutoff = 0.01)


## View the first few enriched terms
cluster_1_go %>%
  slot("result") %>%
  pull(Description) %>%
  head()


## [1] "proteasome complex"
## [2] "endopeptidase complex"
## [3] "peptidase complex"
## [4] "intracellular protein-containing complex"
## [5] "proteasome regulatory particle"
## [6] "proteasome accessory complex"



We can plot the results using the barplot function. We pass the argument showCategory = 10 to tell enrichGO to only plot the first 10 enriched terms. Other plotting functions are available in the enrichGO package. We refer users to the online book for the package which can be found at https://yulab-smu.top/biomedical-knowledge-mining-book/.

## Plot results
barplot(cluster_1_go, showCategory = 10) +
  ggtitle("Enriched terms in Cluster 1")



**Figure 12.**
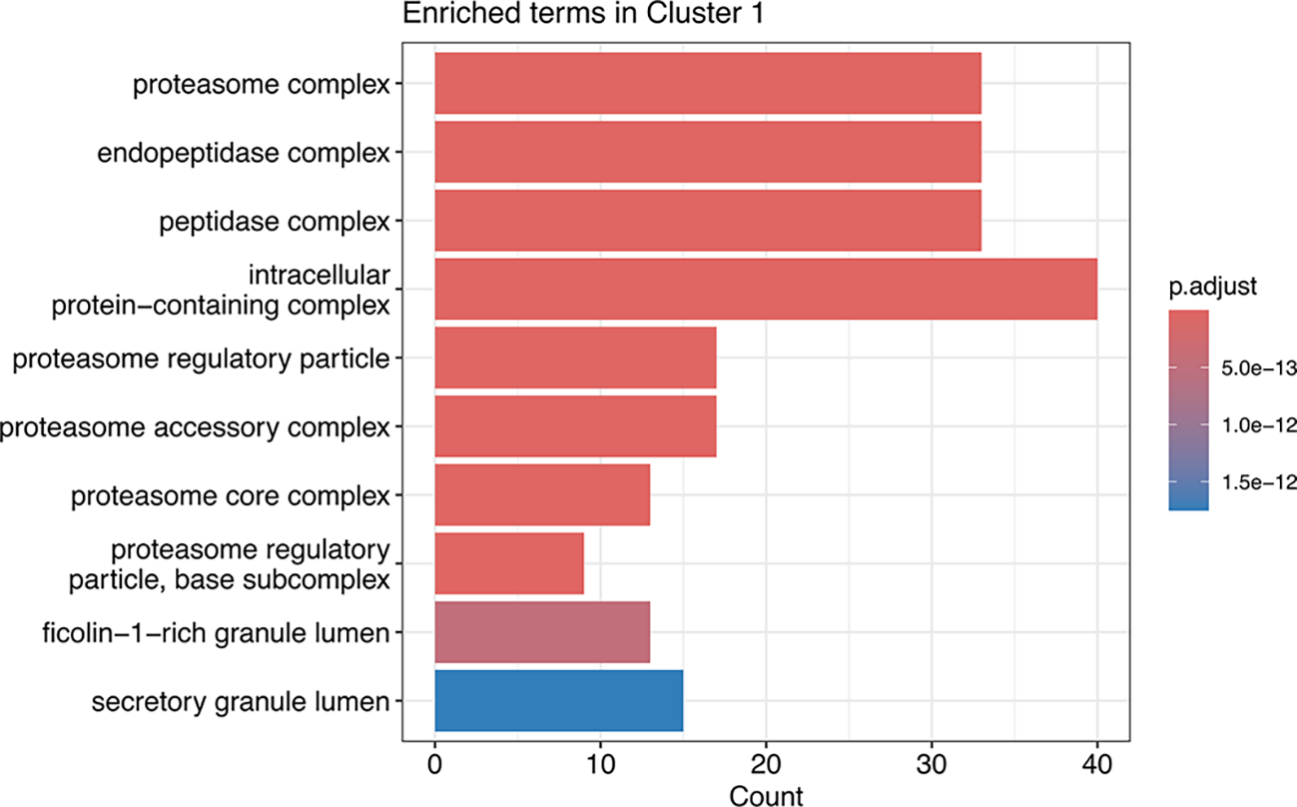
Gene Ontology Cellular Compartment annotation term enrichment for Cluster 1 of the HDBSCAN unsupervised learning results. Bars are coloured according the -log10(adj.p-value) for each term found. The x-axis count refers to the number proteins associated with that term in the Cluster 1.

The results of GO enrichment ( Figure 12) indicate that cluster 1 represents the proteasome complex. This provides confidence that the proteasome is sufficiently resolved in our dataset and should be included as a compartment in the downstream analyses.

To simplify the investigation of all discovered clusters, the code below can be used to generate a list of the enriched CC GO terms for each cluster.

## Extract the number of clusters we have found
n <- hdb_results[["cluster"]] %>%
  max()

## Define protein background
background_proteins <- unstim_msn %>%
  fData() %>%
  pull(Master.Protein.Accessions)

## Initialise a container for the GO output
go_result <- vector("list", n)

## For loop over each cluster number carrying out GO enrichment
for (i in 1:n) {
  ## Step (1) - extract protein IDs in the cluster
  protein_id <- unstim_msn %>%
    fData() %>%
    filter(hdb_cluster_id == i) %>%
    pull(Master.Protein.Accessions)

  ## Step (2) - run enrichGO
  go_result[[i]] <- enrichGO(gene = protein_id,
                        universe = background_proteins,
                        OrgDb = org.Hs.eg.db,
                        keyType = "UNIPROT",
                        ont = "CC",
                        pAdjustMethod = "BH",
                        readable = TRUE)
}



You will notice that in the above code we did not pass thresholds the p-value cutoff i.e., pvalueCutoff and qvalueCutoff as we did in the first example. When no thresholds are passed all GO CC terms found per cluster are recovered whether they are significant to not. This can be useful if you wish to examine different thresholds.

We can filter these results manually using filter, for example, at a given p- and q-value.

## Filter results manually by p- and q-value
enriched_terms <- vector("list", n)
for (i in 1:n) {
  enriched_terms[[i]] <- go_result[[i]] %>%
    slot("result") %>%
    filter(p.adjust < 0.01 & qvalue < 0.01) %>%
    pull(Description)
}

names(enriched_terms) <- paste0("Cluster", 1:n)



### Step 2: Generating an initial marker list

Having visualised the data to validate the presence of clusters and explored which subcellular organelles may be resolved in the dataset, we can now start with an initial marker list. For model organisms, pre-existing marker lists can often be derived from published subcellular proteomics datasets. For simplicity, the pRoloc package stores a number of curated marker sets as well as published marker sets, as discussed below. However, for non-model organisms with limited pre-existing subcellular proteomics data it may be necessary to manually curate an initial marker list. In such cases we would advise users to apply unsupervised clustering to their data in order to guide marker discovery.

**Marker lists from** pRolocmarkers: The pRoloc package contains marker protein lists for a number of organisms, including curated organism reference sets and published marker lists. To see which marker sets are available we can use the pRolocmarkers function. Type ?pRolocmarkers for more details.

## View available marker sets within pRoloc
pRolocmarkers()


## 14 marker lists (version 2) available:
## Arabidopsis thaliana [atha]:
##  Ids: TAIR, 543 markers
## Drosophila melanogaster [dmel]:
##  Ids: Uniprot, 179 markers
## Gallus gallus [ggal]:
##  Ids: IPI, 102 markers
## Homo sapiens [hsap_christopher]:
##  Ids: Uniprot, 1509 markers
## Homo sapiens [hsap_geladaki]:
##  Ids: Uniprot, 579 markers
## Homo sapiens [hsap_itzhak]:
##  Ids: Uniprot, 1076 markers
## Homo sapiens [hsap_villaneuva]:
##  Ids: Uniprot, 682 markers
## Homo sapiens [hsap]:
##  Ids: Uniprot, 872 markers
## Mus musculus [mmus_christoforou]:
##  Ids: Uniprot, 922 markers
## Mus musculus [mmus]:
##  Ids: Uniprot, 937 markers
## Saccharomyces cerevisiae [scer_sgd]:
##  Ids: SGD, 259 markers
## Saccharomyces cerevisiae [scer_uniprot]:
##  Ids: Uniprot, 259 markers
## Toxoplasma gondii [toxo_barylyuk]:
##  Ids: ToxoDB gene identifier, 718 markers
## Trypanosoma brucei [tryp_moloney]:
##  Ids: TriTrypDB gene identifier, 891 markers



We can see a number of lists, each labelled with species, ID type (UniProt protein accessions, species-specific gene IDs etc.) and the number of markers. The name of the marker set is indicated inside of square brackets. Reference marker sets for model organisms are named after their species: atha for the *Arabidopsis thaliana* reference set, dmel for *Drosophila melanogaster*, hsap for *Homo sapiens*, mmus for *Mus musculus*, and ggal for *Gallus gallus.* Marker sets extracted directly from publications are named using the nomenclature species_author e.g., hsap_christopher for the marker list used in Christopher et al. ^13^

To use one of the marker lists stored in pRolocmarkers, users should first extract the marker list into a named character vector by using the pRolocmarkers function and passing the name of the desired marker list.

## Example extract markers from pRolocmarkers
hsap_christopher <- pRolocmarkers("hsap_christopher")



These markers can then be used to annotate the data via the addMarkers function, as demonstrated below.

**Adding user-defined marker lists:** It is possible to import and apply a user-defined marker list. The markers should be imported as either (i) a named vector where subcellular marker annotations are entries and feature accessions are the corresponding names, or (ii) from a .csv file with two columns, one for the subcellular marker annotation and another for the corresponding feature name. Of note, the feature names used in the marker list must be of the same format as those in the featureNames of the MSnSet to be annotated. In the use-case the format is UniProt protein accessions but featureNames could also be gene names or organisms-specific identifiers. We show how to load from a .csv file in the below code chunk and add annotation to the proteins in the data based on the marker list we have provided. A new column will be added to the data, which we will call “markers_initial”. This is done by passing the argument mfcol = "markers_initial". For users who have extracted markers from pRolocmarkers, the code below should be edited to pass the extracted named character object (e.g., hsap_christopher generated above) to the markers argument.

## Add markers to the MSnSet
unstim_msn <- addMarkers(object = unstim_msn,
                         markers = "mrk.csv",
                         mcol = "markers_initial")


## Markers in data: 1502 out of 5701

## organelleMarkers
##         chromatin            cytosol                ER               ERGIC
##                41                370               274                  15
##                GA           lysosome     mitochondrion             nucleus
##                24                 33               363                 224
##        peroxisome                 PM        proteasome  ribosome/complexes
##                17                 44                33                  64
##           unknown
##              4199



Upon successfully annotating the markers, a table is printed to the screen summarising the number of markers that have been added to the data. We see a new column has been added to the fData of our MSnSet to annotate these proteins in our dataset, called “markers_initial”.

## Print the column names of the fData
unstim_msn %>%
  fvarLabels()


##  [1] "Checked"                     "Tags"
##  [3] "Confidence"                  "Identifying.Node.Type"
##  [5] "PSM.Ambiguity"               "Contaminant"
##  [7] "Number.of.Proteins"          "Master.Protein.Accessions"
##  [9] "Master.Protein.Descriptions" "Protein.Accessions"
## [11] "Protein.Descriptions"        "Delta.Cn"
## [13] "Rank"                        "Search.Engine.Rank"
## [15] "Concatenated.Rank"           "Ions.Matched"
## [17] "Matched.Ions"                "Total.Ions"
## [19] "Activation.Type"             "MS.Order"
## [21] "Quan.Info"                   "Number.of.Protein.Groups"
## [23] "hc"                          "hdb_cluster_id"
## [25] "hdb_cluster_prob"            "markers_initial"



If you wish to remove the markers from your data you can assign NULL to the output i.e., one would type fData(unstim_msn)[, "markers_initial"] <- NULL. Additionally, if you wish to use a different name to “markers_initial”, to refer to your annotation list users can pass a new name using the argument mcol when using addMarkers. See the help documentation of addMarkers by typing ?addMarkers for more details.

**An additional note on minimum number of markers per compartment:** Determining the optimal number of markers to include in the training data can be challenging. The inclusion of more training data (markers) is usually thought to increase the generalisability of a model, although this is only true if these markers represent the full diversity of data and do not lead to model over-fitting. While there are no fixed rules on the minimum number of training examples needed to learn a classifier, we suggest (1) having a sufficient number of markers per class (compartment) to run cross-validation e.g. 10-15 for a 5-fold cross validation, (2) more markers than quantitation features e.g. for correlation profiling experiment with 10 abundance channels, > 10 markers per compartment. Some classifiers also require the number of examples per class to be balanced. Unfortunately, the number of proteins known to reside in a subcellular niche can vary greatly, and some niches simply have too few proteins to add more markers. For example, the peroxisome is a relatively small organelle with few available marker proteins. This means that for experiments with many fractions and multiple replicates it may not be possible to have the number of peroxisomal markers exceed the number of quantitative channels. Nevertheless, users should be aware of these general guidelines and try to meet them where possible.

### Step 3: Evaluating markers

After annotating the markers we again use dimensionality reduction within the plot2D function as a visualisation tool. This time we pass fcol = "markers_initial" to colour our proteins based on the markers_initial column within our fData. As outlined above, we expect markers to cluster together on these 2D plots.

par(mfrow = c(1, 3))

unstim_msn %>%
plot2D(method = "PCA", fcol = "markers_initial",
       main = "Initial marker resolution PCA")

set.seed(399)
unstim_msn %>%
plot2D(method = "t-SNE", fcol = "markers_initial",
       main = "Initial marker resolution t-SNE")

unstim_msn %>%
  addLegend(where = "bottomleft", fcol = "markers_initial", cex = .7)



**Figure 13.**
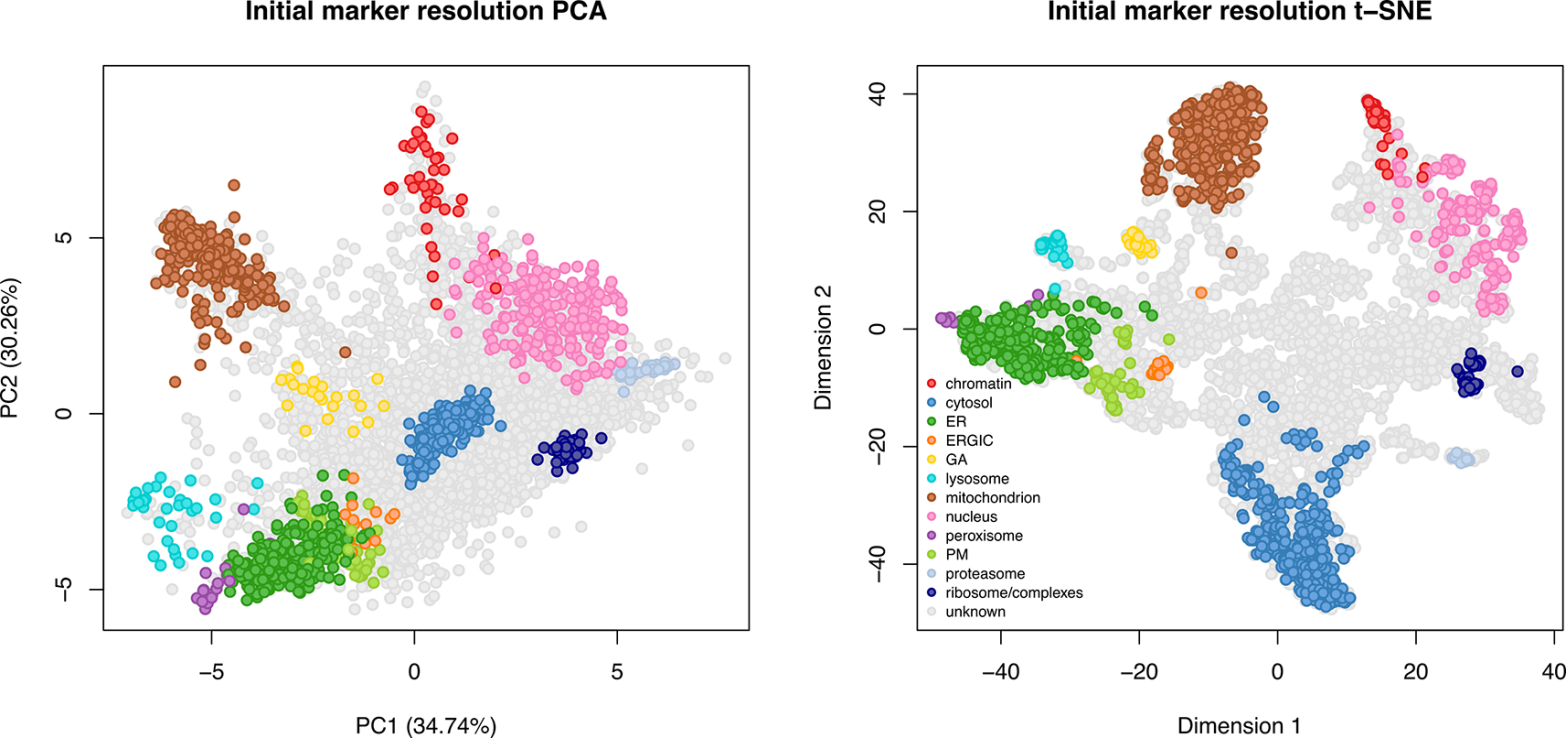
PCA and t-SNE plots of the unstimulated data annotated with subcellular markers. Proteins known to belong to a subcellular niche (markers) are highlighted by colours. Grey points denote unlabelled (unknown) proteins.



**Figure 14.**
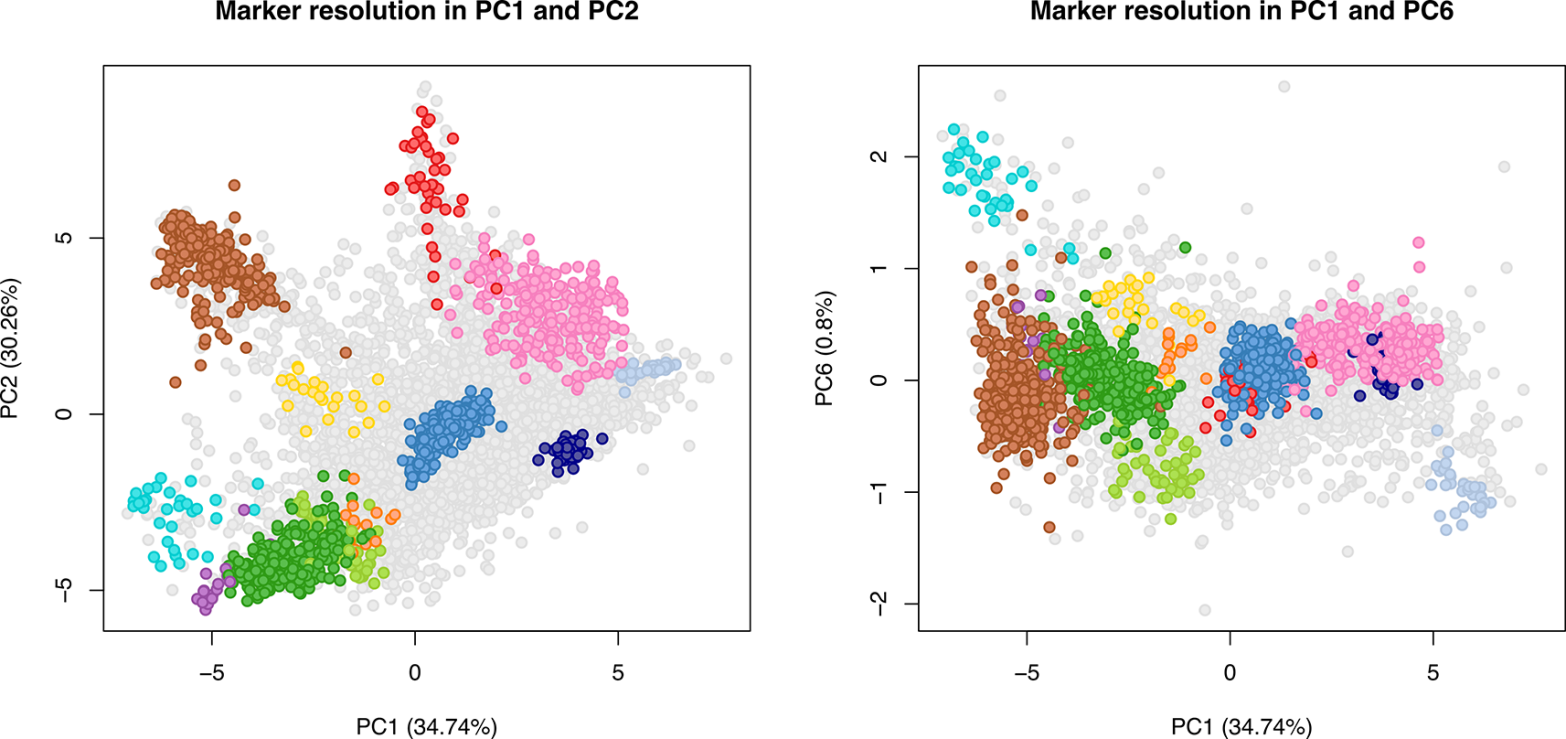
PCA plots of the unstimulated data annotated with subcellular markers. PC dimension 1 versus PC dimension 1 (left), and PC dimension 1 versus PC dimension 6 (right) is shown. Proteins known to belong to a subcellular niche (markers) are highlighted by colours. Grey points denote unlabelled (unknown) proteins.

As expected, the majority of markers cluster together with other proteins from the same subcellular compartment ( Figure 13). However, we can see some protein markers which do not cluster as tightly with the rest of their compartment. Additionally, whilst the t-SNE plot shows all clusters to be relatively well separated, the PCA plot displays an overlap between the ER (dark green) and plasma membrane (light green). This is because the plot2D function uses PC1 and PC2 by default which limits visualisation to compartments resolved in these first two dimensions. We can pass a vector of alternative PC dimensions to the dims argument within plot2D to explore resolution in these lower PCs. Below we show that the ER and plasma membrane are better differentiated when visualising PC1 and PC6 ( Figure 14).

par(mfrow = c(1, 2))

## Visualise PC1 and PC2 - the PCs which explain the most variance
unstim_msn %>%
plot2D(method = "PCA", fcol = "markers_initial",
       main = "Marker resolution in PC1 and PC2")

## Visualise PC1 and PC6 - explain less variance but differentiate some clusters
unstim_msn %>%
plot2D(method = "PCA", fcol = "markers_initial",
       dims = c(1, 6), main = "Marker resolution in PC1 and PC6")



Importantly, although dimensionality reduction provides a convenient visualisation tool, neither the PCA nor the t-SNE co-ordinates represent the data input for downstream supervised machine learning classification algorithms. The training data for classification are the marker protein correlation profiles. Therefore, it is critical to look directly at these profiles as well as the 2D maps.

**Figure 15.**
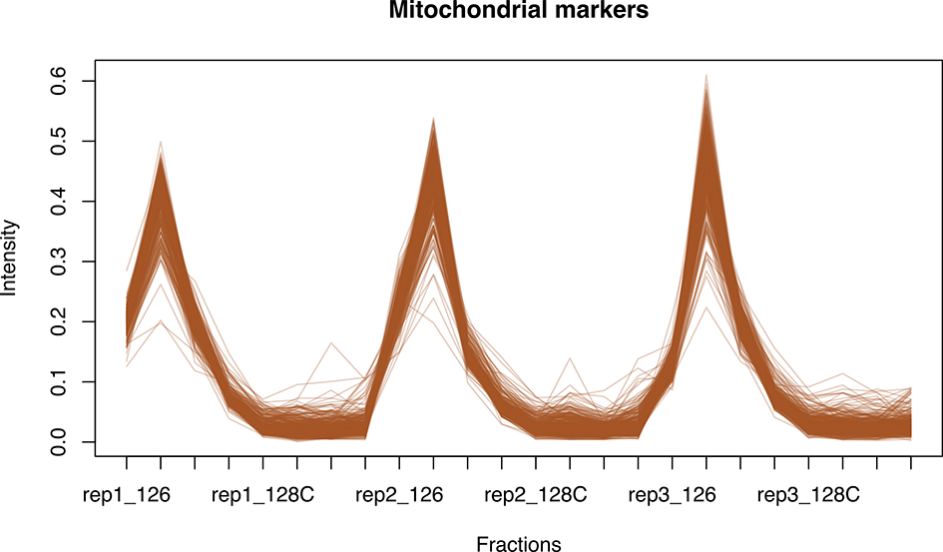
Protein profile distribution plot of the mitochondrial marker proteins.

The plotDist function within pRoloc generates a line plot showing the protein distribution profiles ( Figure 15). We pass our unstim_msn object and subset the proteins annotated as “mitochondrion” in the markers_initial column of the fData.

## Extract indices for mitochondrion markers
mt <- fData(unstim_msn)$markers_initial == "mitochondrion"

## Plot the distributions of mitochondrial markers
plotDist(object = unstim_msn[mt, ], pcol = "#A65628", las = 2)
title("Mitochondrial markers")



A for loop can be used to plot the distribution of each marker class, as outlined in the code chunk below. The getMarkerClasses function can be used to return a vector of all subcellular marker compartments in a given MSnSet.

## Define marker compartments
marker_organelles <- getMarkerClasses(unstim_msn, fcol = "markers_initial")

## Define colours
my_cols <- getStockcol()

## Plot marker distribution profile per compartment
par(mfrow = c(4, 3))
for (i in 1:length(marker_organelles)) {
  marker_indices <- fData(unstim_msn)$markers_initial == marker_organelles[i]
  plotDist(unstim_msn[marker_indices, ],
           pcol = my_cols[i],
           xlab = "Fraction",
           ylab = "Intensity")
  title(marker_organelles[i])
}



**Figure 16.**
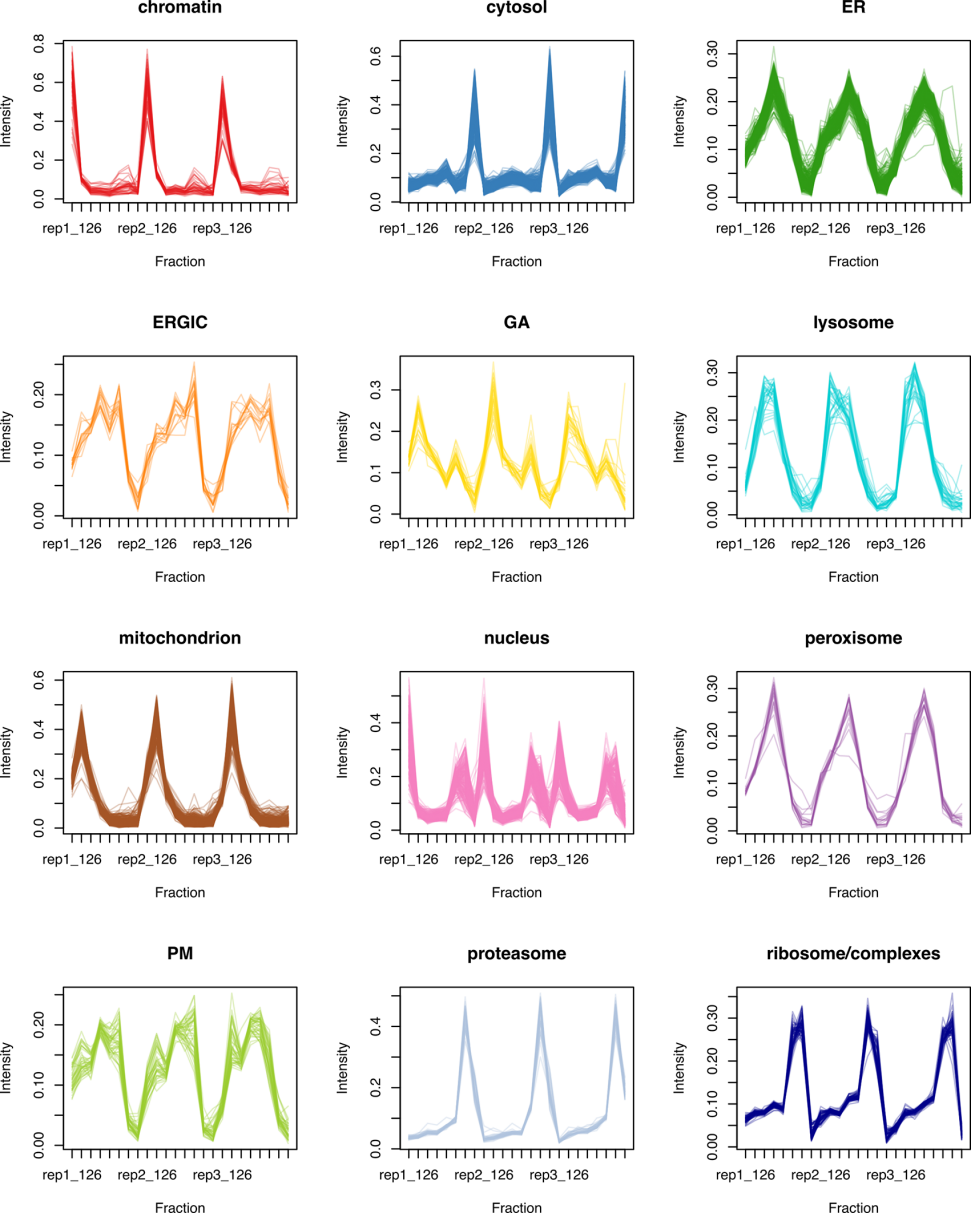
Protein correlation profiles for each set of marker proteins, across all replicates in the unstimulated dataset.

Plotting the concatenated profiles ( Figure 16) is useful as it allows us to assess the suitability of markers across all three replicates at once. Importantly, a protein can only be a marker if it behaves as such across all replicates. Overall, this marker set is a good fit to the data. Nevertheless, there are a few markers that could be removed. For example, the third plot shows that one of the ER markers does not behave well in replicate 3 and, therefore, perhaps is not the best candidate as input for machine learning. Similarly, we can see a few outliers across the golgi apparatus (GA), lysosome, mitochondrion and peroxisome.

**An additional note on outlier marker removal:** Decisions regarding what constitutes an “outlier marker” can be challenging. In most correlation profiling experiments the marker profiles will contain a certain degree of noise, and this is to be expected. Further, the degree of noise will differ across subcellular compartments depending upon how well they behave during the biochemical fractionation process. For instance, in the use-case data the proteasomal markers have a very tight profile with minimal noise whilst the nuclear markers have a wider profile indicating more variation in the behaviour of these proteins during fractionation. Whilst it would be possible to extract a subset of nuclear markers to generate a much tighter set of profiles (which would translate to a tighter cluster when visualised via dimensionality reduction), this would be an inaccurate representation of the data. Indeed, over-curation of markers to generate unrealistically tight clusters can lead to over-fitting of downstream protein localisation classification models. In other words, the nuclear markers would no longer be a true representation of the nucleus and the model may fail to recognise which of our unknown proteins should be classified as nuclear. The goal of marker curation is to select a set of markers which accurately represent the dataset being analysed. Moreover, some machine learning models are more robust to outliers than others.

**Interactive data visualisation:** Visual exploration of correlation profiling datasets is a key aspect of the data analysis. As outlined above, the visualisation of marker protein distribution profiles as well as the annotation of these proteins on 2D maps facilitates the marker curation process required prior to protein localisation classification. To make this process of visual data exploration easier and reduce the need for excess plotting, the pRolocVis function from the pRolocGUI package can be used. We simply pass our MSnSet object to the pRolocVis function, specify a method of dimensionality reduction and a column from our fData which we wish to visualise, here the markers_initial column.

## Open pRolocGUI for unstimulated dataset
pRolocVis(object = unstim_msn,
          method = "PCA",
          fcol = "markers_initial")



**Figure 17.**
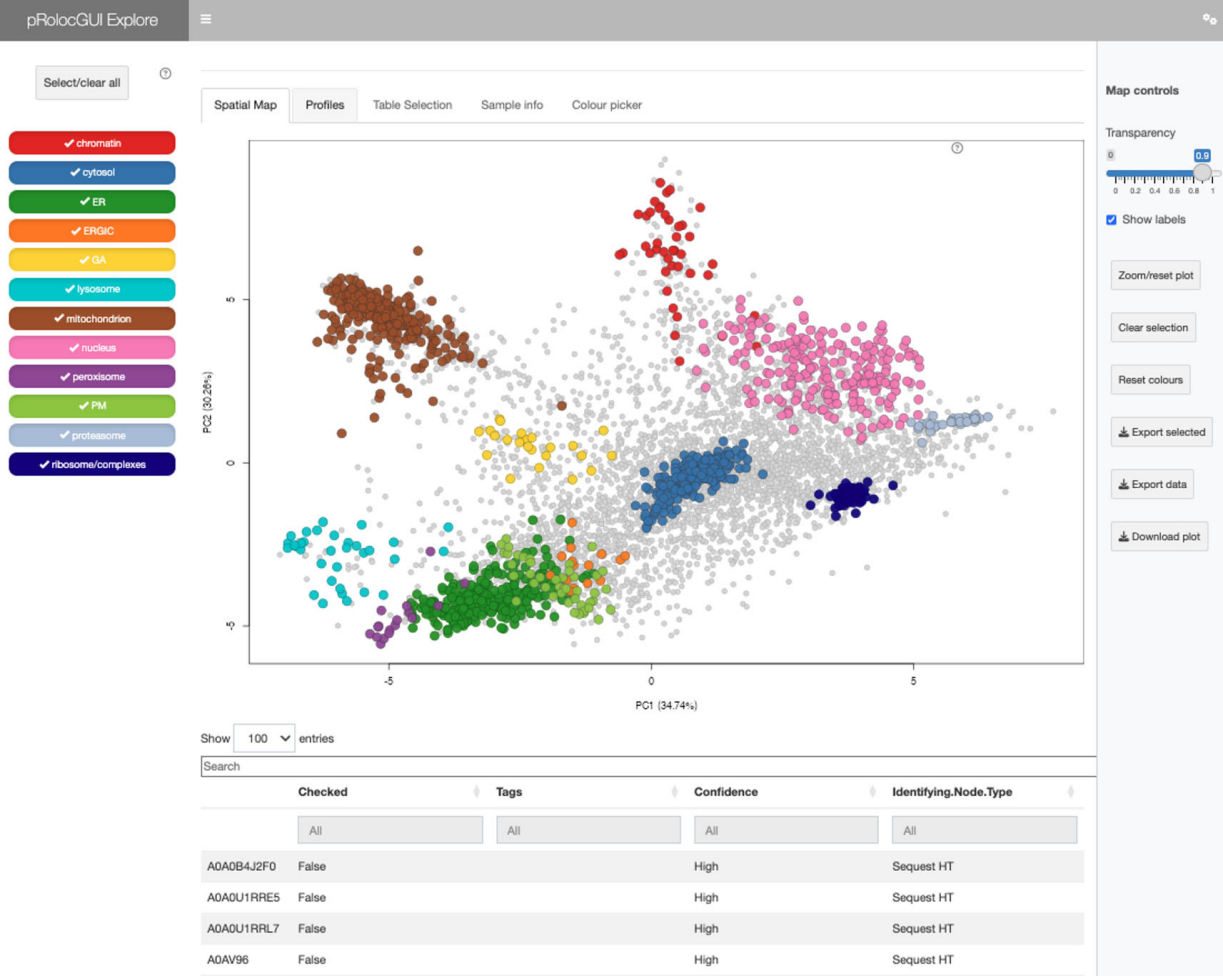
Screenshot of the pRolocVis GUI application.

Using this interactive visualisation tool ( Figure 17) enables us to easily identify which proteins the outlier points and profiles correspond to.

**Quantifying resolution using** QSep: In addition to identifying outlying markers in each of the annotated compartments, users should also consider whether all of the initial compartments have sufficient resolution to be included in downstream classification. From the marker plots generated above, the initial compartments in our marker set do seem to be reasonably well resolved. However, we can take a more quantitative approach to exploring resolution by using the QSep function within pRoloc. QSep is a method for quantifying the separation of clusters in subcellular spatial proteomics datasets. ^29^

## Carry out QSep
qsep_res <- QSep(object = unstim_msn, fcol = "markers_initial")

## Verify
qsep_res


## Object of class 'QSep'.
## Data: unstim_msn
## With 12 sub-cellular clusters.



The QSep function returns a specialised object of class QSep. Within this object we can access matrices containing two types of QSep score: (1) the original and (2) normalised QSep scores. Original QSep scores are calculated as the average Euclidean distance between two clusters and thus directly quantify how far apart the clusters are when visualised via PCA. These scores are calculated using one of the clusters as a reference cluster. Since the distance between two clusters is the same regardless of which is the reference, the original QSep scores will be a symmetrical matrix. We can access this matrix by executing qsep_res@x.

## Look at original QSep scores
qsep_res@x


##                      cytosol        ER mitochondrion chromatin peroxisome
## cytosol            0.1800847 0.7565219     0.9969295 1.1244650 0.80235075
## ER                 0.7565219 0.1154529     0.7017802 0.9497942 0.17624056
## mitochondrion      0.9969295 0.7017802     0.1436030 0.8841179 0.71492610
## chromatin          1.1244650 0.9497942     0.8841179 0.2616475 0.99379818
## peroxisome         0.8023508 0.1762406     0.7149261 0.9937982 0.09772863
## proteasome         0.7640793 0.8799295     1.1106904 1.1806420 0.94655883
## nucleus            0.8624014 0.6660389     0.8338416 0.5975924 0.76076215
## GA                 0.7640110 0.3628224     0.4624246 0.8593753 0.43007672
## lysosome           0.8243242 0.2863528     0.5884373 1.0261414 0.24559925
## PM                 0.7667704 0.1806928     0.6962547 0.9294028 0.29016134
## ERGIC              0.7566707 0.2031881     0.7126456 0.9431341 0.32235418
## ribosome/complexes 0.8244196 0.5756393     0.9230781 1.0278682 0.68518018
##                    proteasome   nucleus        GA  lysosome        PM
## cytosol             0.7640793 0.8624014 0.7640110 0.8243242 0.7667704
## ER                  0.8799295 0.6660389 0.3628224 0.2863528 0.1806928
## mitochondrion       1.1106904 0.8338416 0.4624246 0.5884373 0.6962547
## chromatin           1.1806420 0.5975924 0.8593753 1.0261414 0.9294028
## peroxisome          0.9465588 0.7607622 0.4300767 0.2455992 0.2901613
## proteasome          0.1181278 0.7479004 0.8270153 0.9653016 0.8682598
## nucleus             0.7479004 0.2522033 0.5780509 0.8035946 0.6095942
## GA                  0.8270153 0.5780509 0.1522524 0.3668160 0.3340057
## lysosome            0.9653016 0.8035946 0.3668160 0.1351328 0.3686466
## PM                  0.8682598 0.6095942 0.3340057 0.3686466 0.1116434
## ERGIC               0.8166343 0.5800091 0.3147845 0.3788261 0.1421239
## ribosome/complexes  0.5307091 0.5016937 0.5379438 0.7190812 0.5111228
##                         ERGIC ribosome/complexes
## cytosol            0.75667068          0.8244196
## ER                 0.20318805          0.5756393
## mitochondrion      0.71264563          0.9230781
## chromatin          0.94313409          1.0278682
## peroxisome         0.32235418          0.6851802
## proteasome         0.81663434          0.5307091
## nucleus            0.58000907          0.5016937
## GA                 0.31478445          0.5379438
## lysosome           0.37882611          0.7190812
## PM                 0.14212393          0.5111228
## ERGIC              0.09899275          0.4394807
## ribosome/complexes 0.43948066          0.0809796



By contrast to these raw scores, normalised QSep scores are calculated as the ratio of average Euclidean distance between clusters to the average distance within the reference cluster. This means that the normalised QSep scores represent not only the distance between clusters but this distance relative to how tight the reference cluster is. As a result, we have an asymmetrical matrix where the score is different when we use each of the two compared clusters as the reference. This matrix is accessed using qsep_res@xnorm, as shown below.

## Look at the normalised QSep scores
qsep_res@xnorm


##                      cytosol       ER mitochondrion chromatin peroxisome
## cytosol             1.000000 4.200923      5.535893  6.244090   4.455408
## ER                  6.552643 1.000000      6.078495  8.226679   1.526514
## mitochondrion       6.942260 4.886946      1.000000  6.156681   4.978489
## chromatin           4.297633 3.630053      3.379042  1.000000   3.798233
## peroxisome          8.209987 1.803367      7.315421 10.168956   1.000000
## proteasome          6.468244 7.448963      9.402449  9.994618   8.013008
## nucleus             3.419469 2.640881      3.306228  2.369487   3.016464
## GA                  5.018056 2.383033      3.037224  5.644413   2.824762
## lysosome            6.100105 2.119047      4.354512  7.593578   1.817466
## PM                  6.868035 1.618482      6.236419  8.324748   2.599002
## ERGIC               7.643698 2.052555      7.198968  9.527305   3.256341
## ribosome/complexes 10.180584 7.108448     11.398897 12.692928   8.461146
##                    proteasome  nucleus       GA lysosome       PM    ERGIC
## cytosol              4.242889 4.788866 4.242510 4.577425 4.257833 4.201749
## ER                   7.621542 5.768921 3.142600 2.480255 1.565078 1.759921
## mitochondrion        7.734450 5.806574 3.220160 4.097667 4.848468 4.962609
## chromatin            4.512338 2.283960 3.284477 3.921847 3.552118 3.604598
## peroxisome           9.685584 7.784435 4.400724 2.513074 2.969052 3.298462
## proteasome           1.000000 6.331283 7.001023 8.171673 7.350175 6.913144
## nucleus              2.965466 1.000000 2.292004 3.186297 2.417075 2.299768
## GA                   5.431871 3.796663 1.000000 2.409263 2.193764 2.067517
## lysosome             7.143355 5.946703 2.714485 1.000000 2.728032 2.803362
## PM                   7.777084 5.460193 2.991721 3.302002 1.000000 1.273017
## ERGIC                8.249436 5.859107 3.179874 3.826807 1.435700 1.000000
## ribosome/complexes 6.553614 6.195310 6.642954 8.879782 6.311747 5.427054
##                  ribosome/complexes
## cytosol                    4.577955
## ER                         4.985921
## mitochondrion              6.427985
## chromatin                  3.928447
## peroxisome                 7.011049
## proteasome                 4.492670
## nucleus                    1.989243
## GA                         3.533237
## lysosome                   5.321293
## PM                         4.578175
## ERGIC                      4.439524
## ribosome/complexes         1.000000



For both raw and normalised QSep scores, a higher score indicates better resolution between clusters.

We can also visualise the results of QSep in multiple ways. Firstly, the plot function within the pRoloc package can be used to generate a boxplot of the QSep scores for each included compartment. The norm argument takes a logical input to indicate whether to use normalised or raw QSep scores.

## Plot a boxplot of raw QSep scores
plot(x = qsep_res, norm = FALSE)

## Plot a boxplot of normalised QSep scores
plot(x = qsep_res, norm = TRUE)



**Figure 18.**
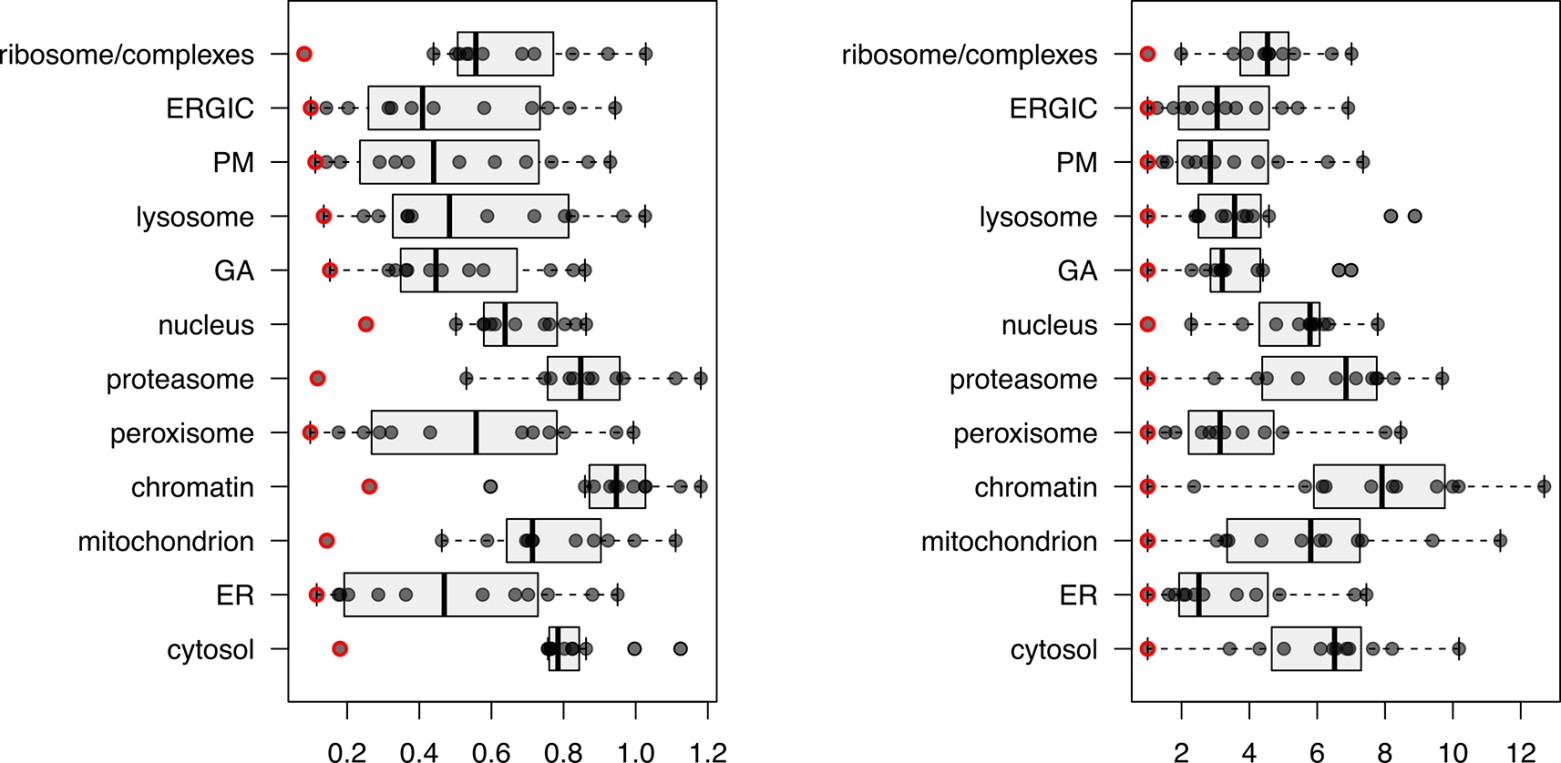
Boxplots displaying the raw (left) and normalised (right) between cluster QSep scores, output from running a QSep analysis on the marker protein correlation profiles of the unstimulated data. The within cluster distances are shown in red.

From these boxplots ( Figure 18) we can see which of our subcellular compartments have higher overall normalised QSep scores, thus indicating that they are well resolved. In the use-case data, ribosome/complexes, chromatin and proteasome are our three most well-resolved compartments.

Secondly, to take a closer look at the pairwise QSep scores we can use the levelPlot function, again with the norm argument to specify which type of score we are interested in.

## Plot a level plot of raw QSep values between each compartment
levelPlot(object = qsep_res, norm = FALSE)

## Plot a level plot of normalised QSep values between each compartment
levelPlot(object = qsep_res, norm = TRUE)



**Figure 19.**
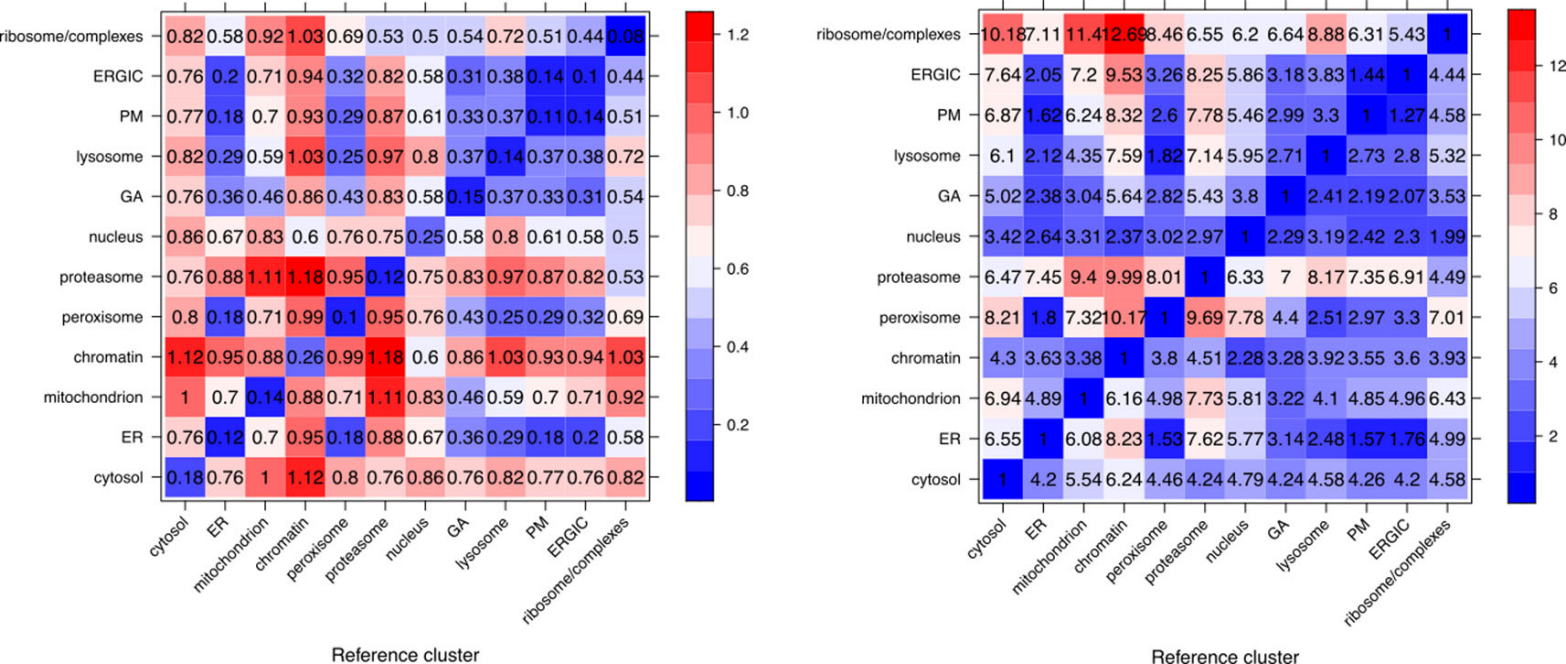
Heatmaps displaying the raw (left) and normalised (right) within (along the diagnol) and between euclidean distances. The colour key differentiates between small distances (blue) and large distances (red).

Looking at the pairwise QSep scores is particularly useful in identifying which subcellular compartments are the least resolved from each other. In the use-case data the plasma membrane (PM) and Endoplasmic-reticulum-Golgi intermediate compartment (ERGIC) have the lowest pairwise resolution ( Figure 19). When resolution between two subcellular compartments is low (i.e., their pairwise QSep scores are low) there is a higher chance of proteins being misclassified between these compartments during downstream machine learning. Indeed, a subcellular compartment could have a high average QSep score, but the ability to correctly classify proteins to that compartment is determined almost entirely by its lowest pairwise QSep score. For compartments where all calculated QSep scores are low, users may consider whether it is appropriate to include the compartment at all. By contrast, for compartments with high overall scores but one or two low pairwise QSep scores it may be worth combining the compartments with lower resolution. After making any changes, QSep scores can be recalculated on the updated annotated MSnSet to determine whether resolution has been improved.

QSep scores are a quantitative measure of cluster (subcellular compartment) separation. Higher QSep scores indicate that clusters are more well resolved from each other. However, users should not aim for unreasonably high QSep scores. As discussed above in “An additional note on outlier marker removal”, over-curation can generate unrealistically tight protein clusters which are no longer representative of the dataset. These clusters would have relatively higher QSep scores, since they are tighter and further separated from other compartments but could still lead to over-fitting during protein localisation classification.

### Step 4: Refining marker lists

Having made an initial assessment of marker suitability, we can now update our marker list accordingly. There are three different aspects to consider here:

1. Removal of outlier markers
2. Combining compartments which lack sufficient resolution
3. Expansion or removal of marker compartments which have an insufficient number of proteins to use as training data for classification

With respect to removing the annotation for individual outlier proteins, we previously visualised the correlation profiles of each marker compartment and used pRolocGUI to interactively identify which proteins the outlier profiles belong to. As we wish to keep track of marker annotation throughout our analysis we create a new column for our final marker list that we will use for analysis. We will call this “markers”. This is the default value of the fcol argument in pRoloc for many of pRoloc functions. This means we can omit fcol in many arguments.

Let’s first create this new list,

fData(unstim_msn)$markers <- fData(unstim_msn)$markers_initial



Check it’s been created,

unstim_msn %>% fvarLabels()


##  [1] "Checked"                     "Tags"
##  [3] "Confidence"                  "Identifying.Node.Type"
##  [5] "PSM.Ambiguity"               "Contaminant"
##  [7] "Number.of.Proteins"          "Master.Protein.Accessions"
##  [9] "Master.Protein.Descriptions" "Protein.Accessions"
## [11] "Protein.Descriptions"        "Delta.Cn"
## [13] "Rank"                        "Search.Engine.Rank"
## [15] "Concatenated.Rank"           "Ions.Matched"
## [17] "Matched.Ions"                "Total.Ions"
## [19] "Activation.Type"             "MS.Order"
## [21] "Quan.Info"                   "Number.of.Protein.Groups"
## [23] "hc"                          "hdb_cluster_id"
## [25] "hdb_cluster_prob"            "markers_initial"
## [27] "markers"



We can now remove the annotation for the following outlying proteins in our new markers column,

## Define marker accessions identified as outliers
outlier_acc <- c("Q9BSE5", "O75063", "O14832", "Q9BTZ2", "Q86T03", "Q9BUM1")

## Convert 'marker' column to unknown
to_rm <- which(fData(unstim_msn)$Master.Protein.Accessions %in% outlier_acc)
fData(unstim_msn)$markers[to_rm] <- "unknown"



We can validate the removal of these 6 proteins by looking at a table of the markers using the getMarkers function. We see that previously had 4199 “unknown” (unannotated) proteins and now have 4205.

getMarkers(unstim_msn, fcol = "markers_initial")


## organelleMarkers
##          chromatin           cytosol               ER              ERGIC
##                 41               370              274                 15
##                 GA          lysosome    mitochondrion            nucleus
##                 24                33              363                224
##         peroxisome                PM       proteasome ribosome/complexes
##                 17                44               33                 64
##            unknown
##               4199


getMarkers(unstim_msn, fcol = "markers")


## organelleMarkers
##          chromatin           cytosol               ER              ERGIC
##                 41               370              273                 15
##                 GA          lysosome    mitochondrion            nucleus
##                 23                32              362                224
##         peroxisome                PM       proteasome ribosome/complexes
##                 15                44               33                 64
##            unknown
##               4205



Next, we consider whether to remove any compartment annotation and leave these proteins as “unknown” or “unlabelled”. Visualisation of PCA and t-SNE plots indicated that all of the annotated marker clusters were reasonably well resolved. Therefore, we will not yet remove or combine any of our clusters. However, when quantifying the resolution of our data using QSep we identified the pairs of clusters with the lowest separation - the PM and ERGIC, PM and ER and ER and ERGIC. Therefore, we can re-assess the inclusion of these compartments based on the results of an initial attempt at protein localisation.

Should users wish to remove the annotation for an entire compartment, the fDataToUnknown function provides an easy way to achieve this. We would pass our MSnSet, the "markers" column and then use the from and to arguments to change our annotations from a given organelle to "unknown" (the default), or another annotation if desired, as shown below.

## Create a test copy of the data
test_msn <- unstim_msn

## Change lysosome to "unknown" in the column "markers"
test_msn <- fDataToUnknown(object = test_msn, fcol = "markers",
                           from = "lysosome", to = "unknown")

## We see the lysosome annotation has been removed and relabelled as "unknown"
getMarkers(test_msn)


## organelleMarkers
##          chromatin           cytosol                 ER            ERGIC
##                 41               370                273               15
##                 GA     mitochondrion            nucleus       peroxisome
##                 23               362                224               15
##                 PM        proteasome ribosome/complexes          unknown
##                 44                33                 64             4237



Similarly, this function could also be used to change the names of compartments, replacing “unknown” in the above code chunk to the desired compartment name.

Finally, if users find that their initial or curated marker lists include compartments of interest which appear to be sufficiently resolved but have too few marker proteins (see the following note “An additional note on minimum number of markers”) it may be necessary to find some additional marker proteins within the dataset. This could be informed by the results of unsupervised clustering, as demonstrated above. Alternatively, users could consider looking at different marker sets to see whether there are any other well-established marker proteins for their compartment of interest.

After updating the marker list, users are advised to re-plot the marker profiles and annotated dimensionality reduction plots to display the final markers. This can be achieved using the same code as above (switching "markers_initial" for "markers"), so for the simplicity of this workflow we will not repeat the plotting here.

### Protein localisation prediction via supervised machine learning

Now that we are confident that we have annotated our dataset with markers that are reliable and representative of our data structure we can use these markers to localise query proteins via supervised machine learning. Whilst unsupervised methods such as HDBSCAN (as used above) can discover tight clusters of proteins with similar behaviours, these algorithms tend to struggle when it comes to identifying compartment borders. There are several supervised and semi-supervised machine learning algorithms available within the pRoloc infrastructure, and these differ with respect to their data output, interpretability, and computational demand. Ultimately, the choice of algorithm is up to the user, and we would recommend trying multiple models to determine which works best on a given set of data.

Within the pRoloc package there is a dedicated vignette to the “Machine learning techniques available in pRoloc”. We also have previously demonstrated how to perform protein localisation prediction in pRoloc using (1) classical machine learing methods in Breckels et al. ^11^ and (2) Bayesian machine learning methods in Crook et al. ^12^ Whilst these two resources are readily available and show applications to correlation profiling data, one of the aims of this article is to provide users with a complete end-to-end workflow for analysing spatial proteomics data. Therefore, we include below how to apply a classical Support Vector Machine (SVM) learning approach before demonstrating two more recent Bayesian algorithms based on t-augmented Gaussian models (TAGM). Whilst the application and interpretation of the latter Bayesian approaches will be shown, details of the algorithms themselves are not explicitly discussed. For more information about the TAGM classifiers users are directed to. ^12, 35^

### Classification using a Support Vector Machine

Support Vector Machines (SVMs) have been widely used for protein subcellular localisation prediction. ^2, 3, 4, 9, 36, 37^ In simple terms, an SVM attempts to find the optimal set of lines or hyperplanes which maximise the distance between classes (here subcellular compartments) in a multi-dimensional space. Once optimised, these hyperplanes are used for classification. For more details about SVM algorithms readers are directed to. ^38^

**Optimising SVM parameters:** As is the case with most supervised learning models, before we can perform any classification the model parameters need to be optimised. We adopt a classical view of training the model parameters and split the data into training and testing sets. By default, in pRoloc all methods allocate 80% of the labelled data (marker proteins) for training and optimisation via cross-validation of the free parameters, namely sigma and cost. The remaining 20% labelled data acts as the test set to which the optimised parameters are applied in order to calculate F1 scores. Briefly, an F1 score can be used as a measure of model accuracy as it combines measurements of both precision and recall. A higher F1 score indicates a better-quality classifier.

In pRoloc the SVM optimisation process is facilitated by the svmOptimisation function. We pass our MSnSet object (here, unstim_msn) to the svmOptimisation function and tell it the name of the fData column where our markers are held (here "markers"). We use the times argument to specify how many iterations of cross-validation to complete and the xval argument to set the value of *n* in n-fold cross validation. Here, we will use the default 5-fold cross-validation ( xval = 5) with 10 iterations ( times = 10). We typically would set times = 100 but for demonstration and computational time we use 10. A local machine with 12 cores available can take up to approximately 60 minutes to run 100 iterations on this specific use-case. Since our training data contains asymmetric class sizes (subcellular compartments with variable numbers of marker proteins), we also set class weights using the class.weights argument to avoid biasing our localisation towards compartments with a greater number of marker proteins. ^39^ Hence, we weight each subcellular compartment by the inverse of the number of marker proteins it has. Finally, to ensure reproducibility of our results we also choose to set a seed.

## Get markers
marker_tbl <- unstim_msn %>%
  getMarkers() %>%
  table()


## organelleMarkers
##          chromatin           cytosol               ER              ERGIC
##                 41               370              273                 15
##                 GA          lysosome    mitochondrion            nucleus
##                 23                32              362                224
##         peroxisome                PM       proteasome ribosome/complexes
##                 15                44               33                 64
##            unknown
##               4205


## Set class weights as inverse of class frequencies
weights <- 1 / marker_tbl[names(marker_tbl) != "unknown"]


## SVM parameter optimisation (using times = 10 for demonstration, usually 100)
svm_params <- svmOptimisation(object = unstim_msn,
                              fcol = "markers",
                              times = 10,
                              xval = 5,
                              class.weights = weights,
                              seed = 399)



The output of this code is an object of class GenRegRes, a dedicated data container used to store the results of machine learning optimisation (see Breckels et al. ^11^ for more details). Since we carried out 10 iterations, we have 10 F1 score matrices, each representing the results of testing our potential sigma and cost SVM paramaters on a different 5-fold partition of the training data.

## Access F1 matrix for tested sigma and cost params in iteration 1
svm_params@f1Matrices[[1]]


##       cost
## sigma       0.0625       0.125        0.25         0.5           1           2
##    0.001 0.003065364 0.003065364 0.003065364 0.003065364 0.003065364 0.003065364
##   0.01 0.003065364 0.003065364 0.003065364 0.003065364 0.003065364 0.207445544
##   0.1  0.003065364 0.003065364 0.003065364 0.003065364 0.240067080 0.730830371
##   1    0.006526998 0.006526998 0.006526998 0.006526998 0.006526998 0.109311084
##   10   0.001385450 0.001385450 0.001385450 0.001385450 0.001385450 0.001385450
##   100  0.001385450 0.001385450 0.001385450 0.001385450 0.001385450 0.001385450
##       cost
## sigma             4          8         16
##   0.001 0.003065364 0.00311616 0.25861525
##   0.01  0.467000512 0.67812419 0.79771801
##   0.1   0.874942038 0.94929648 0.96510184
##   1     0.582051068 0.84285522 0.89429244
##   10    0.001385450 0.00138545 0.05748409
##   100   0.001385450 0.00138545 0.01530253



To see which pairs of parameters resulted in the highest F1 score for each iteration we can use the getF1Scores function. This will give us a single matrix with 10 rows.

## Check F1 scores for tested parameters
getF1Scores(object = svm_params)


##               F1 sigma cost
##   [1,] 0.9440221   0.1   16
##   [2,] 0.9621307   0.1   16
##   [3,] 0.9901235   0.1   16
##   [4,] 0.9800712   0.1   16
##   [5,] 0.9768438   0.1   16
##   [6,] 0.9451423   0.1   16
##   [7,] 0.9873307   0.1   16
##   [8,] 0.9354428   0.1   16
##   [9,] 0.9774255   0.1   16
##  [10,] 0.9598493   0.1   16



We can also use the f1Count function to generate a table of all parameter combinations and the number of times (out of 10) they resulted in an F1 score above a given threshold. Here, we use t = 0.6 to count combinations which resulted in an F1 score > 0.6.

## Look at param combinations and how many times they result in F1 > 0.6
f1Count(svm_params, t = 0.6)


##     16
## 0.1 10



From this output we can see that we only have one possible parameter combination which is a sigma of 16 and cost of 0.1. This means that these parameters were the best for all 10 iterations of our training. However, in cases where there are multiple possible parameter combinations, the getParams function can be used to automatically return the *best* parameters.

## Return optimal parameters
getParams(object = svm_params)


## sigma  cost
##   0.1  16.0



**Protein localisation classification using an SVM**: Now that we have our optimised parameters, we can apply the SVM model to our data using the svmClassification function. We pass the optimised values of sigma and cost and again provide our weights to class.weights.

## Carry out SVM classification using optimised parameters
## Set a random seed for reproducibility set.seed(399)
unstim_msn <- svmClassification(object = unstim_msn,
                                fcol = "markers",
                                sigma = 0.1,
                                cost = 16,
                                class.weights = weights)


## [1] "markers"



After carrying out the SVM classification, we now have two new columns added to the fData of our MSnSet. The first of these is the svm column which contains the classification result i.e., the localisation prediction for each protein.

## Look at svm classification column
unstim_msn %>%
  fData() %>%
  pull(svm) %>%
  table()


## .
##          chromatin           cytosol               ER              ERGIC
##                180              1424              761                154
##                 GA          lysosome    mitochondrion            nucleus
##                138                79              688               1386
##         peroxisome                PM       proteasome ribosome/complexes
##                 21               366               73                431



The second new column that we have is the svm.scores column. Whilst SVMs do not directly provide probability estimates for their classifications, the algorithm does output an assignment score indicating the distance of each protein from the decision boundary.

## Look at svm.score column
unstim_msn %>%
  fData() %>%
  pull(svm.scores) %>%
  summary()


##   Min. 1st Qu.  Median    Mean 3rd Qu.    Max.
## 0.1702  0.5679  0.8938  0.7770  1.0000  1.0000



We can see that all the scores lie between 0 and 1. These scores are more challenging to interpret than standard probabilities but the larger the positive SVM score, the further away from the decision boundary the protein was. This typically corresponds to having more confidence in classifications with a greater SVM score.

**Thresholding protein localisation classification using an SVM:** As we saw above, all proteins have been classified to one of the subcellular compartments included in our training data. However, when applying a supervised machine learning algorithm, it is standard practice to set a specific confidence threshold at which we accept new assignments. Any protein with a confidence below this threshold will receive the final classification of "unknown". The way in which confidence is defined differs between classification algorithms. In the case of SVMs, we rely on the SVM score to set our thresholds.

Let’s plot a boxplot of the SVM scores of proteins allocated to each subcellular compartment. We first use the unknownMSnSet function to return an MSnSet with the marker proteins removed, since these would all have an F1 score of 1 and cannot inform us about the success of the classifier.

## Remove markers
unstim_preds <- unknownMSnSet(object = unstim_msn)

## Visualise svm scores per organelle
unstim_preds %>%
  fData() %>%
  as_tibble() %>%
  ggplot(aes(x = svm, y = svm.scores)) +
  geom_boxplot() +
  labs(x = "SVM classification", y = "SVM score") +
  theme_bw() +
  theme(axis.text.x = element_text(angle = 45, hjust = 1))

**Figure 20.**
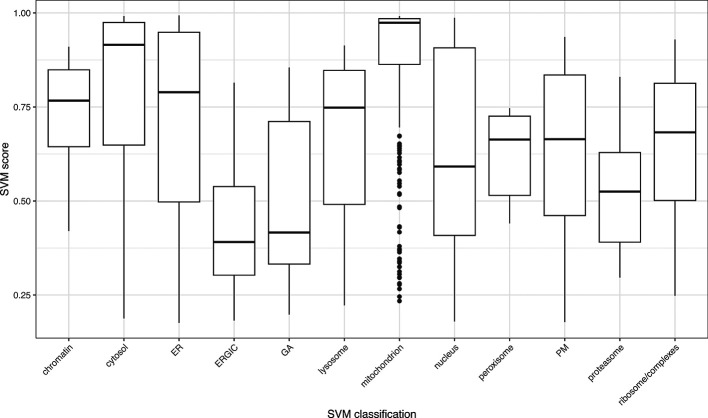
Boxplot showing the distribution of class (marker)-specific SVM scores output from running an SVM classifier on the unstimulated data.

We can see that each compartment displays a different distribution of SVM scores ( Figure 20) and, therefore, applying a global score threshold would not be appropriate here. Instead, there are several ways in which users can apply compartment-specific thresholding. One common approach is to manually set a compartment-specific FDR to allow up to 5% false discoveries, for example. This would involve putting the proteins localised to each compartment in descending order by their SVM score before going down the list to find the SVM score at which 5% of proteins are incorrectly classified based on reliable subcellular databases and prior knowledge from the literature. Unfortunately, such an approach can never be completely objective and is limited to organisms with well annotated subcellular proteomes. An alternative method would be to threshold each compartment at a set percentile of its SVM scores. Here, we will demonstrate the latter approach using the orgQuants and getPredictions functions within pRoloc.

The orgQuants function takes an MSnSet containing classification results as its input. We pass the column in our fData where the classification assignments are stored (here "svm"), the score column ( "svm.scores") and the marker column ( "markers"). Finally, we use the t argument to specify our threshold quantile. The quantile threshold is ultimately up to the user and will depend on (1) how exploratory the analysis aims to be, and (2) the data quality and resolution. In reality, we recommend testing multiple thresholds. Here, we demonstrate the use of a 0.75 quantile threshold.

## Get organelle-specific quantile SVM scores
score_thresholds <- orgQuants(object = unstim_msn,
                              fcol = "svm",
                              scol = "svm.scores",
                              mcol = "markers",
                              t = 0.75)


##          chromatin           cytosol               ER              ERGIC
##          0.8482371         0.9746572        0.9465950          0.5395450
##                 GA          lysosome    mitochondrion            nucleus
##          0.7060770         0.8467634        0.9847589          0.9054268
##         peroxisome                PM       proteasome ribosome/complexes
##          0.7294495         0.8298566        0.6280824          0.8122768



We can now use these quantile-based score thresholds in the getPredictions function.

## Use organelle-specific quantiles to get thresholded localisation predictions
unstim_msn <- getPredictions(object = unstim_msn,
                             fcol = "svm",
                             scol = "svm.scores",
                             mcol = "markers",
                             t = score_thresholds)


## ans
##          chromatin           cytosol               ER              ERGIC
##                 76               634              395                 50
##                 GA          lysosome    mitochondrion            nucleus
##                 52                44              444                515
##         peroxisome                PM       proteasome ribosome/complexes
##                 17               127               43                156
##            unknown
##               3150



After thresholding we have a new column in our fData called svm.pred. This column contains the localisation allocations after thresholding. We also get a summary output when we applied the getPredictions function showing us the number of proteins classified to each subcellular compartment as well as the 3150 proteins which remain "unknown".

**Why would proteins not meet the classification threshold and remain unknown?** After thresholding our SVM classifications we have 3150 proteins which were not assigned to one of the subcellular compartments and instead remain as “ unknown”. There are a number of reasons for which proteins may remain unknown after classification. Firstly, the subcellular structure and compartmentalisation of a cell is extremely complex and cannot be completely represented by our labelled training data (markers). For instance, some subcellular structures may have too few well-established marker proteins to be included in classification. This could be due to lack of prior knowledge or absence of a unique protein profile, as seen with p-bodies and stress granules, niches which are defined by many of the same proteins. Secondly, a large proportion of the proteome is known to reside in multiple subcellular niches within a given cell at a given time. ^40^ The abundance profiles of these multi-localised proteins can represent a mixture of their localistions and, therefore, no longer match the marker profiles of any given compartment. Further, since correlation profiling experiments utilise bulk proteomics, the observed protein abundance profiles represent an average of the population. Proteins which differ in subcellular localisation across a heterogeneous starting population (e.g., due to differences in cell cycle status) could also display mixed localisation profiles and ultimately remain classified as “ unknown”.

### Classification using TAGM-MAP

As an alternative to protein localisation classification using a classical SVM approach, users can also make use of newer Bayesian models available within pRoloc. The main advantages of applying a Bayesian framework are that (1) protein localisation predictions come with quantified uncertainties which are much more interpretable than SVM scores, and (2) it is possible to gain information about protein multi-localisations rather than simply accepting these proteins as “ unknown”. These advantages will be exemplified in this section.

Both Bayesian classifiers available within pRoloc are based on a t-augmented Gaussian mixture (TAGM) model, as outlined in Crook et al. ^35^ The first implementation of this model is referred to as TAGM-MAP as it uses *maximum a posteriori* estimates of posterior localistion probabilities. In contrast to TAGM-MCMC, the second Bayesian model which will be introduced later, TAGM-MAP does not sample the entire posterior distribution but instead uses an extended version of the expectation maximisation (EM) algorithm for inference. In terms of user experience, this means that TAGM-MAP is faster and requires less computational power. However, it also means that this algorithm will only output the most probable protein localisation and its associated uncertainty score, not the probability of localisation per compartment.

**Optimising TAGM-MAP model parameters:** The first step in using TAGM-MAP is executing the tagmMapTrain function. We run the algorithm for 100 iterations (the default, numIter = 100). We set a seed to ensure reproducibility and pass our marker annotated MSnSet to the function.

## Optimise the tagm-map model
set.seed(2)
map_params <- tagmMapTrain(unstim_msn, numIter = 100)



The output of the tagmMapTrain function is an object of class MAPParams which contains the optimised model parameters. Importantly, before applying these parameters we need to check that the model has converged. The TAGM-MAP expectation maximisation (EM) algorithm iterates between an expectation and maximisation step until the log-posterior is stable. To check that the model successfully reached this status, we use the logPosteriors function to extract the log-posteriors at each iteration and pass this to the plot function.

## Plot to visualise convergence
map_params %>%
  logPosteriors %>%
  plot(type = "b", col = "blue", cex = 0.3,
       ylab = "log-posterior",
       xlab = "Iteration")



**Figure 21.**
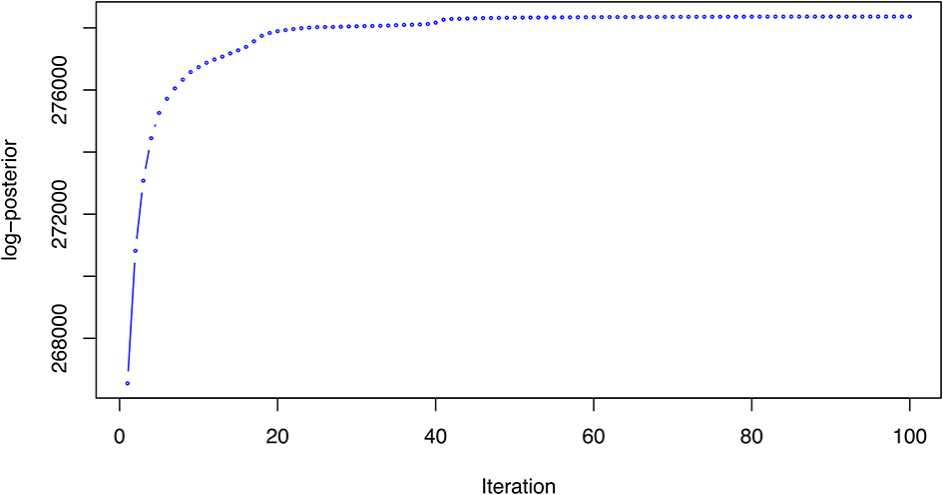
Log-posteriors from running TAGM-MAP. The log-posteriors at each iteration of the EM algorithm demonstrating convergence.

From Figure 21 we can see that the value of the log-posterior increased until it stopped changing, thus indicating that the TAGM-MAP model has converged. This means that it is suitable to continue with our optimised parameters. For further details of the algorithm we refer readers to Crook et al. ^12^

**Protein localisation classification using TAGM-MAP:** Next, we apply the optimised TAGM-MAP parameters to carry out protein localisation classification via the tagmMapPredict function. We pass our MSnSet object and the object storing our parameters, here called map_params.

## Predict protein localisation using converged parameters
unstim_msn <- tagmMapPredict(object = unstim_msn, params = map_params)



The tagmMapPredict function adds three new columns to our MSnSet.

## Check which columns we have in the fData
unstim_msn %>%
  fvarLabels()



The first is tagm.map.allocation, the column which contains the most probable localisation classification for each protein. The second column, tagm.map.probability stores the probability that the associated classification is correct. Finally, the tagm.map.outlier column contains the probability that the protein is an outlier.

Let’s take a look at the initial classification results.

## Check overall classifications prior to thresholding
unstim_msn %>%
  fData() %>%
  pull(tagm.map.allocation) %>%
  table()


## .
##          chromatin           cytosol               ER              ERGIC
##                496              1151              560                 30
##                 GA          lysosome    mitochondrion            nucleus
##                 75                92              639                725
##         peroxisome                PM       proteasome ribosome/complexes
##                 21               927              896                 89



We can also look at the range of probabilities given for organelle classifications and outlier detection.

## Check range of probabilities for compartment localisations
unstim_msn %>%
  fData() %>%
  pull(tagm.map.probability) %>%
  summary()


##   Min. 1st Qu.  Median   Mean  3rd Qu.   Max.
## 0.0000 0.6932  0.9998  0.7577  1.0000  1.0000


## Check range of probabilities for outliers
unstim_msn %>%
  fData() %>%
  pull(tagm.map.outlier) %>%
  summary()


##      Min.   1st Qu.    Median      Mean    3rd Qu.     Max.
## 0.000e+00 0.000e+00 2.918e-05 2.356e-01 2.058e-01 1.000e+00



**Thresholding protein localisation classification using TAGM-MAP:** As was the case when applying an SVM model, we still need to apply thresholding to the initial classification results to ensure confidence in our results. Again, proteins which do not meet our defined threshold will receive the final classification of "unknown". Thresholding of TAGM-MAP outputs can be achieved by combining the probability of a predicted compartment classification being correct with the probability that a protein belongs to an outlier component. In the code chunk below we create a new column in the fData of our MSnSet called overall_prob. This value is calculated as the probability of the compartment classification multiplied by the inverse of the probability that a protein is an outlier. Hence, a larger overall_prob indicates a higher confidence in our classification. We then use the getPredictions function to update our classification predictions based on our score column ( scol) overall_prob with a threshold ( t) of 0.999.

## Store classification and outlier probabilities
tagm_prob <- fData(unstim_msn)[, "tagm.map.probability"]
tagm_out <- 1 - fData(unstim_msn)[, "tagm.map.outlier"]

## Create new column containing overall probability
fData(unstim_msn)[, "overall_prob"] <- tagm_prob * tagm_out

## Set prediction thresholds on overall probability
unstim_msn <- getPredictions(unstim_msn,
                             fcol = "tagm.map.allocation",
                             scol = "overall_prob",
                             t = 0.999)


## ans
##          chromatin           cytosol               ER              ERGIC
##                187               662              410                 24
##                 GA          lysosome    mitochondrion            nucleus
##                 52                72              561                409
##         peroxisome                PM       proteasome ribosome/complexes
##                 18               302              311                 66
##            unknown
##               2627



As above, the getPredictions function has printed a summary of the thresholded classification results, which are stored in a new column called tagm.map.allocation.pred.

### Classification using TAGM-MCMC

The pRoloc package contains a second TAGM-based Bayesian algorithm for protein localisation prediction. This method, termed TAGM-MCMC, is based on a Markov-chain Monte Carlo sampling method and can be used to obtain a full posterior localisation probability distribution for each protein rather than a single point estimate. This means that in addition to reporting the most probable localisation classification for each protein, the TAGM-MCMC algorithm is able to give the full probability distribution of a protein being classified to each of the included subcellular compartments. This is particularly useful for users wishing to investigate protein multi-localisations. Ultimately, however, this richer analysis comes with slower implementation and the need for more computational power. Users will require access to moderate computational resources and expect the analysis to run for hours-days depending on available backend.

**Optimising TAGM-MCMC model parameters:** As was the case for TAGM-MAP, the first step in the application of TAGM-MCMC is to train the algorithm. This is done by passing our MSnSet to the tagmMcmcTrain function. In the next code chunk we load a pre-computed TAGM-MCMC model as generating a model locally can take several hours when run for thousands of iterations (as advised and discussed below).

load("tagm_mcmc_params.rda", verbose = TRUE)


## Loading objects:
##   mcmc_model



The model was generated from running the following code. It took approximately 5 hours on a HPC machine using 20 cores.

## Training performed using and HPC
mcmc_model <- tagmMcmcTrain(object = unstim_msn,
                            numIter = 10000, # typically recommend 10,000
                            burnin = 1000, # start with 1000
                            thin = 20,
                            numChains = 4,
                            S0 = diag(0.001, nrow = 24))



In the code chunk above we set the number of iterations to 10,000. In general, we recommend specifying numIter to 10,000 or greater to achieve model convergence. Further, when applying an MCMC sampling method it is necessary to define the numChains argument to specify how many parallel Markov chains we wish to run. We recommend starting with 4 chains and running more if an assessment of the chains does not yield satisfactory results. We also need to specify the burn-in parameter i.e. burnin, which in Bayesian statistics is referred to as the “warm-up” period of time (iterations) before we start collecting samples (and reach equilibrium). Finally, we set thin which refers to thinning. This consists of picking separated points from the sample, at each *k*th step and can help reduce size of the model. This is a consideration as these models can get large ~1GB. We refer users to Crook et al. ^12^ for more details and to consult the tagmMcmcTrain documentation file in pRoloc by typing ?tagmMcmcTrain in the console of RStudio.

If we examine the object we’ve generated we see it is of class MCMCParams containing the model parameters.

mcmc_model


## Object of class "MCMCParams"
## Method: TAGM.MCMC
## Number of chains: 4



**Assessing convergence in MCMC models:** Bayesian MCMC models are only reliable if Markov chains adequately converge and sample from the joint posterior distribution. There is a whole field of research dedicated to assessing Markov chain convergence and Crook et al. ^12^ has implemented some functions to help users in the context of the TAGM-MCMC algorithm within the pRoloc framework. We refer users to Crook’s Bayesian workflow for a more comprehensive application. ^12^ However, for completeness we show here two user-friendly methods to help assess chain convergence: (1) computation of the Gelman and Rubin diagnostic and (2) visualisation of the model characteristics via trace and density plots. These two methods will also be re-visited later in this workflow when we demonstrate how to use the BANDLE method to interrogate protein differential localisation.

First we run the helper function mcmc_get_outliers to extract the number of outliers at each MCMC iteration.

mcmc_output <- mcmc_get_outliers(mcmc_model)



Let’s start with the Gelman which allows us to compare the inter and intra chain variances. If the chains have converged the ratio of these quantities should be less than 1.2.

## Calculate Gelman and Rubin diagnostic (for all chains together)
coda:::gelman.diag(mcmc_output, transform = FALSE)


## Potential scale reduction factors:
##
##      Point est. Upper C.I.
## [1,]          1          1



We see the Gelman is 1, which is our first indication of convergence. If users see values higher than 1.2 there may be one or several bad chains. It is possible to compute the Gelman for different combinations of chains (excluding some, keeping others). We can visually assess the chains to give an idea of what is driving these statistics.

Assessing model characteristics for convergence via trace and density plots is common practice in Bayesian analysis. Trace and density plots are subjective but can be used to help visually assess the sample path of the chains. Textbooks on Bayesian inference often tell us that a good trace plot should look like a “hairy” or “fuzzy caterpillar”. We can also look at the mean number of allocations at each iteration of the MCMC algorithm, across iterations and also express this a probability density. We expect the number of allocations (and in turn the number of outliers) to have reached a stable equilibrium. Practically, this translates to visualising a normally distributed plot centered around roughly the same number of outliers in each chain.

In the below code chunk we plot trace and density plots for each chain.

## Save number of chains
nChains <- length(mcmc_output)

## Plot trace and density plots per chain
par(mfrow = c(4, 2))
for (i in seq_len(nChains))
  plot(mcmc_output[[i]], main = paste("Chain", i),
       auto.layout = FALSE, col = i)



**Figure 22.**
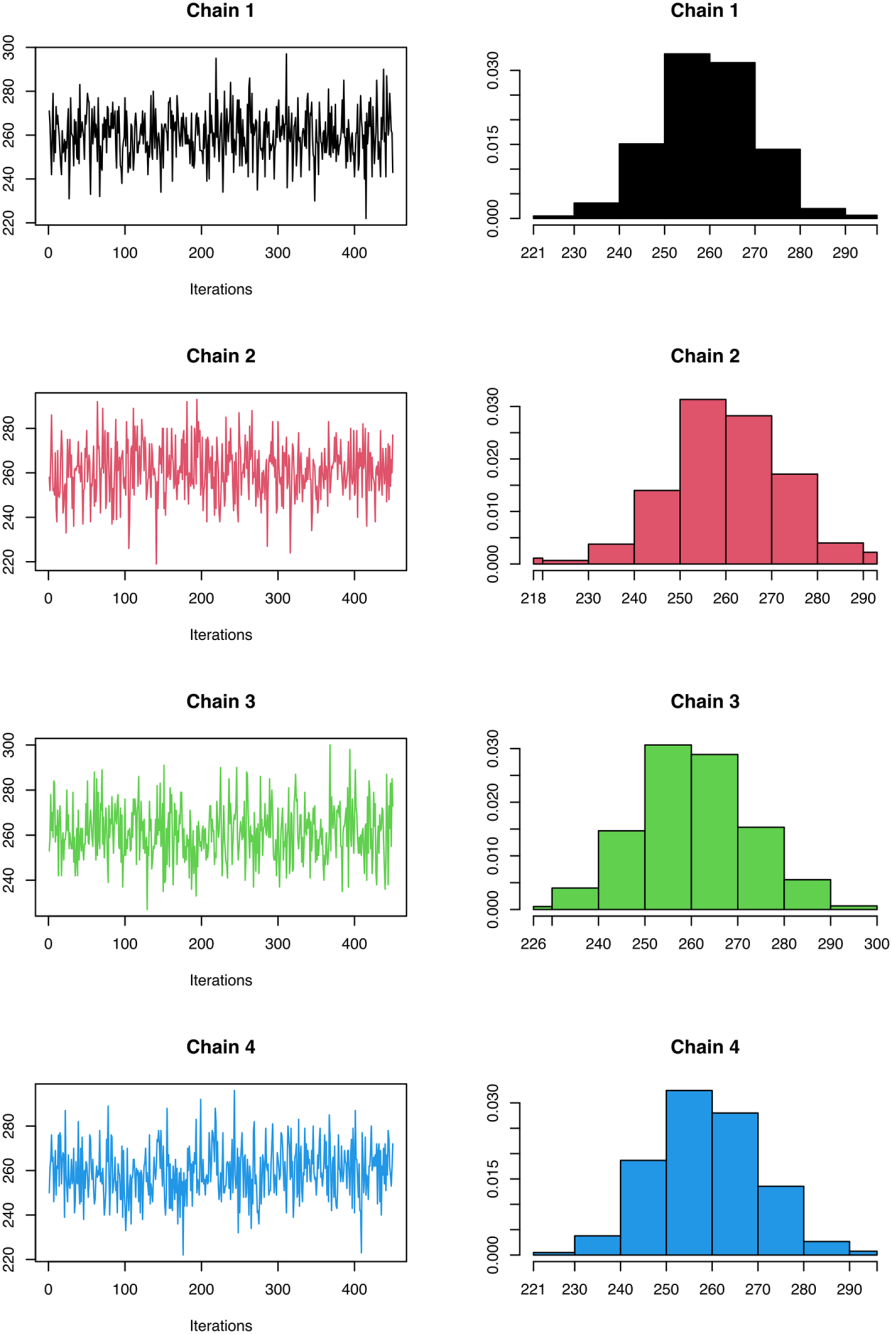
Trace (left) and density (right) plots for the MCMC chains output from running a TAGM-MCMC analysis.

The trace plots show how the number of outliers varies across each iteration ( Figure 22, left). We point out here that although we ran the algorithm for 10,000 iterations, we had a burin of 1000, and thinned (discarded) data at every 20 iterations, this leaves 450 samples per chain (x-axis, left plots). The density plots show the number of outliers expressed as a probability density ( Figure 22, right). We see that all chains are centered around 260 outliers per run. If there was a chain with wildly different outliers (i.e. centered around a different value) to the others, we could consider removing this chain and re-computing the the Gelman diagnostics.

For the sake of demonstration to show users how to discard a bad chain, let’s remove chain 2. Taking 3 chains forward for modelling is sufficient. We remove the chain and call the new object mcmc_converged.

## Save index of chain to remove
removeChain <- 2


## Create new object with desired chain(s) removed
mcmc_converged <- mcmc_model[-removeChain]



We can confirm that the chain has been removed by checking the length of the new object mcmc_converged.

## Check number of chains in new object
length(mcmc_converged)


## [1] 3



We verify that we now have 3 chains remaining. Lastly, we pool the chains together to generate one long chain from which we can sample.

## Pool remaining chains into a single chain for sampling
mcmc_converged <- mcmc_pool_chains(mcmc_converged)



**Protein localisation prediction using TAGM-MCMC:** Now that we have assessed our model we can process the chains using tagmMcmcProcess and perform protein localisation prediction using tagmPredict. We pass the processed parameters to the params argument and specify probJoint = TRUE to indicate we would like to return the predicted probabilities across all subcellular classes.

## Process MCMCParams object
p <- tagmMcmcProcess(mcmc_converged)


## Use optimised tagm-mcmc parameters to classify protein localisation
unstim_msn <- tagmPredict(unstim_msn, params = p, probJoint = TRUE)

## Check the column names in our fData
unstim_msn %>%
  fvarLabels()


##  [1] "Checked"                             "Tags"
##  [3] "Confidence"                          "Identifying.Node.Type"
##  [5] "PSM.Ambiguity"                       "Contaminant"
##  [7] "Number.of.Proteins"                  "Master.Protein.Accessions"
##  [9] "Master.Protein.Descriptions"         "Protein.Accessions"
## [11] "Protein.Descriptions"                "Delta.Cn"
## [13] "Rank"                                "Search.Engine.Rank"
## [15] "Concatenated.Rank"                   "Ions.Matched"
## [17] "Matched.Ions"                        "Total.Ions"
## [19] "Activation.Type"                     "MS.Order"
## [21] "Quan.Info"                           "Number.of.Protein.Groups"
## [23] "hc"                                  "hdb_cluster_id"
## [25] "hdb_cluster_prob"                    "markers_initial"
## [27] "markers"                             "svm"
## [29] "svm.scores"                          "svm.pred"
## [31] "tagm.map.allocation"                 "tagm.map.probability"
## [33] "tagm.map.outlier"                    "overall_prob"
## [35] "tagm.map.allocation.pred"            "tagm.mcmc.allocation"
## [37] "tagm.mcmc.probability"               "tagm.mcmc.probability.lowerquantile"
## [39] "tagm.mcmc.probability.upperquantile" "tagm.mcmc.mean.shannon"
## [41] "tagm.mcmc.outlier"                   "tagm.mcmc.joint"



We see the MsnSet has been populated with the MCMC results as we have 7 new columns appended to the fData.

**Thresholding protein localisation classifications using TAGM-MCMC:** We again perform thresholding on our results, as we did for the previous two classification methods. As per the MAP method, the MCMC method outputs the mean posterior probability for the protein subcellular localisation allocations. This is located in the tagm.mcmc.probability column of the feature data. Other useful summaries are available with the MCMC method such as the tagm.mcmc.outlier which is the posterior probability for the protein belonging to the outlier component. The Monte-Carlo average Shannon entropy is also computed and can be found in the column tagm.mcmc.mean.shannon and is an orthogonal measure of uncertainty.

## Examine probabilities
unstim_msn %>%
  fData() %>%
  pull(tagm.mcmc.probability) %>%
  summary()


##    Min. 1st Qu.  Median    Mean  3rd Qu.    Max.
##  0.3257  0.9880  1.0000  0.9529   1.0000  1.0000


## Summary of outlier probabilities
unstim_msn %>%
  fData() %>%
  pull(tagm.mcmc.outlier) %>%
  summary()


##       Min.    1st Qu.     Median       Mean    3rd Qu.       Max.
##  0.000e+00  0.000e+00  1.220e-06  4.563e-02  6.478e-04  1.000e+00


## Summary of Shannon entropy
unstim_msn %>%
  fData() %>%
  pull(tagm.mcmc.mean.shannon) %>%
  summary()


##      Min.   1 stQu.    Median      Mean   3rd Qu.      Max.
## 0.0000000 0.0000000 0.0001437 0.1003307 0.0584145 1.4212035



In the subsequent code chunk we again threshold on the posterior probability and outlier probability as we did in the TAGM-MAP section, storing the results in the column “overall_prob” and thresholding on this output.

## Store allocation and outlier probabilities
tagm_prob <- fData(unstim_msn)[, "tagm.mcmc.probability"]
tagm_out <- 1 - fData(unstim_msn)[, "tagm.mcmc.outlier"]

## Create a new column containing overall probability
fData(unstim_msn)[, "overall_prob"] <- tagm_prob * tagm_out

## Set prediction thresholds based on overall probability
unstim_msn <- getPredictions(unstim_msn,
                             fcol = "tagm.mcmc.allocation",
                             scol = "overall_prob",
                             t = 0.999)


## ans
##          chromatin           cytosol               ER              ERGIC
##                225               559              344                294
##                 GA          lysosome    mitochondrion            nucleus
##                181                61              500                476
##         peroxisome                PM       proteasome ribosome/complexes
##                 19               290              192                179
##            unknown
##               2381



In machine learning setting decision thresholds is non-trivial and can be especially tricky for imbalanced datasets. ^41^ Class imbalance can lead to over-classification and it is important to try to manage this scenario with thresholding as we have done here by thresholding on not only the posterior probability but also the outlier probability that is generated with the TAGM models. As mentioned above, the Shannon entropy can also be used in thresholding and should be considered if over-classification is occurring in the data.

**Figure 23.**
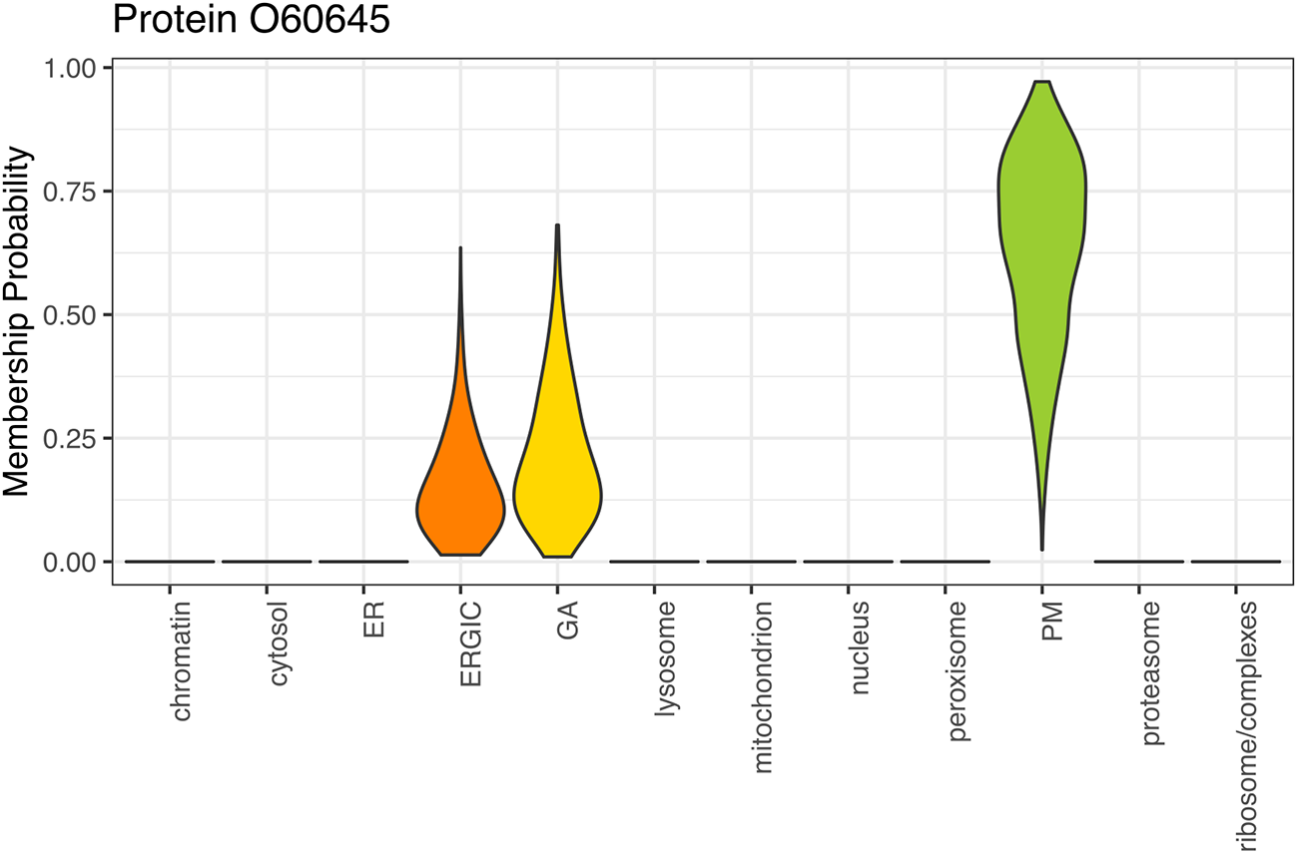
A membership probability plot for protein O60645. The violin plot displays the full posterior probability distribution across all subcellular niches included in the training data.

**Extracting multi-localised proteins:** One of the advantages of using fully Bayesian methods is the ability to extract the full probability distribution of a protein across multiple subcellular classes. This is important as studies have suggested that over half of the proteome can reside in multiple locations in the cell. ^40^ In the Bayesian workflow from Crook et al. ^12^ the authors show how to draw upon the Shannon entropy to pull out proteins that have uncertainty in their location. The probability distribution of proteins of interest can be visualised using the plot generic method for TAGM-MCMC objects. In the code chunk below we show how to generate a violin plot for a given protein of interest. We plot protein O60645, an exocyst complex protein known to be involved in vesicle transport by tethering secretory vesicles to the plasma membrane. The violin plot reveals uncertainty in the localisation of this protein between the ERGIC complex, golgi apparatus and plasma membrane, reflective of its function ( Figure 23).

plot(mcmc_converged, "O60645")

### Generating a subcellular spatial map

Regardless of the algorithm used, the result of classification is a list of proteins and their predicted subcellular localisations. As was the case for markers, these results can be visualised using dimensionality reduction methods via the plot2D function to create a “subcellular spatial map” of the data. It is also important to remember to plot the protein correlation profiles alongside maps of the data to aid interpretation and verify resolution.

This time, we pass our MSnSet and specify the fcol as the column containing our thresholded localisation results. It is up to the user which dimensionality reduction method is applied. To see the full list of visualisation methods available for use with plot2D see plot2Dmethods which are also listed in the plot2D documentation. See ?plot2D for details.

plot2Dmethods


## [1] "PCA"    "MDS"   "kpca"   "lda"   "t-SNE"   "UMAP"   "nipals" "hexbin"
## [9] "none"   "scree"



**Figure 24.**
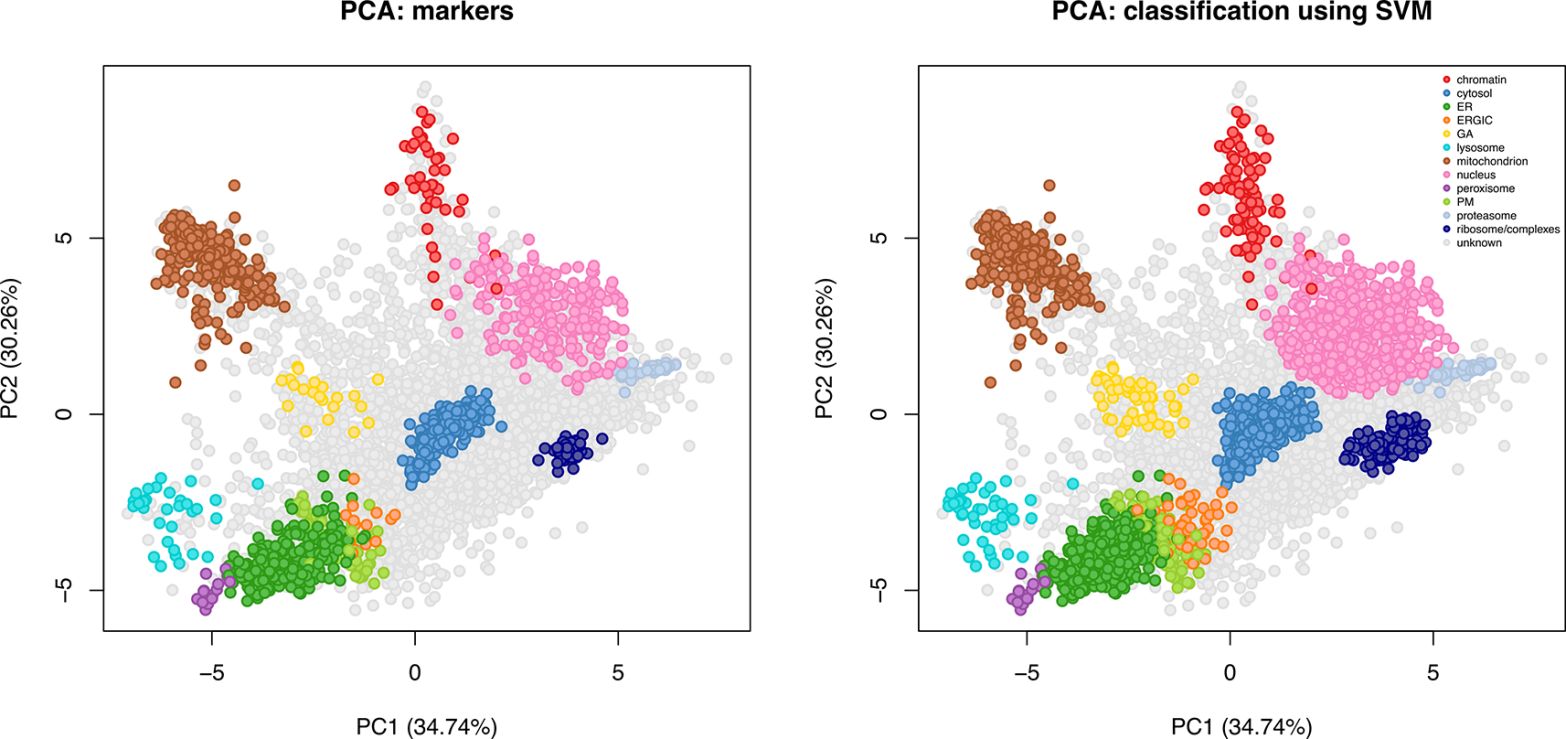
PCA plots of the unstimulated data annotated with marker proteins (left) and classification results from running a SVM (right). One point represents one protein, and proteins are annotated by colour according to their subcellular localisation.

We can plot the data using the default plot2D setting which is a PCA visualisation and tell plot2D where the markers and results are located ( Figure 24, left). For example, to plot the markers we would pass fcol = "markers" and to plot the results of the SVM classification after thresholding we would pass fcol = "svm.pred" ( Figure 24, right).

par(mfrow = c(1, 2))
unstim_msn %>%
  plot2D(main = "PCA: markers")
unstim_msn %>%
  plot2D(fcol = "svm.pred", main = "PCA: classification using SVM")
unstim_msn %>%
  addLegend(where = "topright", cex = .5)



Some non-linear dimensionality reduction methods for example t-SNE and UMAP, among others, can take from a minute to tens of minutes to compute. They are also stochastic, so it is good practice to set a seed for reproducibility of the visualisation and also to pre-compute the coordinates and pass the resultant matrix to plot2D.

In the below code chunk, we show how to pre-compute the coordinates for PCA, UMAP, t-SNE and linear discriminant analysis (LDA).

## Pre-compute coordinates for PCA
## PCA is deterministic so we do not need to set a seed
pcas <- unstim_msn %>% plot2D(method = "PCA", plot = FALSE)

## UMAP
set.seed(399)
umap <- unstim_msn %>%
  plot2D(method = "UMAP", plot = FALSE)

## t-SNE
set.seed(399)
tsne <- unstim_msn %>%
  plot2D(method = "t-SNE", plot = FALSE)

## Linear discriminant analysis
set.seed(399)
lda <- unstim_msn %>%
  plot2D(method = "lda", plot = FALSE)



**Figure 25.**
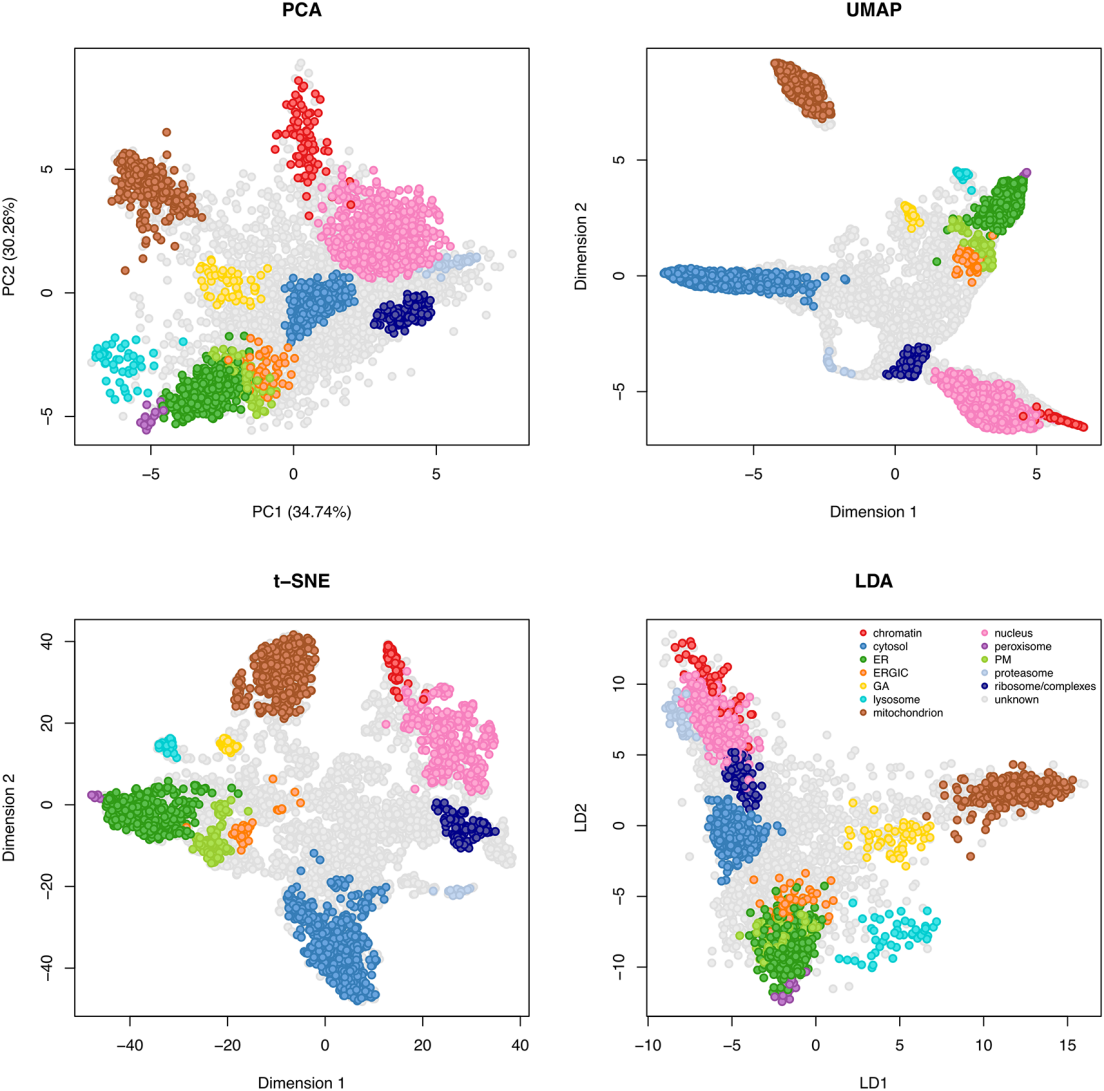
Two-dimensional visualisation of the unstimulated data using different dimensionality reduction methods. Subcellular maps generated by principal components analysis (PCA) (top left), Uniform Manifold Approximation and Projection (UMAP) (top right), t-distributed stochastic neighbor embedding (t-SNE) (bottom left) and linear discriminant analysis (LDA) (bottom right).

We can now pass these coordinates to plot2D to visualise the data ( Figure 25). Since they have been computed once, we don’t have to compute them again for subsequent visualisations so we can pass method = “none”. We pass fcol = “svm.pred” to visualise the results from the SVM classification.

par(mfrow = c(2, 2))
plot2D(pcas, method = "none", methargs = list(unstim_msn),
       fcol = "svm.pred", main = "PCA")
plot2D(umap, method = "none", methargs = list(unstim_msn),
       fcol = "svm.pred", main = "UMAP")
plot2D(tsne, method = "none", methargs = list(unstim_msn),
       fcol = "svm.pred", main = "t-SNE")
plot2D(lda, method = "none", methargs = list(unstim_msn),
       fcol = "svm.pred", main = "LDA")
addLegend(unstim_msn, where = "topright", ncol = 2, cex = .8)



### Overlaying features of interest

Users may wish to add additional annotation to their data, for example, small protein complexes, pathways of interest or organelles not deemed to be suitable to use as training data. This may include organelles that lacked resolution in the experiment or do not have enough protein members to include as a class in the training data for machine learning. If the main aim of the experiment is to produce as comprehensive map as possible for the community it is important to add other layers of information (if these are available). One way to do this is via the addMarkers function or alternatively one could create a new column in the feature data slot manually and add information to this new column.

In the section below we show how to annotate and plot a subset of proteins of interest in the data. First, we use the pRolocVis function in pRolocGUI to access a searchable version of our fData.

## Initiate pRoloc GUI
pRolocVis(unstim_msn, fcol = "svm.pred")



**Figure 26.**
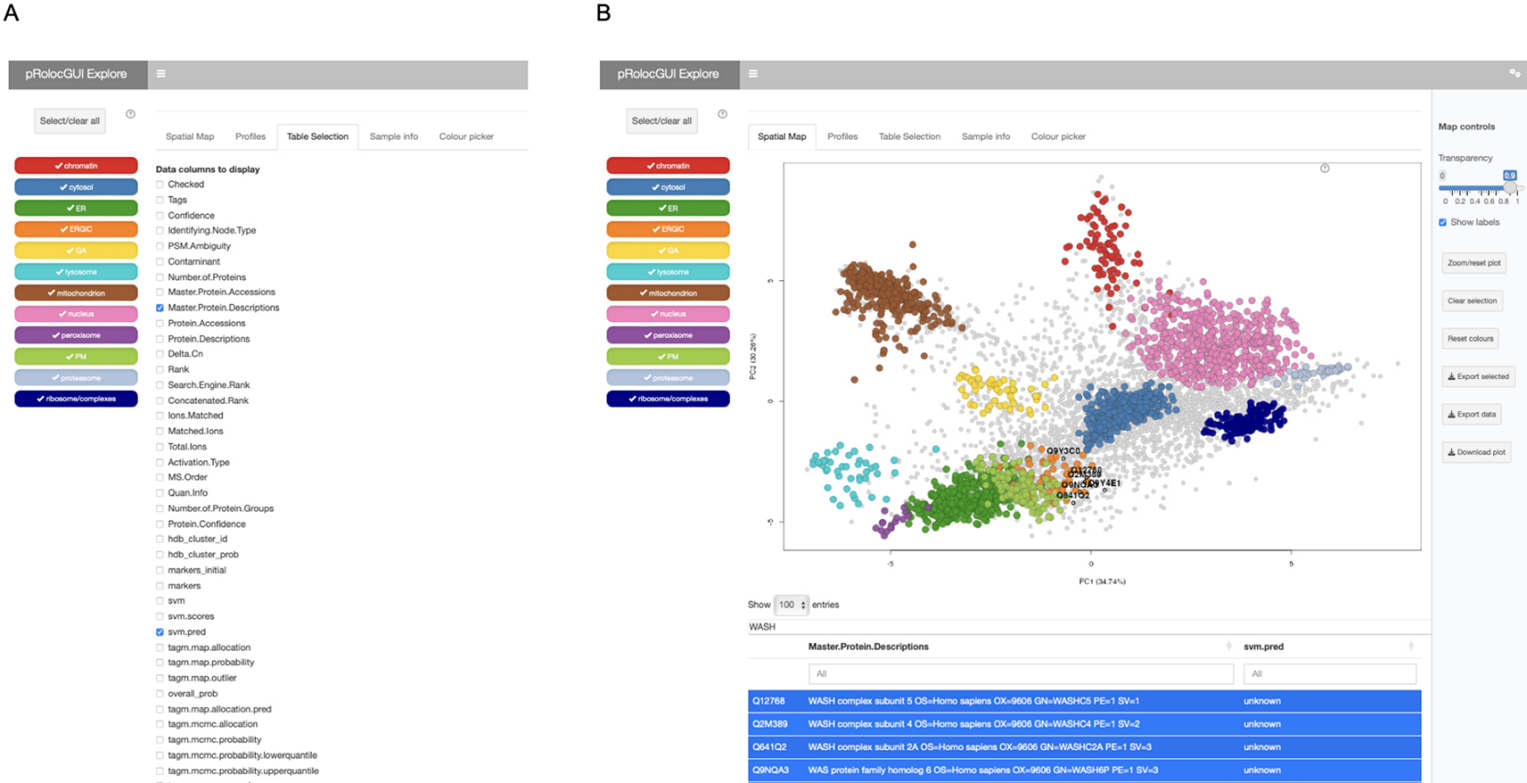
(A) Screenshot of the "Table Selection" tab and (B) Searching the data table in the "Spatial Map" tab in pRolocVis application.

Upon loading of the GUI it is helpful to navigate to the “Table Selection” tab of the app and click on the “Master.Protein.Descriptions” and a column containing subcellular localisation information, here we select “svm.pred” ( Figure 26). The protein descriptions were output with the results of our third party search in Proteome Discoverer so we advise users to be mindful that natively searching for protein descriptions will be dependent on what information is contained in the information of the data table. Users can add this information themselves from databases such as UniProt or search according to a given identifier e.g. accession number or gene name.

We search the feature data for proteins known to localise to (1) the WASH complex; a protein complex that activates the Arp2/3 complex and is involved in actin polymerization, and (2) the exosome complex.

Having identified the protein accessions for members of each complex we can define these members as a character vector.

## Create vector containing master protein accessions of proteins of interest
wash <- c("Q12768", "Q2M389", "Q641Q2", "Q9NQA3", "Q9Y3C0", "Q9Y4E1")

exosome <- c("Q06265", "Q13868", "Q15024", "Q5RKV6", "Q96B26",
             "Q9NPD3", "Q9NQT4", "Q9NQT5", "Q9Y2L1","Q9Y3B2")

**Figure 27.**
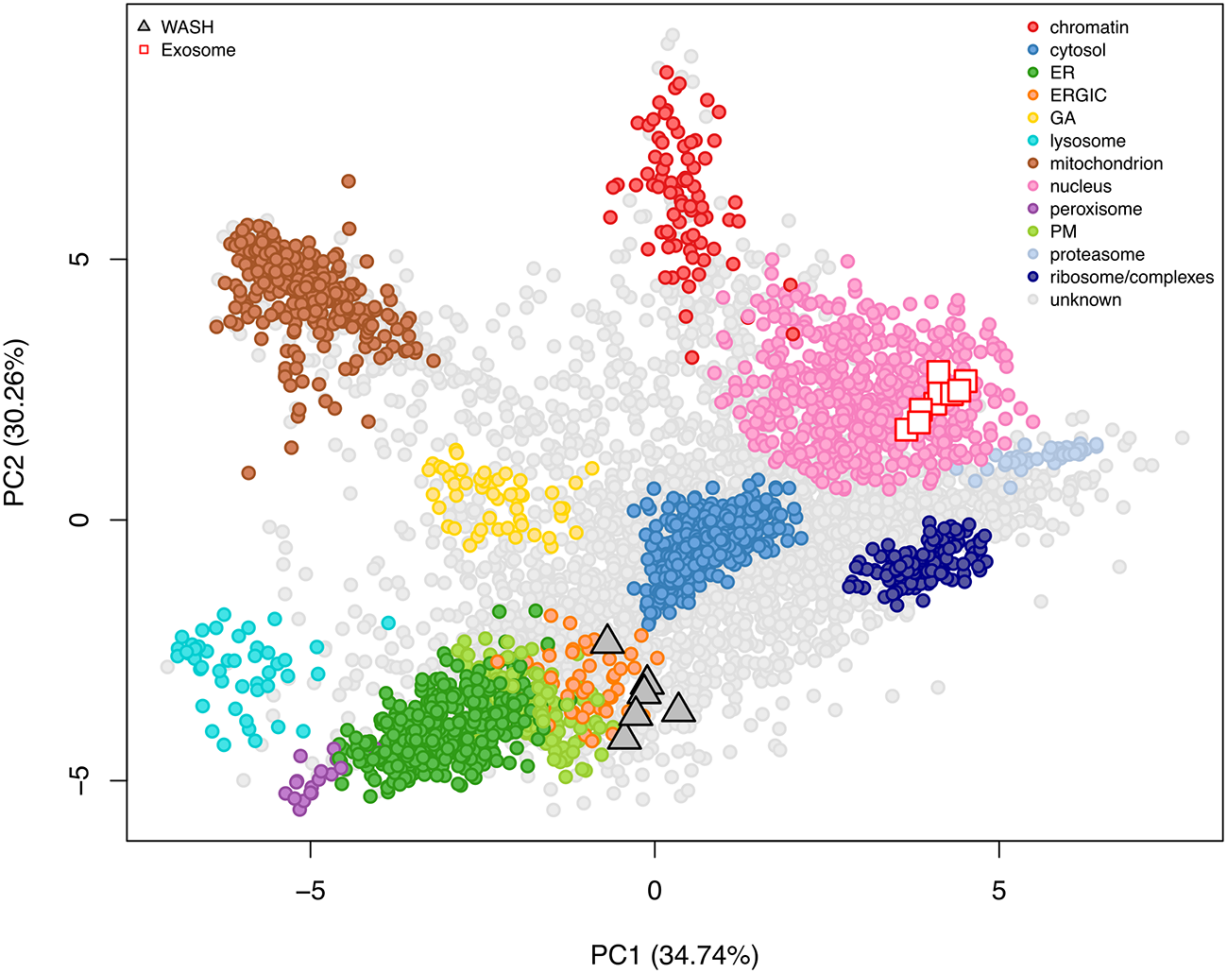
PCA plot of the unstimulated data highlighting the location of the WASH and exosome complexes alongside the protein localisation prediction results.

The location of these proteins can then be plotted using the highlightOnPlot function, as seen in Figure 27. We first use plot2D to generate a spatial map based on PCA, as done previously. We then use highlightOnPlot by passing this function our PCA coordinates ( pcas) and a vector containing features (proteins) of interest, as well as a number of arguments to specify the aesthetics of our annotations.

## Annotate proteins of interest on PCA plots
pcas %>%
  plot2D(method = "none", methargs = list(unstim_msn), fcol = "svm.pred")
highlightOnPlot(pcas, foi = wash, col = "black", bg = "grey",
                pch = 24, cex = 2, lwd = 1.5)
highlightOnPlot(pcas, foi = exosome, col = "red", bg = "white",
                pch = 22, cex = 2, lwd = 1.5)

## Generate a legend for the organelle classes
addLegend(unstim_msn, where = "topright", cex = .7)

## Generate a legend for the complexes
legend("topleft", legend = c("WASH", "Exosome"),
       col = c("black", "red"),
       pt.bg = c("grey", "white"),
       pch = c(24, 22), bty = "n", cex = .7)

Alternatively, if we wanted to plot a map with only our proteins of interested annotated, we can create a new column in the fData of the MSnSet and store these annotations.

## Initiate new column with all proteins annotated as unknown
fData(unstim_msn)[, "other.complexes"] <- "unknown"

## Overwrite the "unknown" in rows corresponding to WASH or Exosome
fData(unstim_msn)[wash, "other.complexes"] <- "WASH complex proteins"
fData(unstim_msn)[exosome, "other.complexes"] <- "Exosome complex proteins"



We can verify that these annotations have been correctly added by printing a table of the other.complexes column in the fData.

## Print table of newly generated other.complexes column
unstim_msn %>%
  fData() %>%
  pull(other.complexes) %>%
  table()


## .
## Exosome complex proteins                unknown    WASH complex proteins
##                       10                   5685                        6



Now we can pass this newly generated column to the fcol argument of plot2D and produce Figure 28.

## Plot map with proteins coloured based on other.complexes column
unstim_msn %>%
  plot2D(fcol = "other.complexes")
addLegend(unstim_msn, fcol = "other.complexes", where = "topleft")

**Figure 28.**
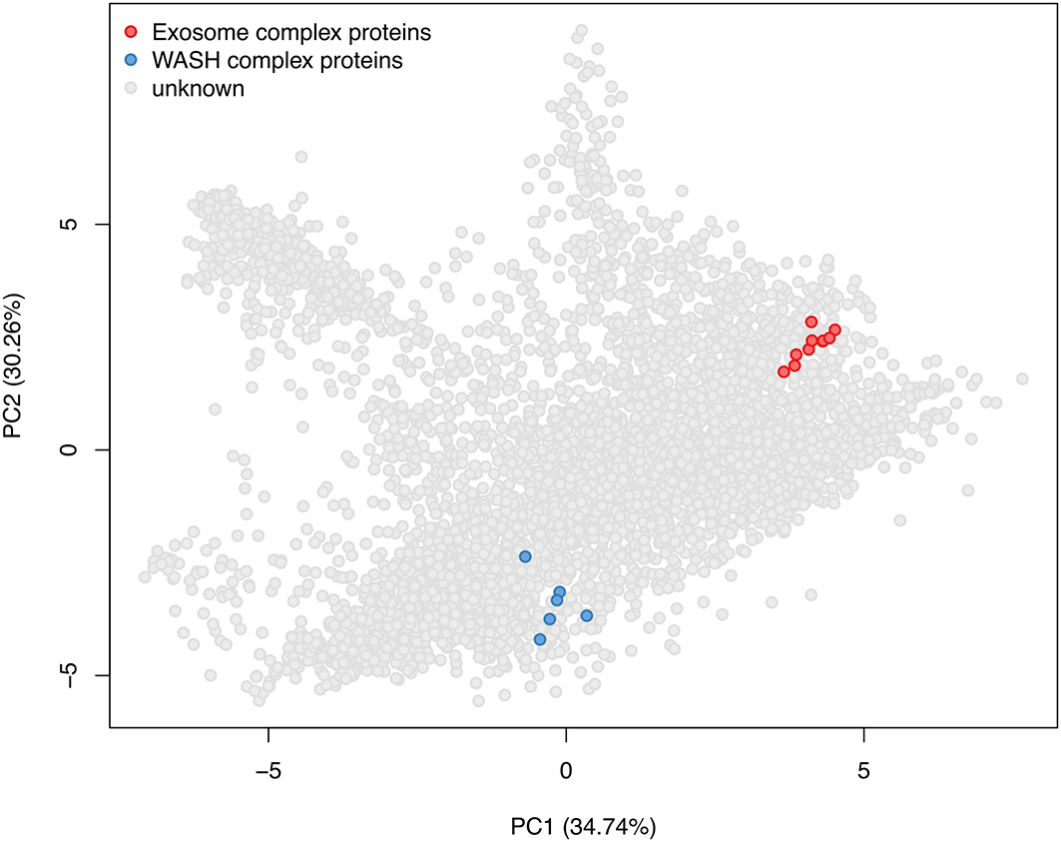
An alternative method for highlighting plotting of proteins of interest. The PCA plot of the unstimulated data is highlighting the location of the WASH and exosome complexes whilst the remaining proteins have been left unlabelled (grey).

### Verifying the results of protein localisation classification

As outlined in “Defining markers for supervised machine learning”, the curation of marker proteins and their application to localisation classification should be considered an iterative process. Having now completed classification, it is necessary to consider whether the results look realistic and subcellular compartments are accurately represented. In particular, users should plot (1) maps and (2) profiles of classified proteins. Further, we recommend using multiple dimensionality reduction methods to visualise the final maps due to the challenges with interpreting these methods, as discussed above.

## Part 3: Differential localisation for dynamic protein correlation profiling experiments

Whilst the steady-state localisation of proteins can provide vital information about functionality, these localisations are specific to the system of interest under the study conditions. Given the functional importance of protein localisation, it is no surprise that the aim has extended from profiling the spatial proteome of a single condition to comparing the spatial proteome between conditions. Indeed, correlation profiling methods have been widely applied to study protein dynamics under various conditions. ^4, 9, 13, 42– 47^

Here, we apply Bayesian ANalysis of Differential Localisation Experiments (BANDLE) ^10^ to identify proteins which are differentially localised in A549 cells upon a 12-hour exposure to 6 Gy x-ray radiation. Whilst it is possible to carry out protein localisation classification using an alternative algorithm and use BANDLE only for differential localisation prediction, for completeness we demonstrate how to extract both protein localisation and differential localisation predictions from BANDLE.

### Preparing the data for BANDLE

Before we can start using the bandle package with our data we need to extract the QFeatures data into an MSnSet. As before, we use the as command and pass the experimental set from our Qfeatures object that we wish to convert to class MSnSet. Currently, bandle works with MSnSets and lists of MSnSets.

**Extract protein-level data into MSnSet**

We can use the experiments command to view all our data stored in the QFeatures object.

experiments(qf)


## ExperimentList class object of length 32:
## [1] psms_raw_rep1: SummarizedExperiment with 115302 rows and 16 columns
## [2] psms_raw_rep2: SummarizedExperiment with 135169 rows and 16 columns
## [3] psms_raw_rep3: SummarizedExperiment with 120343 rows and 16 columns
## [4] psms_filtered_rep1: SummarizedExperiment with 79117 rows and 16 columns
## [5] psms_filtered_rep2: SummarizedExperiment with 90226 rows and 16 columns
## [6] psms_filtered_rep3: SummarizedExperiment with 80446 rows and 16 columns
## [7] psms_rep1_unstim: SummarizedExperiment with 78864 rows and 8 columns
## [8] psms_rep2_unstim: SummarizedExperiment with 90078 rows and 8 columns
## [9] psms_rep3_unstim: SummarizedExperiment with 80302 rows and 8 columns
## [10] psms_rep1_xray: SummarizedExperiment with 78794 rows and 8 columns
## [11] psms_rep2_xray: SummarizedExperiment with 89910 rows and 8 columns
## [12] psms_rep3_xray: SummarizedExperiment with 80099 rows and 8 columns
## [13] psms_rep1_unstim_norm: SummarizedExperiment with 78864 rows and 8 columns
## [14] psms_rep2_unstim_norm: SummarizedExperiment with 90078 rows and 8 columns
## [15] psms_rep3_unstim_norm: SummarizedExperiment with 80302 rows and 8 columns
## [16] psms_rep1_xray_norm: SummarizedExperiment with 78794 rows and 8 columns
## [17] psms_rep2_xray_norm: SummarizedExperiment with 89910 rows and 8 columns
## [18] psms_rep3_xray_norm: SummarizedExperiment with 80099 rows and 8 columns
## [19] psms_rep1_unstim_imputed: SummarizedExperiment with 78864 rows and 8 columns
## [20] psms_rep2_unstim_imputed: SummarizedExperiment with 90078 rows and 8 columns
## [21] psms_rep3_unstim_imputed: SummarizedExperiment with 80302 rows and 8 columns
## [22] psms_rep1_xray_imputed: SummarizedExperiment with 78794 rows and 8 columns
## [23] psms_rep2_xray_imputed: SummarizedExperiment with 89910 rows and 8 columns
## [24] psms_rep3_xray_imputed: SummarizedExperiment with 80099 rows and 8 columns
## [25] prots_rep1_unstim: SummarizedExperiment with 6446 rows and 8 columns
## [26] prots_rep2_unstim: SummarizedExperiment with 6689 rows and 8 columns
## [27] prots_rep3_unstim: SummarizedExperiment with 6375 rows and 8 columns
## [28] prots_rep1_xray: SummarizedExperiment with 6446 rows and 8 columns
## [29] prots_rep2_xray: SummarizedExperiment with 6689 rows and 8 columns
## [30] prots_rep3_xray: SummarizedExperiment with 6374 rows and 8 columns
## [31] prots_unstim: SummarizedExperiment with 5701 rows and 24 columns
## [32] prots_xray: SummarizedExperiment with 5700 rows and 24 columns



In the next code chunk, we extract the individual replicates into MSnSet containers.

## Coerce SummarizedExperiment objects to MSnSets
unstim_msn1 <- as(object = qf[["prots_rep1_unstim"]], Class = "MSnSet")
unstim_msn2 <- as(object = qf[["prots_rep2_unstim"]], Class = "MSnSet")
unstim_msn3 <- as(object = qf[["prots_rep3_unstim"]], Class = "MSnSet")
xray_msn1 <- as(object = qf[["prots_rep1_xray"]], Class = "MSnSet")
xray_msn2 <- as(object = qf[["prots_rep2_xray"]], Class = "MSnSet")
xray_msn3 <- as(object = qf[["prots_rep3_xray"]], Class = "MSnSet")


class(unstim_msn1)


## [1] "MSnSet"
## attr(,"package")
## [1] "MSnbase"



To make downstream analysis simpler we store the replicates of each condition in a list.

data <- c(unstim_msn1, unstim_msn2, unstim_msn3,
          xray_msn1, xray_msn2, xray_msn3)



### Assessing resolution and adding markers

As was the case when using machine learning for protein localisation classification, the use of BANDLE requires that the data is of high quality, contains well-resolved subcellular niches, and is annotated with a representative set of training data i.e.，a set of subcellular markers. We previously demonstrated how to assess the resolution of correlation profiling-based subcellular proteomics data and curate an appropriate marker list. Therefore, for convenience, we here use the addMarkers function to add the same markers to our individual samples as were used in part 2 for for the classification on static maps. Users who have not yet curated a confident list of markers are referred to part 2 of this workflow.

First, we extract the markers curated in part 2. These are stored in the markers column of the fData of our unstim MSnSet object. The code below will extract all values in the markers column, including the "unknowns". Since these unknowns are not markers, we remove those.

## Extract the markers curated in part 2
mrk <- fData(unstim_msn)$markers
names(mrk) <- featureNames(unstim_msn)

## Remove unknowns
mrk <- mrk[which(mrk != "unknown")]

## Print a table to check markers
table(mrk)


## mrk
##          chromatin           cytosol               ER              ERGIC
##                 41               370              273                 15
##                 GA          lysosome    mitochondrion            nucleus
##                 23                32              362                224
##         peroxisome                PM       proteasome ribosome/complexes
##                 15                44               33                 64



Next, we use the lapply function to loop over each item in our data list (i.e., each replicate of each condition) and add the mrk markers using the addMarkers function. Importantly, the same marker list must be added to all replicates of all conditions for input to BANDLE.

## Add markers to each replicate of each condition (stored in data list)
data <- lapply(data, addMarkers, mrk)



Next, we use the function commonFeatureNames to extract proteins that are common across all replicates of both conditions. This function has a nice side effect which is that it also wraps the data into an MSnSetList, the format required for input into bandle.


## Extract common proteins across all 6 samples
input_bandle <- commonFeatureNames(data)


## 5700 features in common



### Settings priors

As mentioned above, BANDLE is inspired by the TAGM-MCMC model and uses Markov-Chain Monte-Carlo (MCMC) to sample the posterior distribution of parameters and latent variables from which statistics of interest can be computed. We will use the bandle function to apply this fully Bayesian algorithm to our data. However, before we can run bandle we must first define some priors. In Bayesian statistics, priors refer to estimates of the model parameters prior to observing any data (i.e., based on prior knowledge). Users who are not familiar with these concepts may find the interpretation of priors challenging. For a more in-depth discussion of Baysian statistics and the BANDLE model specifically users are directed to. ^48, 10^

**Constructing a penalised complexity prior matrix** ( pcPrior): First, we define a matrix of penalised complexity priors for the model hyperparameters. We will then use these priors to fit non-parametric regression functions, termed Gaussian Processes (GPs), to the marker profiles. In general, the default priors on the hyperparameters (see ?fitGP) work well for correlation profiling data with 10 or less channels (as tested in Crook et al. ^10^). The default priors on the hyperparameters are hyppar = matrix(c(0.5, 3, 100), nrow = 1) (see ?bandle for documentation). Different priors can also be constructed and tested. For example, here, we found that matrix(c(10, 60, 100) worked well.

The prior needs to form a K*3 matrix where K corresponds to the number of subcellular classes in the data, and the 3 columns are; (1) length-scale, (2) amplitude and (3) variance (see hyppar in ?fitGPmaternPC). Generally, (1) increasing the length-scale parameter increases the spread of the covariance i.e. the similarity between points, (2) increasing the amplitude parameter increases the maximum value of the covariance and lastly (3) decreasing the variance reduces the smoothness of the function to allow for local variations. Increasing these values, increases the shrinkage. For more details see the manuscript by Crook et al. ^10^ We strongly advise that users start with the recommended hyppar parameters and assess their suitability for their dataset by visually evaluating the fit of the GPs using the plotGPmatern function, as demonstrated below.

We begin by constructing a matrix of priors and saving these to an object called pc_prior.

## Extract subcellular classes
mrkCl <- getMarkerClasses(input_bandle[[1]], fcol = "markers")

## Construct a pc_prior
set.seed(1)
K <- length(mrkCl) # K = number of subcellular classes
pc_prior <- matrix(NA, ncol = 3, K) # Initiate empty matrix
pc_prior[seq.int(1:K), ] <- matrix(rep(c(10, 60, 100), each = K), ncol = 3)



**Fitting Gaussian Processes to the data** ( gpParams): Now that we have defined our penalised complexity priors we use these to fit GPs to the datasets using the fitGPmaternPC function. We pass the pc_prior object to the hyppar argument and use set.seed to ensure reproducibility. These GPs are saved to an object called gpParams. Once the GPs have been fit we can visually assess their suitability using the plotGPmatern function.

## Fit GP priors to the data - each sample separately
set.seed(1)
gpParams <- lapply(input_bandle,
                   function(x) fitGPmaternPC(x, hyppar = pc_prior))

## Using plotGPmatern to overlay the predictives
## For demonstration purposes plot just the first
par(mfrow = c(4, 3))
plotGPmatern(input_bandle[[1]], gpParams[[1]])



**Figure 29.**
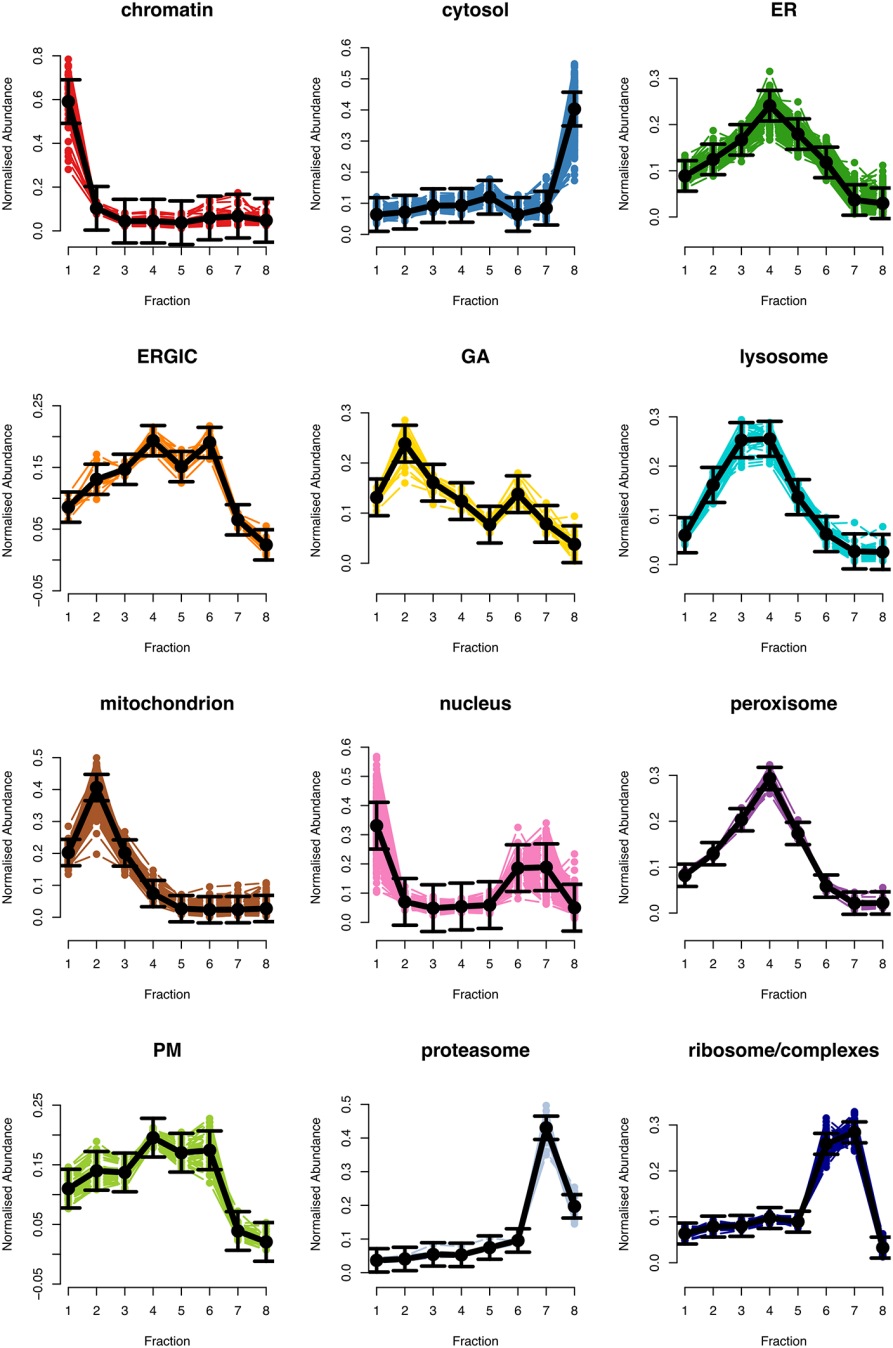
Profiles of the marker proteins with the posterior predictives overlayed.

We see that the fit looks sensible and the predictives (shown in black) overlay nicely with the marker profiles of the unstimulated replicate 1 data ( Figure 29). Users should assess the fit of GPs for each replicate of each condition by altering the above code to extract each item in the input_bandle and gpParams lists (i.e., changing the indices inside of the [[]]). The GPs should fit all datasets. If the posterior predictives do not fit the pattern of marker proteins, users should test alternative priors.

**Setting up the Dirichlet prior matrix** ( dirPrior): The next step is to set up the matrix Dirichlet prior on the mixing weights. These weights are defined across datasets so are slightly different to mixture weights in usual mixture models. The (i, j)th entry is the prior probability that a protein localises to organelle i in the control and j in the treatment. This mean that off-diagonal terms (terms in which the control and treatment organelles differ i.e., differential localisation) have a different interpretation to diagonal terms (terms in which the control and treatment organelles are the same i.e., no differential localisation). Since we expect differential localisation to be rare, off-diagonal terms should be small.

## Set up Dirichlet prior matrix
dirPrior <- diag(rep(1, K)) + matrix(0.0005, nrow = K, ncol = K)

## Determine prior probability of >15 differential localisation events
predDirPrior <- prior_pred_dir(object = input_bandle[[1]],
                               dirPrior = dirPrior,
                               q = 15)



**Figure 30.**
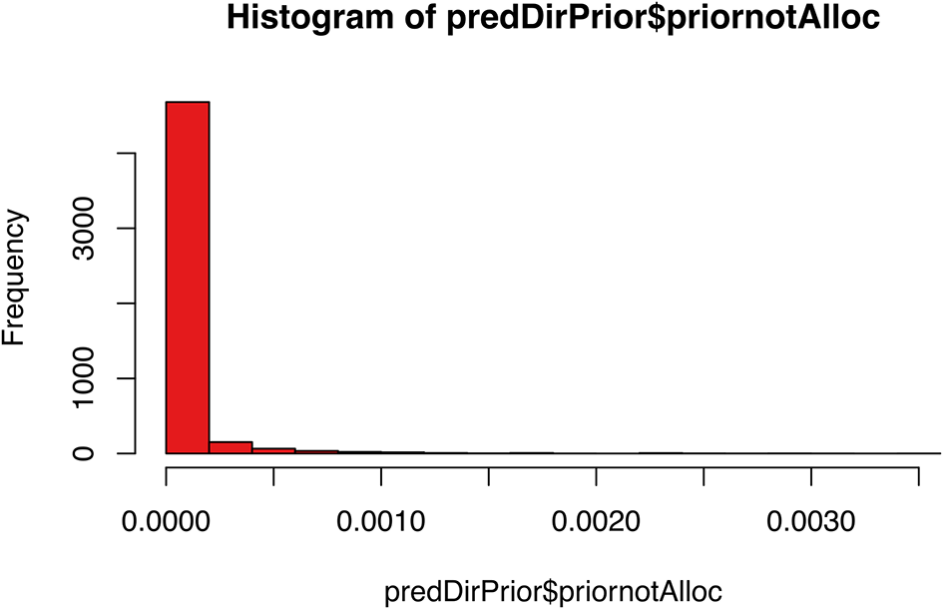
Histogram of the prior probability that proteins are allocated to different components (subcellular locations) between datasets.

We can plot a histogram ( Figure 30) of the prior probability that proteins are allocated to two different subcellular compartments between conditions by accessing predDirPrior$priornotAlloc, as demonstrated below.

## Plot histogram of differential localisation probability based on priors
hist(predDirPrior$priornotAlloc, col = getStockcol()[1])



We see that the prior probability that proteins are allocated to different components between datasets concentrates around 0. This is what we expect since only a small proportion of proteins will be differentially localised whilst the majority remain in their original localisation.

### Running BANDLE

Now that we have computed our pcPrior, gpParams and dirPrior we can run the main BANDLE algorithm. The bandle function implements the BANDLE model using MCMC inference. As per the TAGM-MCMC algorithm we need to specify the number of iterations, chains, burnin and thin parameters, along with our priors. We refer readers to part 2 for the details regarding these parameters. The bandle function is computationally heavy and running the BANDLE algorithm for 10,000 iterations and 5 chains with the below parameters took approximately 3 hours on a local machine using 12 cores. If users wish to get a feel for how to apply the method before doing a full run we suggest using just 20 iterations with 5 burnin and thinning every other iteration (as per, for example, the bandle vignette). This will not result in a converged model and we do not suggest doing this for real-life applications but it gives users a feel for how to run the method.

Before we run BANDLE we first subset our data into two objects called cond1 and cond2, each a list of replicates in a given condition.

## Create lists of replicate MSnSets per condition
cond1 <- list(input_bandle[[1]], input_bandle[[2]], input_bandle[[3]])
cond2 <- list(input_bandle[[4]], input_bandle[[5]], input_bandle[[6]])



We pass these objects to the bandle function along with our previously optimised parameters ( gpParams, dirPrior, pc_prior). We run BANDLE with 5 parallel MCMC chains, each of 10,000 iterations with burnin of 5,000 (the number of samples to be discarded from the beginning of the chain) and a thinning frequency of 20 iterations. Since it takes several hours to run the full model locally, we here display the code used and load the resulting pre-computed bandleres object.

## Run the BANDLE algorithm
bandleres <- bandle(objectCond1 = cond1,
                    objectCond2 = cond2,
                    numIter = 10000L,
                    burnin = 5000L,
                    thin = 20L,
                    gpParams = gpParams,
                    numChains = 5,
                    dirPrior = dirPrior,
                    pcPrior = pc_prior,
                    seed = 1,
                    BPPARAM = MulticoreParam())


## Load pre-computed output generated with code chunk above
load("bandleres.rda", verbose = TRUE)


## Loading objects:
##   bandleres
##   cond1
##   cond2



This pre-computed bandleres object can be downloaded from Zenodo at doi: 10.5281/zenodo.15100485.

### Assessing convergence of the BANDLE algorithm

As we did for the TAGM-MCMC method in section 2, we must assess our MCMC chains of the BANDLE model for convergence. This is an important step to ensure that we have generated reasonable estimates on which we are going to base our inference. The two main functions we can use to help us assess convergence are (1) calculateGelman which calculates the Gelman diagnostics for all pairwise chain combinations and (2) plotOutliers which generates trace and density plots for all chains.

Let’s start with the Gelman which allows us to compare the inter and intra chain variances. If the chains have converged the ratio of these quantities should be close to one.

## Calculate Gelman diagnostic for all pairs of chains
bandleres %>%
  calculateGelman()


## $Condition1
##            comb_12  comb_13  comb_14   comb_15  comb_23  comb_24  comb_25
## Point_Est 1.004939 1.014478 1.005598 0.9982596 1.007572 1.005648 1.003474
## Upper_CI  1.022159 1.071408 1.030113 0.9982596 1.017721 1.005950 1.020222
##             comb_34  comb_35  comb_45
## Point_Est 0.9999416 1.015382 1.006845
## Upper_CI  1.0076398 1.071347 1.030943
##
## $Condition2
##            comb_12  comb_13  comb_14  comb_15   comb_23   comb_24   comb_25
## Point_Est 1.012134 1.012824 1.009356 1.007928 0.9990051 0.9988274 0.9990425
## Upper_CI  1.056042 1.046211 1.050258 1.030302 0.9995774 0.9989415 1.0023945
##            comb_34   comb_35   comb_45
## Point_Est 1.001355 0.9984959  1.000401
## Upper_CI  1.001551 0.9995919  1.002772



**Figure 31.**
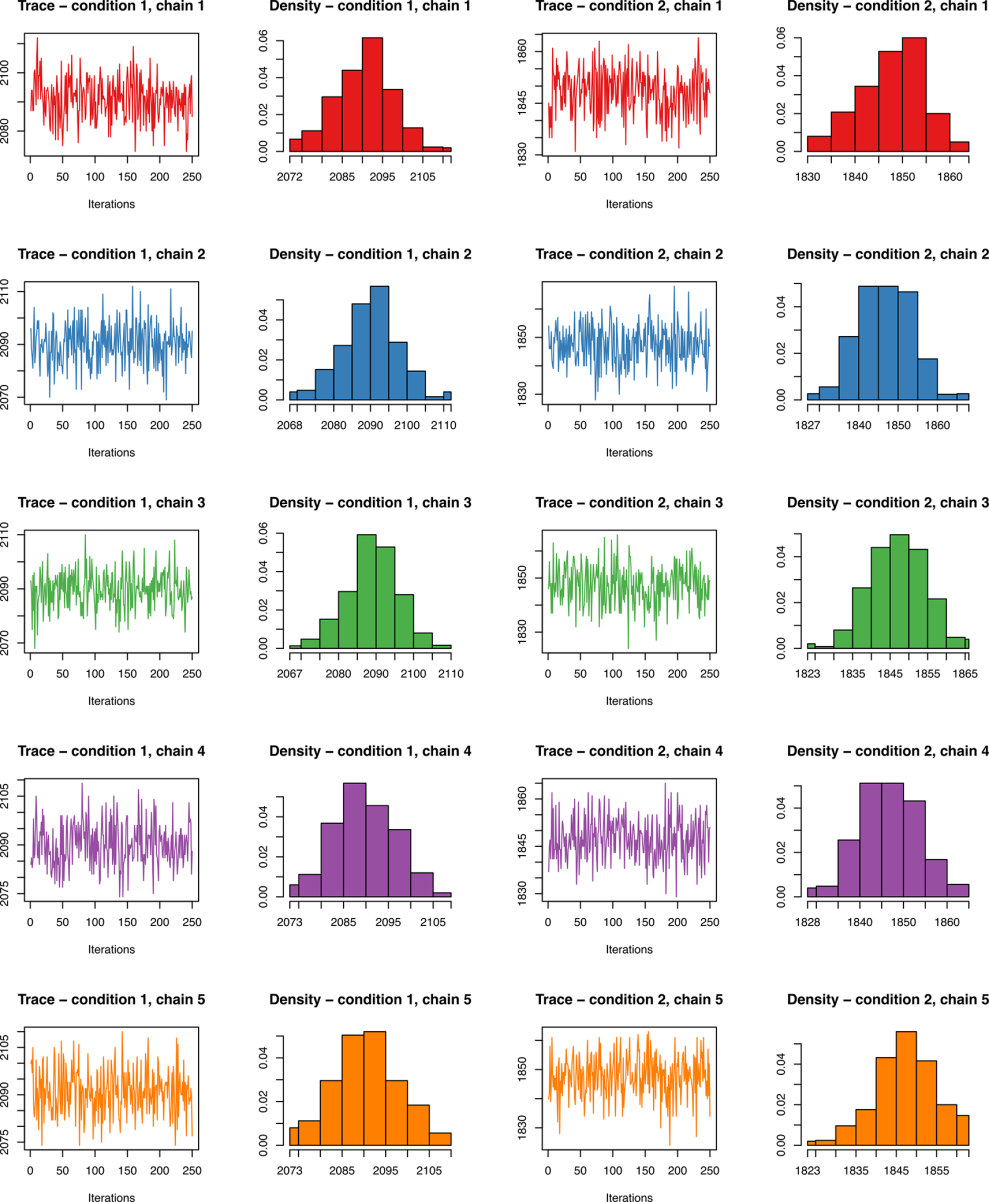
Trace and density plots for the five MCMC chains, for each replicated condition.

We see that the point estimate Gelman diagnostics are less than 1.2 which is an indicator of chain convergence. The upper confidence intervals are also less than 1.2 for all pairs of chains. Let’s now generate trace and density plots ( Figure 31) to visually assess the number of outliers across the chains.

## Generate trace and density plots per chain
bandleres %>%
  plotOutliers()

Trace plots are subjective but can used to help visually assess the sample path of the chains. Textbooks on Bayesian inference often tell us that a good trace plot should look like a “hairy” or “fuzzy caterpillar”. Users will note that the total number of iterations across the x-axis of the trace plots is 250. We indeed have estimates for 250 points. We recall we set the numIter to 10,000 and burnin to 5,000, thus leaving 5,000 remaining iterations to draw our samples from. As we thinned every 20, this resulted in keeping 250 points across the data. The y-axis shows the number of outliers for each of these iterations. The plots show reasonable sampling of the data space. The density plots show the number of outliers for each iteration expressed as a probability density. For convergence we expect to see a normally distributed plot centered around roughly the same number of outliers in each chain. This is indeed what we see for this BANDLE run. We can conclude that we have reached a stationary distribution and proceed with inference.

If the number of outliers was wildly different for one of the chains, or if the trace plot has a long period of burn-in (the beginning of the trace looks very different from the rest of the plot), or high serial correlation (the chain is very slow at exploring the sample space) we may wish to discard these chains and, depending on how many chains remain, run more chains.

### Removing chains

The MCMC results from BANDLE look reasonable and for this use-case we do not need to remove any chains. If, however, we had some chains that looked as though they did not converge we could remove them and base our inference on the other “good” chains. We suggest taking forward a minimum of 3 chains for inference. Here we have run 5 chains. For demonstration purposes, let’s remove 2 of the chains. In practice, we would keep all of these chains as there are no obvious problems. We also note, that if a chain in bad in one condition, it must be discarded in the second condition. Chains are considered in pairs. This is done automatically when we remove chains, as demonstrated below.

## Check current number of chains
bandleres


## Object of class "bandleParams"
## Method: bandle
## Number of chains: 5



Let’s remove chain 2 and chain 3 for demonstration. To remove chains we subset the bandleres object as we would a standard list in R passing the indices of the chains to be removed. We generate a new object called bandleres_converged.

## Remove chains 2 and 3
bandleres_converged <- bandleres[-c(2, 3)]



### Processing the output of BANDLE

The BANDLE method generates probabilistic predictions for (1) protein subcellular localisation and (2) the differential localisation of proteins. In this section we will use the bandleProcess and bandlePredict functions to extract our estimates and perform protein subcellular localisation prediction.

### Running the bandleProcess and bandlePredict functions

Satisfied with the BANDLE parameters we have generated we next pass these to the bandleProcess function to populate the object with probability estimates for the subcellular localisation across organelles and differential localisation estimates for all proteins.

## Process BANDLE results
params <- bandleProcess(bandleres_converged)



The resultant object is of class bandleParams.

## Check class of params
params %>%
  class()


## [1] "bandleParams"
## attr(,"package")
## [1] "bandle"



Using the bandlePredict function we now append these results to the first dataset in the list of MSnSets, for each condition i.e. cond1 and cond2. The results are the same across all replicates of a condition, hence they are only appended to the first replicate.

## Append BANDLE results to first entry in list of each condition
res <- bandlePredict(objectCond1 = cond1,
                     objectCond2 = cond2,
                     params = params,
                     fcol = "markers")

## Extract results for each condition
res_unstim <- res[[1]]
res_xray <- res[[2]]



We can verify that the results have been appended by checking the column names of the fData.

## Check column names of fData
res_unstim[[1]] %>%
  fvarLabels()


res_xray[[1]] %>%
  fvarLabels()


##  [1] "Checked"                          "Tags"
##  [3] "Confidence"                       "Identifying.Node.Type"
##  [5] "Search.ID"                        "PSM.Ambiguity"
##  [7] "Contaminant"                      "Number.of.Proteins"
##  [9] "Master.Protein.Accessions"        "Master.Protein.Descriptions"
## [11] "Protein.Accessions"               "Protein.Descriptions"
## [13] "Delta.Cn"                         "Rank"
## [15] "Search.Engine.Rank"               "Concatenated.Rank"
## [17] "Ions.Matched"                     "Matched.Ions"
## [19] "Total.Ions"                       "Activation.Type"
## [21] "MS.Order"                         "Quan.Info"
## [23] "Number.of.Protein.Groups"         "replicate"
## [25] "Protein.Confidence"               ".n"
## [27] "markers"                          "bandle.allocation"
## [29] "bandle.probability"               "bandle.probability.lowerquantile"
## [31] "bandle.probability.upperquantile" "bandle.mean.shannon"
## [33] "bandle.differential.localisation" "bandle.outlier"
## [35] "bandle.joint"



We see the first replicate has 35 columns and the results have been appended to the columns 28:35 in both the control and treated conditions.

The BANDLE results are shown in the columns:

• bandle.allocation which contains the the localisation predictions to one of the subcellular classes that appear in the training data.
• bandle.probability is the allocation probability, corresponding to the mean of the distribution probability.
• bandle.probability.lowerquantile and bandle.probability.upperquantile are the upper and lower quantiles of the allocation probability distribution.
• bandle.mean.shannon is the Shannon entropy, measuring the uncertainty in the allocations (a high value representing high uncertainty; the highest value is the natural logarithm of the number of classes).
• bandle.differential.localisation is the differential localisation probability.
• bandle.outlier is the probability of being an outlier. A high value indicates that the protein is unlikely to belong to any annotated class (and is hence considered an outlier).
• bandle.joint which is the full joint probability distribution across all subcellular classes.

In the subsequent code chunks we will work with the first MSnSet in each condition as these are where the BANDLE results are located for each condition. These results are the the combined predictions made from all three replicates.

res_unstim_rep1 <- res_unstim[[1]]
res_xray_rep1 <- res_xray[[1]]



### Thresholding

As mentioned in part 2 of this workflow, it is common practice to threshold protein localisation allocation results based on the posterior probability. Proteins that do not meet the threshold are not assigned to a subcellular location and are instead left unlabelled (here we use the terminology “unknown” for consistency with the pRoloc package). It is important not to force proteins to allocate to one of the niches defined in the training data if they have low probability to reside there. We wish to allow for greater subcellular diversity and explore the possibility that proteins are localised to multiple locations. This is essentially captured by leaving a protein “unlabelled” or “unknown”. We can also extract the “unknown” proteins with high uncertainty and examine their distribution over all organelles (see bandle.joint).

To extract proteins localised to one location with high confidence, as we did for the TAGM classifiers, we make use of the outlier probability to filter out predictions that lie on the periphery of the classification boundaries. Users could filter on the posterior probability only rather than both the posterior and outlier probabilities if they wish to be less strict with their organelle definition.

In the following code chunk we create a new column in the fData called bandle.probability.overall which adjusts our classification predictions for outliers. We multiple the bandle.probability with 1 - bandle.outlier to obtain a probability distribution that is adjusted for protein outliers, as we did previously when using the TAGM models. We add this information to each of the MSnSets.

## Store allocation and outlier probabilities for each condition
bandle_unstim_prob <- fData(res_unstim_rep1)$bandle.probability
bandle_xray_prob <- fData(res_xray_rep1)$bandle.probability

bandle_unstim_out <- 1 - fData(res_unstim_rep1)$bandle.outlier
bandle_xray_out <- 1 - fData(res_xray_rep1)$bandle.outlier

## Create new column containing overall bandle probabilities per condition
fData(res_unstim_rep1)$bandle.probability.overall <-
  bandle_unstim_prob * bandle_unstim_out
fData(res_xray_rep1)$bandle.probability.overall <-
  bandle_xray_prob * bandle_xray_out



To obtain classification results we threshold the data assigning proteins to subcellular compartments if they obtain a probability > 0.99 based on the bandle.probability.overall. As before, we append the thresholded results to the fData data using the getPredictions function.

## Threshold BANDLE localisation predictions
res_unstim_rep1 <- getPredictions(res_unstim_rep1,
                                  fcol = "bandle.allocation",
                                  scol = "bandle.probability.overall",
                                  mcol = "markers",
                                  t = .99)


## ans
##          chromatin           cytosol               ER              ERGIC
##                199               704              412                 99
##                 GA          lysosome    mitochondrion            nucleus
##                124                66              564                558
##         peroxisome                PM       proteasome ribosome/complexes
##                 29               337               50                209
##            unknown
##               2349


res_xray_rep1 <- getPredictions(res_xray_rep1,
                                fcol = "bandle.allocation",
                                scol = "bandle.probability.overall",
                                mcol = "markers",
                                t = .99)


## ans
##          chromatin           cytosol               ER              ERGIC
##                208               763              425                130
##                 GA          lysosome    mitochondrion            nucleus
##                137                71              561                580
##         peroxisome                PM       proteasome ribosome/complexes
##                 29               382               89                219
##            unknown
##               2106



A new column has been added to fData called bandle.allocation.pred.

## Check which columns are present in fData
res_unstim_rep1 %>%
  fvarLabels()


##  [1] "Checked"                          "Tags"
##  [3] "Confidence"                       "Identifying.Node.Type"
##  [5] "Search.ID"                        "PSM.Ambiguity"
##  [7] "Contaminant"                      "Number.of.Proteins"
##  [9] "Master.Protein.Accessions"        "Master.Protein.Descriptions"
## [11] "Protein.Accessions"               "Protein.Descriptions"
## [13] "Delta.Cn"                         "Rank"
## [15] "Search.Engine.Rank"               "Concatenated.Rank"
## [17] "Ions.Matched"                     "Matched.Ions"
## [19] "Total.Ions"                       "Activation.Type"
## [21] "MS.Order"                         "Quan.Info"
## [23] "Number.of.Protein.Groups"         "replicate"
## [25] "Protein.Confidence"               ".n"
## [27] "markers""bandle.allocation"       "bandle.allocation"
## [29] "bandle.probability"               "bandle.probability.lowerquantile"
## [31] "bandle.probability.upperquantile" "bandle.mean.shannon"
## [33] "bandle.differential.localisation" "bandle.outlier"
## [35] "bandle.joint"                     "bandle.probability.overall"
## [37] "bandle.allocation.pred"



### Extracting protein localisation predictions from BANDLE

### Single locations

We can examine the distribution of allocations that have been assigned to a single location with high confidence. Before we do this, we subset the data to remove the marker proteins such that our data only contain proteins which received new localisation predictions.

## Extract BANDLE results minus marker proteins
res_no_mrk1 <- unknownMSnSet(res_unstim_rep1, fcol = "markers")
res_no_mrk2 <- unknownMSnSet(res_xray_rep1, fcol = "markers")



We can visualise the new assignments from BANDLE by plotting them on a barplot.

## Pull column containing BANDLE localisation predictions in each condition
alloc1 <- res_no_mrk1 %>%
   fData() %>%
   pull(bandle.allocation.pred)

alloc2 <- res_no_mrk2 %>%
   fData() %>%
   pull(bandle.allocation.pred)

## Plot barplot of BANDLE localisation predictions in each condition
par(mfrow = c(1, 2))
barplot(alloc1 %>% table,
        las = 2, main = "Predicted location: Unstimulated",
        ylab = "Number of proteins")
barplot(alloc2 %>% table(),
        las = 2, main = "Predicted location: X-ray",
        ylab = "Number of proteins")



**Figure 32.**
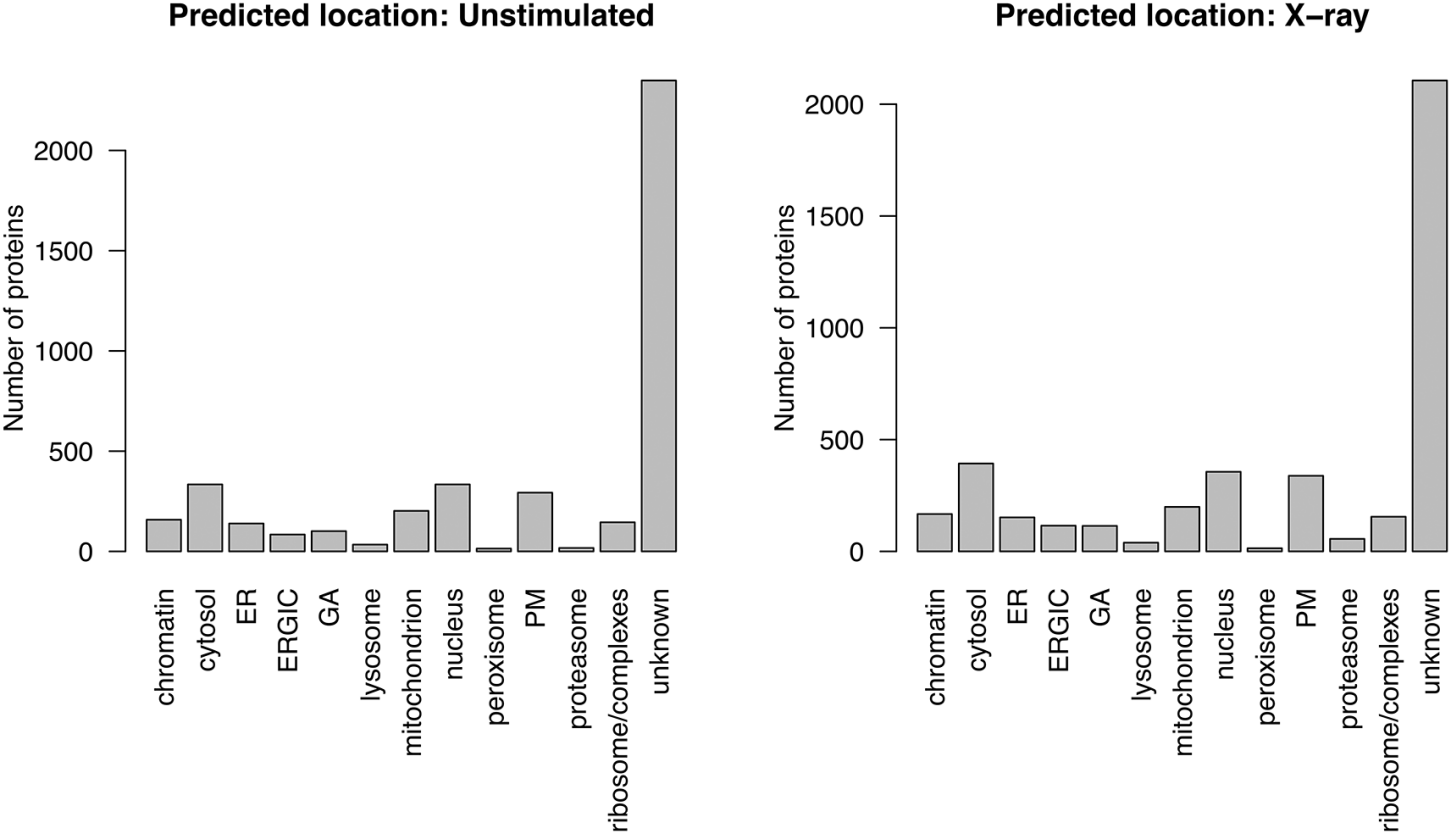
Barplots of the predicted locations of newly classified proteins from BANDLE analysis (markers omitted) for the unstimulated data (left), and x-ray stimulated data (right).

The barplots ( Figure 32) show us that for the use-case data BANDLE has allocated many new proteins to subcellular classes in our training data. Importantly, we also see that the classification distributions across compartments look similar between the two conditions. This is what we would expect given that only a small proportion of proteins will be differentially localised. If users observe a dramatic difference in the number of proteins classified to a given compartment between conditions, it is possible that this compartment was not equally resolved in both conditions. In such a scenario we advise users to plot the classifications (as demonstrated below) and re-assess whether further marker curation is required.

Interestingly, the barplots show that many proteins are left with no allocation after thresholding i.e. they are labelled as “unknown”. These are the proteins which exhibit uncertainty in their localisation allocations and thus potentially display mixed localisation. The associated posterior estimates are located in the bandle.probability column and we can construct a boxplot to examine these probabilities per compartment.

## Pull column containing BANDLE localisation posterior estimates
pe1 <- res_no_mrk1 %>%
  fData() %>%
  pull(bandle.probability)

pe2 <- res_no_mrk2 %>%
  fData() %>%
  pull(bandle.probability)

## Plot boxplots of BANDLE localisation posterior estimates
par(mfrow = c(1, 2))
boxplot(pe1 ~ alloc1, las = 2, main = "Posterior: Unstimulated",
        ylab = "Probability")
boxplot(pe2 ~ alloc2, las = 2, main = "Posterior: X-ray",
        ylab = "Probability")



**Figure 33.**
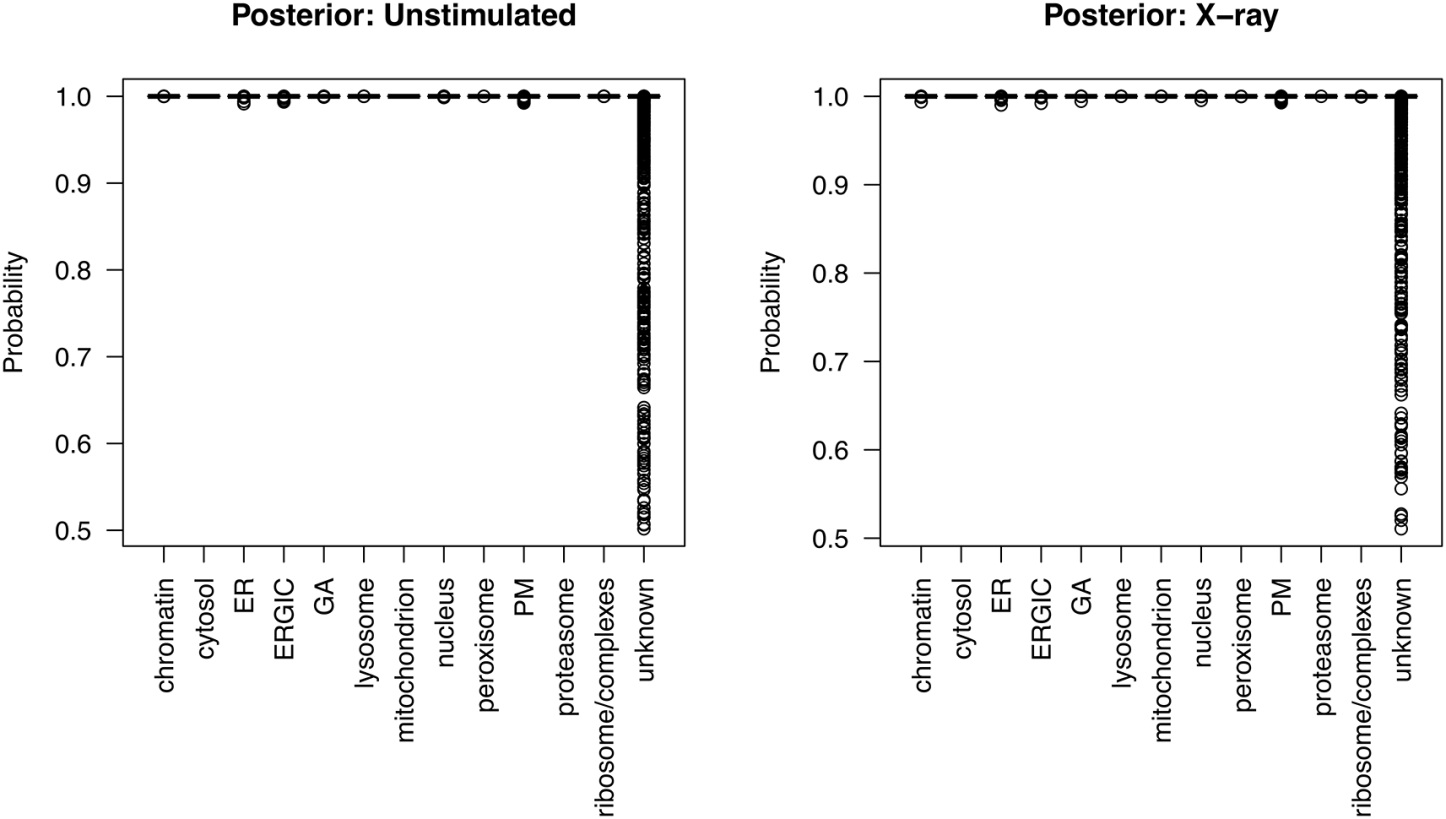
Boxplots displaying the BANDLE localisation posterior probabilities per protein across all subcellular classes.

As expected, we see that proteins in the “unknown” or “unlabelled” category have a wide range of different probabilities whilst those allocated confidently to a single localisation have high probabilities ( Figure 33). Proteins which are “unknown” but still have a high probability are likely to also have a high outlier probability or lie on the classification boundaries.

In order to plot the proteins allocated confidently to a single localisation we first need to populate all datasets with the prediction results. Currently the results are only appended to the first MSnSet of each condition. We then use the plot2D function, as demonstrated previously, to plot the results of BANDLE classification per condition per replicate ( Figure 34).

par(mfrow = c(4, 2))
for (i in seq_along(cond1)) {
  # append results
  fData(res_unstim[[i]])$bandle.allocation.pred <-
    fData(res_unstim_rep1)$bandle.allocation.pred
  fData(res_xray[[i]])$bandle.allocation.pred <-
    fData(res_xray_rep1)$bandle.allocation.pred

  # plot maps per replicate
  plot2D(res_unstim[[i]], fcol = "bandle.allocation.pred",
         main = paste0("Unstimulated: replicate", i))
  plot2D(res_xray[[i]], fcol = "bandle.allocation.pred",
         main = paste0("X-ray: replicate", i))
}

# Add a legend
addLegend(res_unstim[[i]], where = "other", cex = 1.4)

**Figure 34.**
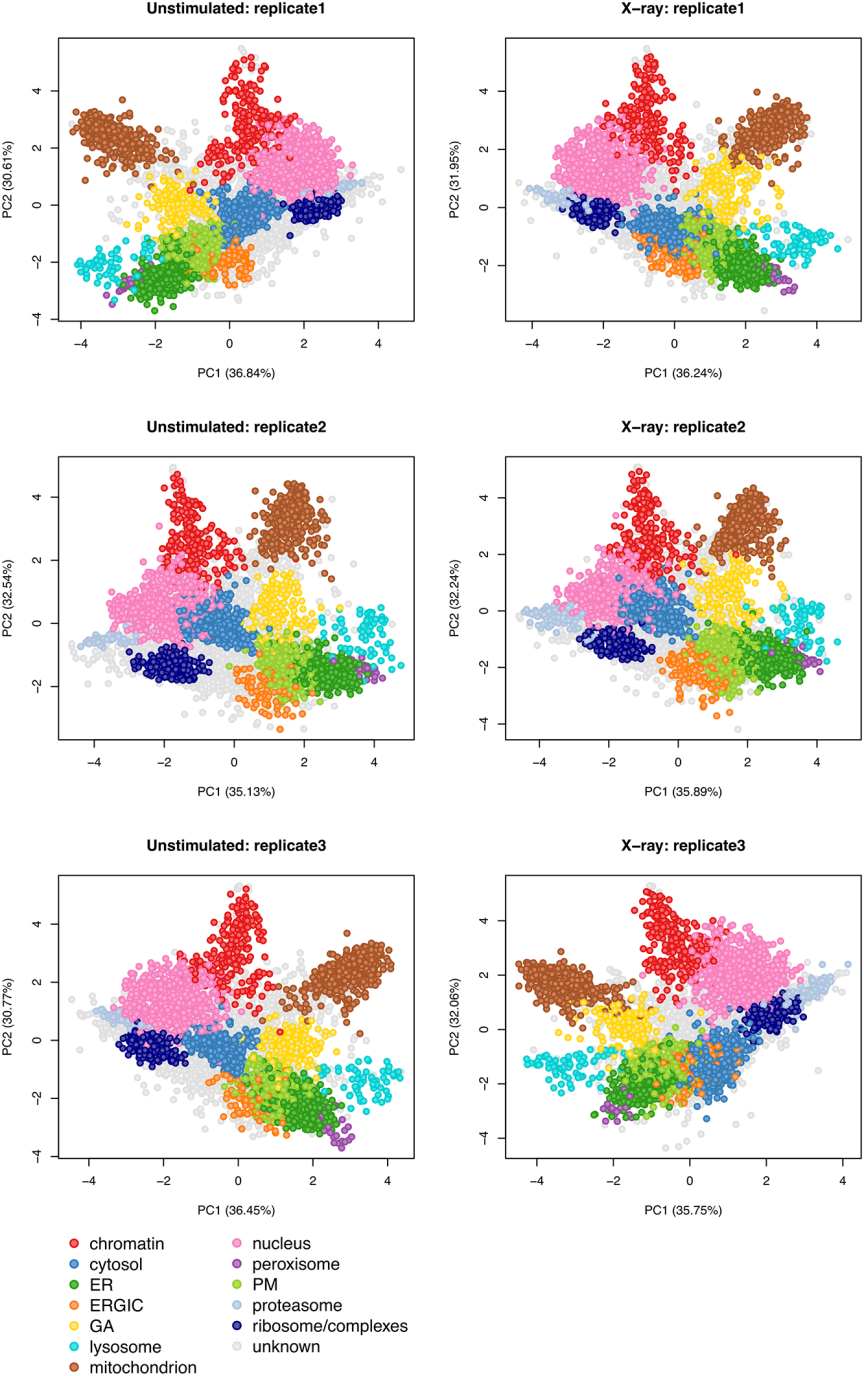
PCA plots displaying the subcellular localisation predictions from BANDLE classification, after thresholding for each replicated condition.

### Exploring proteins with uncertainty in their localisation

In addition to information about proteins classified to a single localisation included in the training data, BANDLE presents the opportunity to explore proteins with uncertain localisation, potentially representing multi-localised proteins.

First, we use the unknownMSnSet function to extract proteins which did not get a main location when we performed thresholding i.e. those labelled “unknown”.

## Extract "unknown" proteins after thresholding on each condition
res_mixed_cond1 <- unknownMSnSet(res_unstim_rep1, fcol = "bandle.allocation.pred")
res_mixed_cond2 <- unknownMSnSet(res_xray_rep1, fcol = "bandle.allocation.pred")

## Check how many proteins are "unknown" in each condition
res_mixed_cond1 %>% nrow()


## [1] 2349


res_mixed_cond2 %>% nrow()


## [1] 2106



We see that we have 2349 proteins that did not get assigned to one main location in the unstimulated data, and 2106 proteins that did not get assigned one main location in the treatment.

Let’s extract the names of these proteins so that we can investigate them further.

## Extract the protein accessions (feature names) of unknown proteins
fn1 <- featureNames(res_mixed_cond1)
fn2 <- featureNames(res_mixed_cond2)



**Figure 35.**
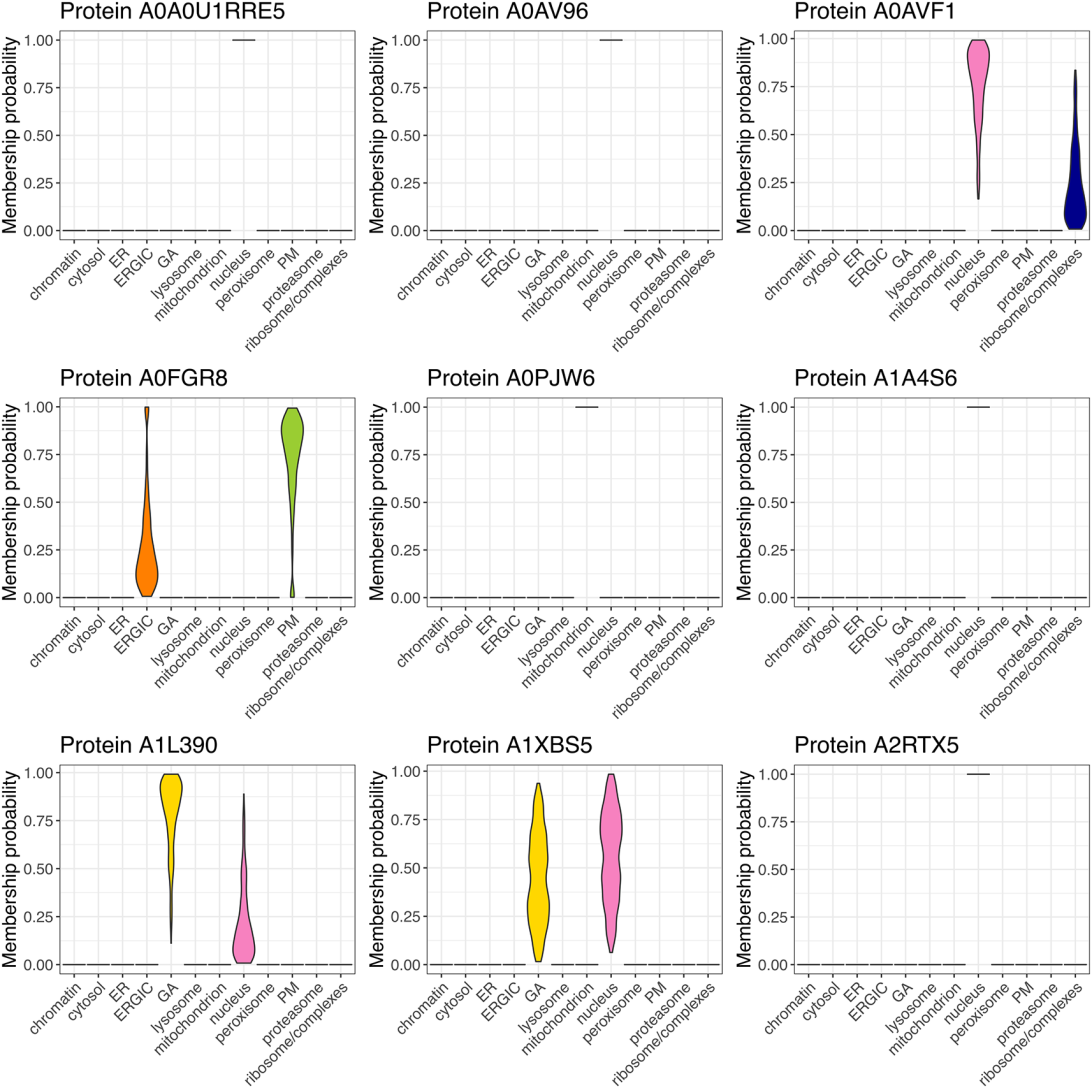
Violin plots displaying the BANDLE MCMC distribution of localisation probabilities across the subcellular niches in the data. Nine proteins are highlighted in the unstimulated data that did not get asisgned a main localisation after thresholding.

**Figure 36.**
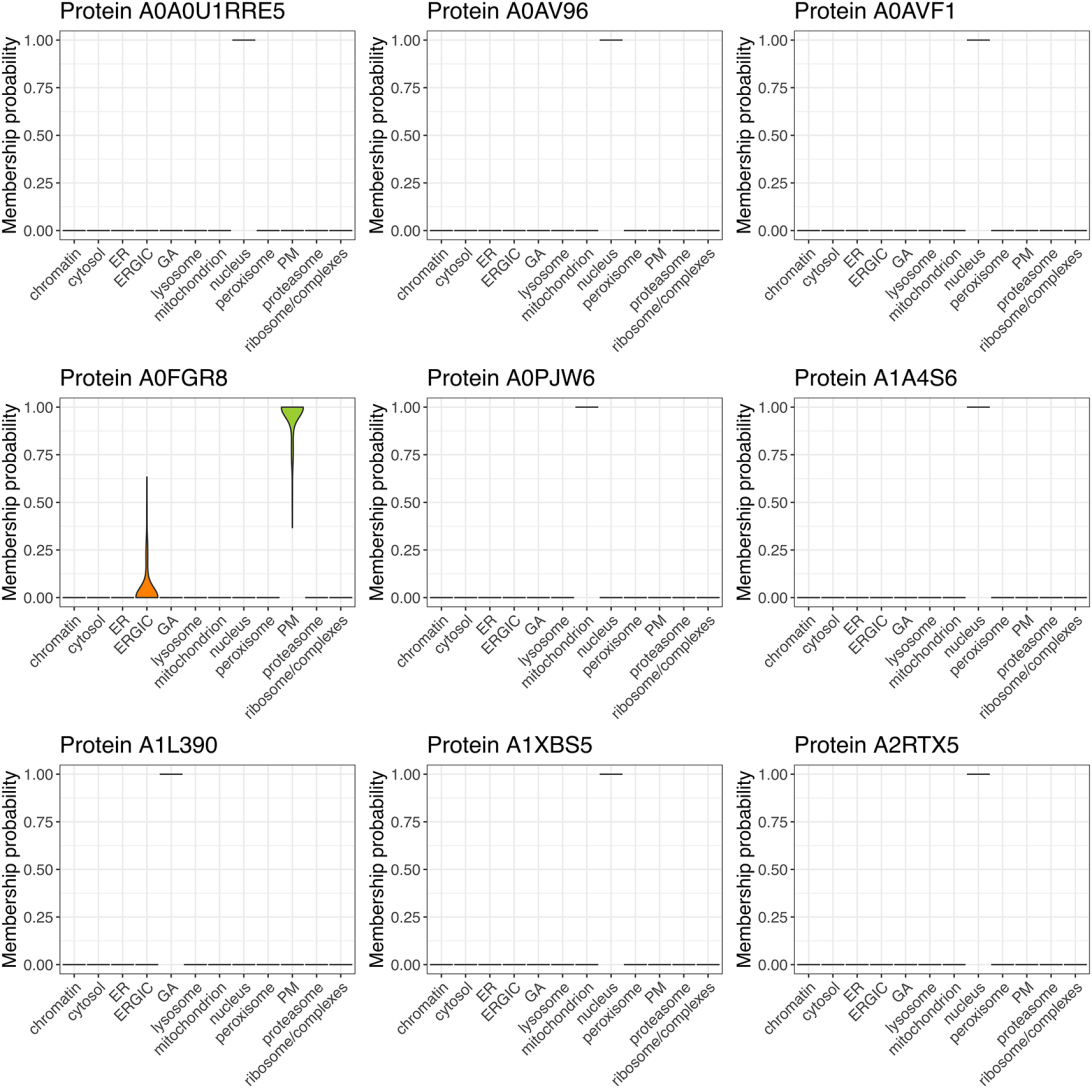
Violin plots displaying the BANDLE MCMC distribution of localisation probabilities across the subcellular niches in the data. Nine proteins are highlighted in the x-ray stimulated data that did not get asisgned a main localisation after thresholding.

The mcmc_plot_probs function can be used to generate violin plots of the localisation distributions of a protein of interest for the unstimulated ( Figure 35) and x-ray stimulated data ( Figure 36). In the code chunks below we show how to examine the first 9 proteins which did not get assigned a main subcellular localisation.

## Plot localisation distribution for first 9 unknown proteins in unstim
g <- vector("list", 9)
for (i in 1:9)
  g[[i]] <- mcmc_plot_probs(params, fn1[i], cond = 1)
do.call(grid.arrange, g)


## Plot localisation distribution for first 9 unknown proteins in xray
g <- vector("list", 9)
for (i in 1:9) g[[i]] <- mcmc_plot_probs(params, fn1[i], cond = 2)
do.call(grid.arrange, g)

From these plots we can see that some of the “unknown” proteins are potentially multilocalised as they have an uncertain location with membership probability split across two or more compartments. By contrast, other “unknown” proteins have a membership probability of near 1.0 for a single compartment. These proteins must be “unknown” due to a high probability of belonging to an outlier component, since we thresholded our classifications based on bandle.probability multiplied by 1 - bandle.outlier. Hence, proteins can be “unknown” due to 1) a low allocation probability ( bandle.probability) or high outlier probability ( bandle.outlier).

We can also get a summary of the full probability distribution by looking at the joint estimates stored in the bandle.joint slot of the MSnSet. This will give us one row per protein and one column per compartment, in which the values represent the membership probability ( bandle.probability) of that protein to that compartment.

## Extract full probability distribution for unknown proteins in unstim
res_mixed_cond1 %>%
  fData() %>%
  pull(bandle.joint) %>%
  head()


##              chromatin       cytosol            ER         ERGIC            GA
## A0A0U1RRE5 2.711979e-65  9.196591e-42 2.049920e-239 8.798116e-241 6.662549e-138
## A0AV96     1.629262e-56 1.343584e-137 1.105275e-116  2.987566e-51  4.716413e-67
## A0AVF1     5.380888e-66 2.840017e-100 2.439553e-212 2.355519e-201 1.498431e-119
## A0FGR8    5.775737e-77 3.214817e-131  2.279897e-17  2.636580e-01  1.295475e-32
## A0PJW6    6.779739e-45 6.950206e-215 6.916067e-285  0.000000e+00  4.191143e-84
## A1A4S6    2.352237e-49 7.327521e-110 9.709287e-134 3.467977e-138  1.479035e-41
##                 lysosome mitochondrion      nucleus   peroxisome            PM
## A0A0U1RRE5 2.531300e-314   0.000000e+00 1.000000e+00 0.000000e+00 8.073240e-175
## A0AV96      6.864402e-207 3.445242e-263 1.000000e+00 0.000000e+00  1.533369e-61
## A0AVF1     4.463920e-288 1.158345e-317 7.603868e-01 0.000000e+00 3.745508e-149
## A0FGR8       2.507399e-87 1.342191e-220 2.092688e-30 1.497077e-150  7.363420e-01
## A0PJW6     6.434494e-207  1.000000e+00 3.026051e-61 0.000000e+00 3.823400e-266
## A1A4S6     7.727091e-185 5.788136e-179 1.000000e+00  0.000000e+00  2.493783e-83
##               proteasome ribosome/complexes
## A0A0U1RRE5  7.028590e-86       4.866538e-99
## A0AV96     1.873003e-320       3.992689e-79
## A0AVF1      2.937760e-76       2.396132e-01
## A0FGR8      0.000000e+00      5.787601e-221
## A0PJW6      0.000000e+00       0.000000e+00
## A1A4S6     1.973827e-177      8.953622e-102



**Figure 37.**
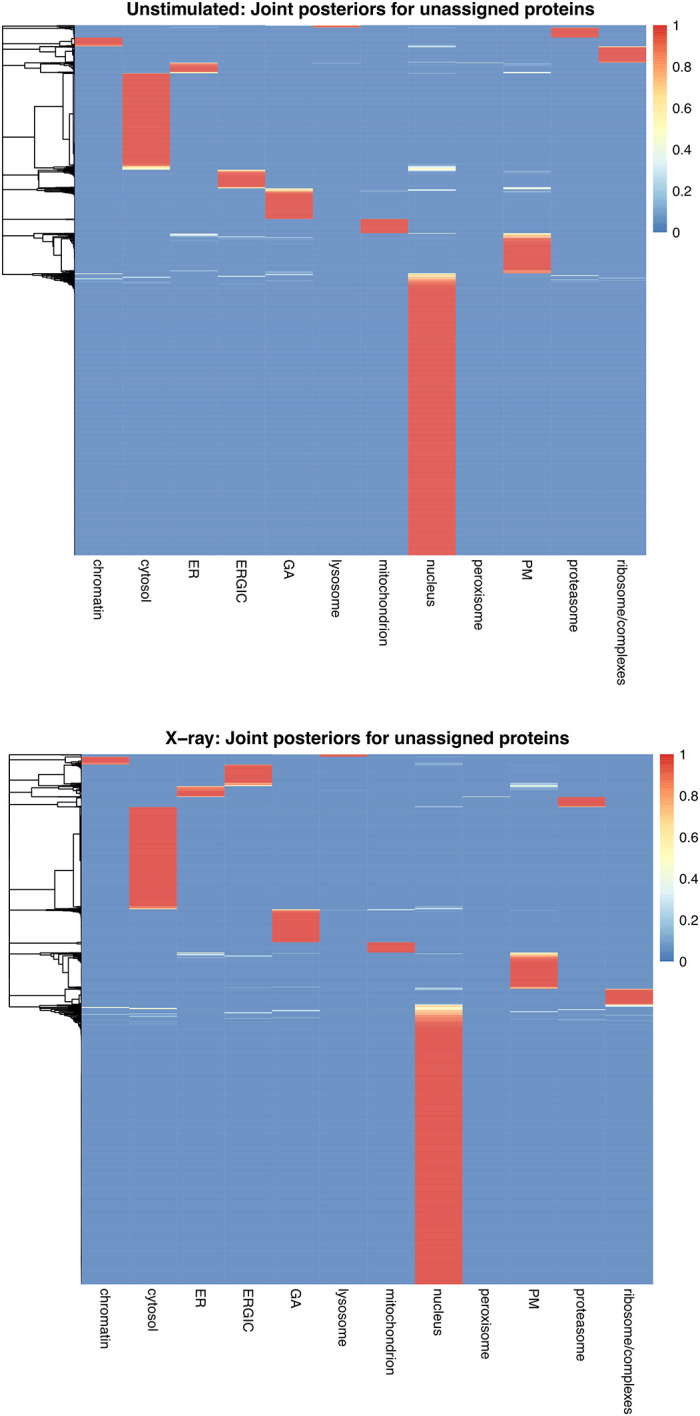
The joint posterior probability distributions for the unstimulated (top) and x-ray stimulated (bottom) data. The rows in each heatmap represent the proteins and the colour shows their joint posterior membership across compartments.

We can visualise the joint posteriors for the full set of unassigned proteins on a heatmap ( Figure 37).

## Visualise full probability distribution for unknown proteins in unstim
pheatmap(mat = fData(res_mixed_cond1)$bandle.joint,
         cluster_cols = FALSE,
         show_rownames = FALSE,
         main = "Unstimulated: Joint posteriors for unassigned proteins")

## Visualise full probability distribution for unknown proteins in xray
pheatmap(mat = fData(res_mixed_cond2)$bandle.joint,
         cluster_cols = FALSE,
         show_rownames = FALSE,
         main = "X-ray: Joint posteriors for unassigned proteins")

As observed in the violin plots above, we see that some of the “unassigned/unlabelled” proteins have low (blue) posterior membership probability across most compartments with a higher probability (white/red) across two or three compartments, whilst other proteins have a low membership probability (blue) in all except one compartment (red). As discussed above, we can assume that if the membership probability was high, the outlier probability was also high or the protein would have been allocated to a compartment at the thresholding stage. This scenario is particularly common for the nucleus and cytosol.

### Differential localisation results

As previously mentioned, the term “differentially localised” is used to refer to proteins which are assigned to different subcellular localisations between two conditions. For the majority of users this is the main output they are keen to extract using the BANDLE method. Whilst it would be possible to simply compare the lists of protein localisation allocations between conditions to identify those with different allocations, this qualitative approach does not provide any quantitative measure of confidence in the differential localisation. Instead, BANDLE provides a differential localisation probability which can be found in either (1) the bandle.differential.localisation column of the MSnSet that we generated following prediction, or (2) obtained directly from the bandleParams object using the diffLocalisationProb function. The latter is useful for users who are only interested in running BANDLE for obtaining differential localisation information and not in using BANDLE as a method for protein localisation prediction.

To obtain the differential localisation probability from a bandleParams object we can use the diffLocalisationProb function.

## Extract differential localisation predictions directly from params
dl <- diffLocalisationProb(params)

## Verify
dl %>%
  head()


## A0A0B4J2F0 A0A0U1RRE5 A0A0U1RRL7  A0AV96 A0AVF1 A0FGR8
##      0.000      0.000      0.000   0.000  0.268  0.236



**Figure 38.**
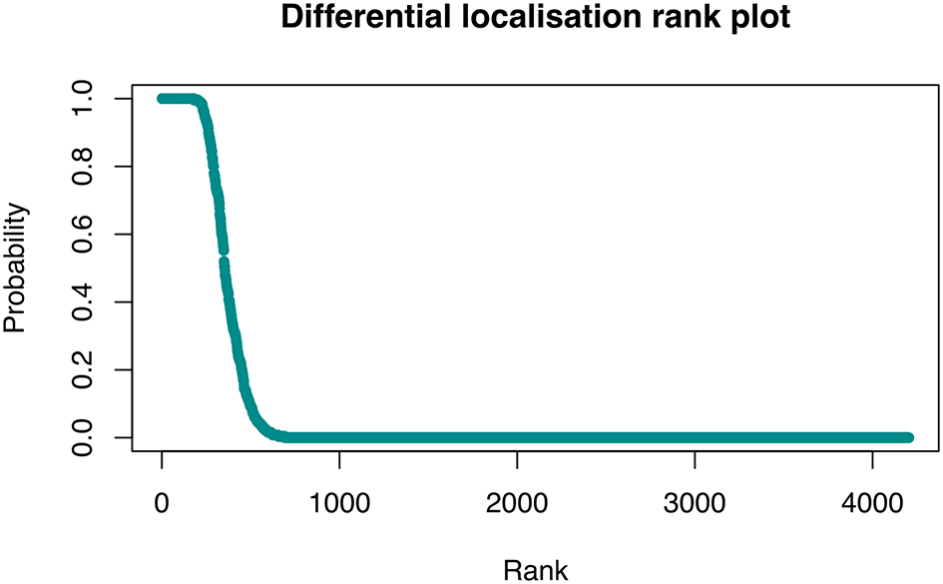
A rank plot showing the distribution of differential localisation probabilites per protein from BANDLE.

The differential localisation probability tells us which proteins are most likely to differentially localise. This can also be seen on a rank plot ( Figure 38).

## Rank plot of differential localisation probabilities output by bandle
plot(dl[order(dl, decreasing = TRUE)], cex = .7,
     col = "cyan4", pch = 19, ylab = "Probability",
     xlab = "Rank", main = "Differential localisation rank plot")

In-line with our expectations, the rank plot indicates that most proteins have a low probability of being differentially localised. We can set a threshold on the bandle.differential.localisation probability and see how many proteins pass this threshold. For example, let’s look at proteins with a differential localisation probability greater than 0.99.

## Extract proteins with differential localisation probability > 0.99
candidates <- which(dl > 0.99) %>%
  names()

## Check how many protein candidates pass this threshold
candidates %>%
  length()


## [1] 211



We have 211 proteins which meet this threshold and represent the most confident differentially localised candidates.

### Visualising differential localisation

There are several different ways we can visualise the movement of these proteins. The bandle package has functions to generate alluvial (also known as a Sankey diagram) and chord (also known as circos) plots to capture the flow of proteins between conditions.

First, we subset the data to visualise the proteins which have been classed as candidates i.e. those that have a differential localisation probability of greater than 0.99. We can set FDRs on the differential localisation using the EFDR function. We do not show this here but refer users to the package documentation by typing ?EFDR in the R console of RStudio.

## Extract candidates from both conditions
msnset_cands <- list(res_unstim_rep1[candidates, ],
                     res_xray_rep1[candidates, ])



If we examine this new object msnset_cands we see it is a list containing replicate 1 of the unstimulated data and replicate 1 of x-ray stimulated data respectively, subsetted for just the 211 proteins which are differential localisation candidates.

## Check the number of proteins in the object
lapply(msnset_cands, nrow)


## [[1]]
## [1] 211
##
## [[2]]
## [1] 211



**Figure 39.**
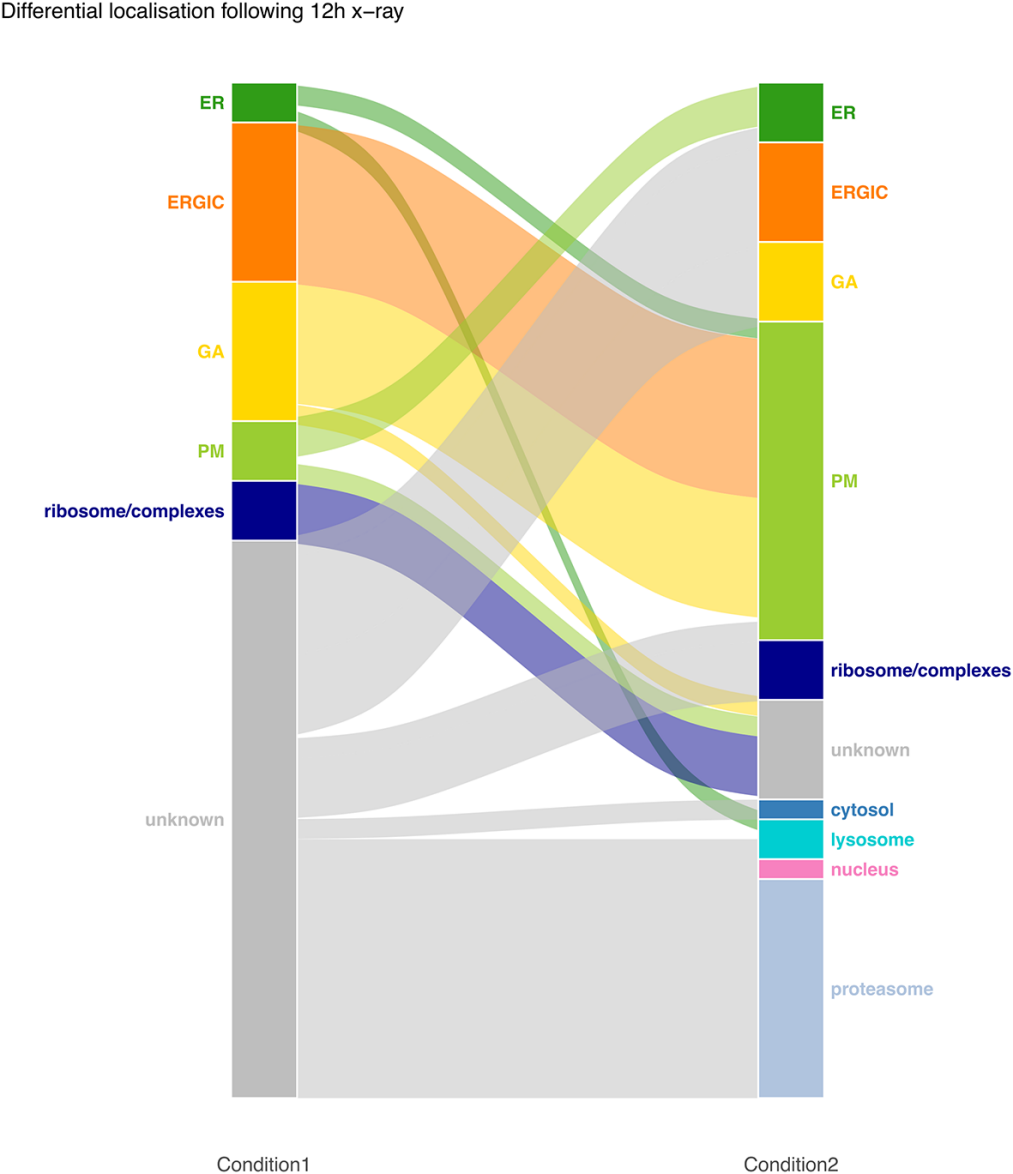
Alluvial plot showing the differential localisation patterns of proteins between subcellular niches between condition 1 (unstim) and condition 2 (xray).

Next, we use the plotTranslocations function to visualise which compartments the candidate proteins are differentially localised between. We first set a colour scheme for the subcellular classes and then pass the msnset_cands to plotTranslocations along with specifying the fcol containing the predicted subcellular localisations, here bandle.allocation.pred. The default plot that is generated by plotTranslocations is an alluvial plot and the function uses code from the ggalluvial R package ( Figure 39).

## Set the colours of the plot to match the organelles
cols <- c(getStockcol()[seq(mrkCl)], "grey")
names(cols) <- c(mrkCl, "unknown")

## Plot the flow of proteins
alluvial <- plotTranslocations(params = msnset_cands,
                               fcol = "bandle.allocation.pred",
                               col = cols)
alluvial + ggtitle("Differential localisation following 12h x-ray")

**Figure 40.**
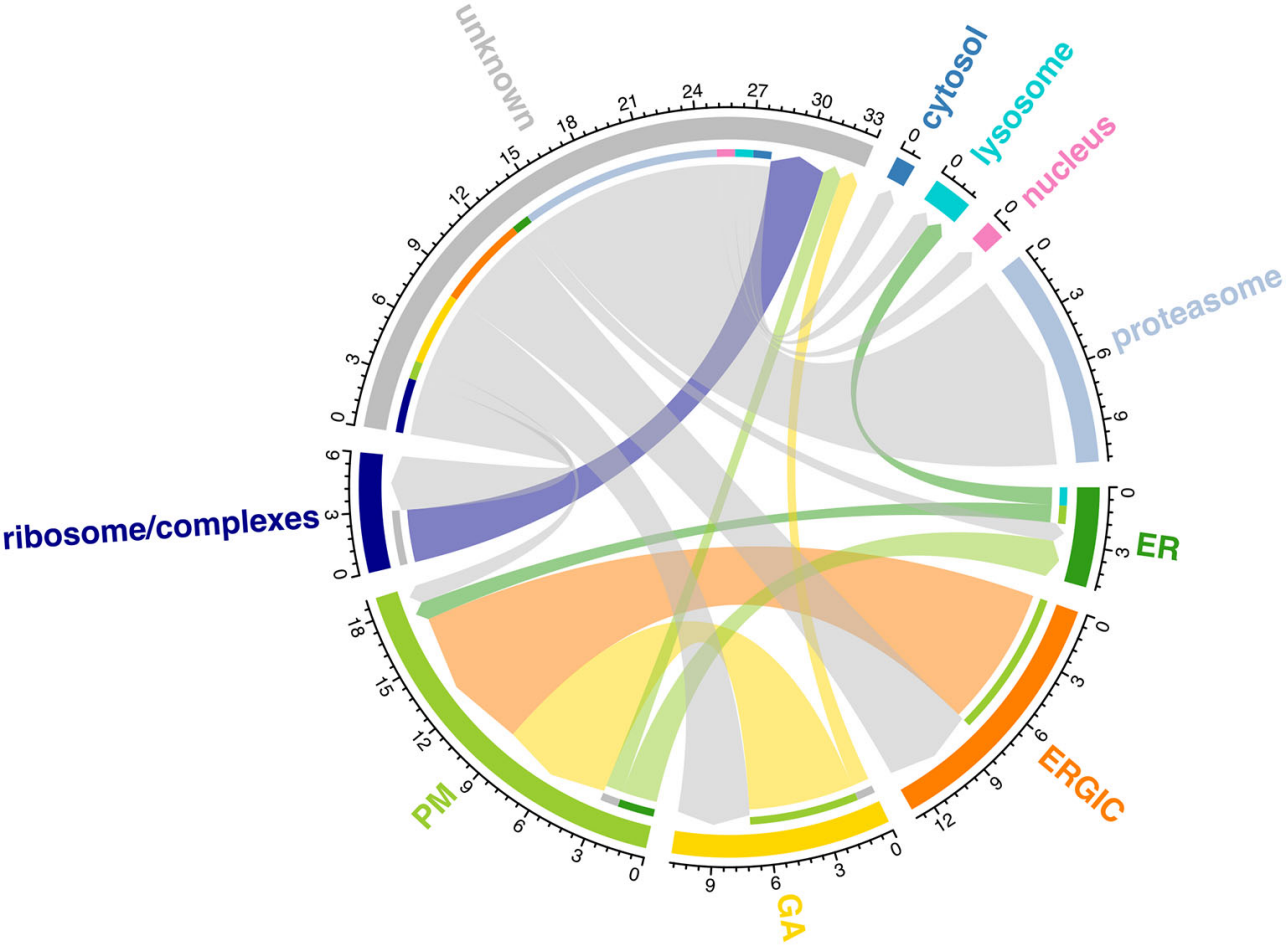
A chord plot showing the differential localisation patterns of proteins between subcellular niches.

We can also plot a chord diagram ( Figure 40) by passing type = "chord" to the plotTranslocations function. The function makes use of the circos package in R.

## Plot the flow of proteins
plotTranslocations(params = msnset_cands,
                   type = "chord",
                   fcol = "bandle.allocation.pred",
                   col = cols)

It is also possible to plot a summary table of translocating proteins using the plotTable function. We can pass our subsetted list of MSnSets for each condition to show a summary of just the proteins that meet the differential localisation threshold of 0.99, that we have set above.

## Plot a summary table
plotTable(params = msnset_cands,
          fcol = "bandle.allocation.pred")


## 211 features in common

## ------------------------------------------------
## If length(fcol) == 1 it is assumed that the
## same fcol is to be used for both datasets
## setting fcol = c(bandle.allocation.pred, bandle.allocation.pred)
## ----------------------------------------------

##            Condition1         Condition2 value
## 3                  ER                 PM     1
## 7                  ER           lysosome     1
## 12              ERGIC                 PM     8
## 21                 GA                 PM     6
## 23                 GA            unknown     1
## 28                 PM                 ER     2
## 32                 PM            unknown     1
## 41 ribosome/complexes            unknown     3
## 46            unknown                 ER     1
## 47            unknown              ERGIC     5
## 48            unknown                 GA     4
## 49            unknown                 PM     1
## 50            unknown ribosome/complexes     3
## 51            unknown            cytosol     1
## 52            unknown           lysosome     1
## 53            unknown            nucleus     1
## 54            unknown         proteasome    11



We can also plot a summary of all differential localisations by passing the whole dataset.

msnset_all <- list(res_unstim_rep1, res_xray_rep1)

## Plot a summary table
plotTable(params = msnset_all,
          fcol = "bandle.allocation.pred")


## 5700 features in common

## ------------------------------------------------
## If length(fcol) == 1 it is assumed that the
## same fcol is to be used for both datasets
## setting fcol = c(bandle.allocation.pred, bandle.allocation.pred)
## ----------------------------------------------

##             Condition1         Condition2 value
## 12           chromatin            unknown     1
## 24             cytosol            unknown    25
## 29                  ER           lysosome     1
## 33                  ER                 PM     1
## 36                  ER            unknown     5
## 45               ERGIC                 PM     9
## 48               ERGIC            unknown     9
## 57                  GA                 PM     6
## 60                  GA            unknown    17
## 72            lysosome            unknown     1
## 84       mitochondrion            unknown    11
## 96             nucleus            unknown    16
## 111                 PM                 ER     2
## 120                 PM            unknown    25
## 132         proteasome            unknown     1
## 144 ribosome/complexes            unknown    15
## 145            unknown          chromatin    10
## 146            unknown            cytosol    84
## 147            unknown                 ER    18
## 148            unknown              ERGIC    49
## 149            unknown                 GA    36
## 150            unknown           lysosome     5
## 151            unknown      mitochondrion     8
## 152            unknown            nucleus    38
## 154            unknown                 PM    56
## 155            unknown         proteasome    40
## 156            unknown ribosome/complexes    25



Finally, we extract our list of differentially localised proteins along with their predicted localisation.

## Construct data.frame
df_cands <- data.frame(
    fData(msnset_cands[[1]])[, c("bandle.differential.localisation",
                                 "bandle.allocation.pred")],
    fData(msnset_cands[[2]])[, "bandle.allocation.pred"])

colnames(df_cands) <- c("differential.localisation",
                        "unstimulated", "xray")

## Order by highest differential localisation estimate
df_cands <- df_cands %>%
  arrange(desc(differential.localisation))

## View
df_cands %>%
  head()


##        differential.localisation       unstimulated               xray
## A2RUB1                         1            unknown ribosome/complexes
## O00258                         1            unknown            unknown
## O00423                         1            unknown            unknown
## O14578                         1 ribosome/complexes            unknown
## O14745                         1            unknown            unknown
## O15231                         1            unknown            unknown



## Discussion and conclusion

Subcellular proteomics is now commonly used to map protein location(s) within the cell. Mass spectrometry-based correlation profiling methods have been widely adopted due to their accessibility and simplicity, which is in part enabled by community-wide protocols and open-source informatics software. ^15^ Given the complexity of the data generated by an MS-based correlation profiling experiment, there is a clear need for comprehensive guidance regarding how to deal with such data and extract biologically meaningful conclusions.

Pre-existing workflows for the analysis of correlation profiling data have been presented as rigid protocols ^49, 9^ or focused on the application of a single tool, ^50^ thus providing limited flexibility for the user. We also previously published two workflows in this space: A Bioconductor workflow for processing and analysing spatial proteomics data ^11^ and A Bioconductor workflow for the Bayesian analysis of spatial proteomics. ^12^ The first of these utilised a protein-level dataset stored in an MSnSet to demonstrate the use of SVM classification for protein localisation, as well as novelty detection and transfer learning. ^11^ The second workflow was an extension of this, again starting with protein-level data in an MSnSet but guiding users through the application of Bayesian t-augmented Gaussian mixture models for protein localisation classification. ^12^ Importantly, both workflows remain relevant and useful for the analysis of correlation profiling subcellular proteomics data. However, as correlation profiling experiments become increasingly common and the question naturally extends from predicting primary localisation to predicting differential localisation, we recognised the need for a more in-depth workflow which covers data analysis from start to finish, provides a narrative to help users understand their options, and points out differences which may arise due to variations in experimental designs and methods.

The start-to-end workflow presented here covers the complete process by which users can (1) convert quantitative MS data matrices into into high quality correlation profiles, (2) assess and explore subcellular spatial resolution to inform optimal marker selection, (3) apply a range of supervised machine learning algorithms to predict protein localisation, and (4) utilise Bayesian ANalysis of Differential Localisation Experiments (BANDLE) ^10^ to predict protein differential localisation events. Our workflow goes further than existing correlation profiling workflows by not only providing instructions but openly discussing the dataset-specific decisions that must be made. This is particularly important with respect to the subcellular compartments included in the analyses and the marker proteins selected to represent these compartments. Moreover, we provide supplementary information and code to help users adapt this workflow to DDA data processed using the MaxQuant software as well as DIA data processed with DIA-NN. Overall, this workflow provides an in-depth and user-friendly guide to the analysis of static and dynamic MS-based correlation profiling experiments, which we hope will encourage the optimal use of these methods in the wider community.

## Session information

Below, we provide a summary of all packages and versions used to generate this document. It is possible that future software updates could lead to the generation of errors or results that differ to those presented in this workflow. Users with more recent package versions should be aware that the code may need to be altered slightly to avoid such errors.

sessionInfo()


## R version 4.5.0 (2025-04-11)
## Platform: x86_64-apple-darwin20
## Running under: macOS Sequoia 15.4.1
##
## Matrix products: default
## BLAS: /Library/Frameworks/R.framework/Versions/4.5-x86_64/Resources/lib/libRblas.0.dylib
## LAPACK: /Library/Frameworks/R.framework/Versions/4.5-x86_64/Resources/lib/libRlapack.dylib; LAPACK version 3.12.1
##
## locale:
## [1] en_US.UTF-8/en_US.UTF-8/en_US.UTF-8/C/en_US.UTF-8/en_US.UTF-8
##
## time zone: Europe/London
## tzcode source: internal
##
## attached base packages:
## [1] stats4   stats   graphics   grDevices utils   datasets   methods
## [8] base
##
## other attached packages:
## [1] pheatmap_1.0.12              ggpubr_0.6.0
## [3] gridExtra_2.3                pRolocdata_1.46.0
## [5] pRolocGUI_2.18.0             org.Hs.eg.db_3.21.0
## [7] clusterProfiler_4.16.0       dbscan_1.2.2
## [9] lubridate_1.9.4              forcats_1.0.0
## [11] stringr_1.5.1               dplyr_1.1.4
## [13] purrr_1.0.4                 readr_2.1.5
## [15] tidyr_1.3.1                 tibble_3.2.1
## [17] ggplot2_3.5.2               tidyverse_2.0.0
## [19] bandle_1.12.0               pRoloc_1.48.0
## [21] BiocParallel_1.42.0         MLInterfaces_1.88.1
## [23] cluster_2.1.8.1             annotate_1.86.0
## [25] XML_3.99-0.18               AnnotationDbi_1.70.0
## [27] MSnbase_2.34.0              ProtGenerics_1.40.0
## [29] mzR_2.42.0                  Rcpp_1.0.14
## [31] QFeatures_1.18.0            MultiAssayExperiment_1.34.0
## [33] SummarizedExperiment_1.38.1 Biobase_2.68.0
## [35] GenomicRanges_1.60.0        GenomeInfoDb_1.44.0
## [37] IRanges_2.42.0              S4Vectors_0.46.0
## [39] BiocGenerics_0.54.0         generics_0.1.4
## [41] MatrixGenerics_1.20.0       matrixStats_1.5.0
##
## loaded via a namespace (and not attached):
##   [1] R.methodsS3_1.8.2     shinydashboardPlus_2.0.5 progress_1.2.3
##   [4] vsn_3.76.0            nnet_7.3-20              DT_0.33
##   [7] Biostrings_2.76.0     vctrs_0.6.5              ggtangle_0.0.6
##  [10] digest_0.6.37         png_0.1-8                shape_1.4.6.1
##  [13] proxy_0.4-27          BiocBaseUtils_1.10.0     git2r_0.36.2
##  [16] ggrepel_0.9.6         parallelly_1.44.0        MASS_7.3-65
##  [19] reshape2_1.4.4        foreach_1.5.2            httpuv_1.6.16
##  [22] qvalue_2.40.0         withr_3.0.2              xfun_0.52
##  [25] ggfun_0.1.8           survival_3.8-3           MetaboCoreUtils_1.16.0
##  [28] memoise_2.0.1         hexbin_1.28.5            gson_0.1.0
##  [31] systemfonts_1.2.3     mixtools_2.0.0.1         ragg_1.4.0
##  [34] tidytree_0.4.6        GlobalOptions_0.1.2      gtools_3.9.5
##  [37] R.oo_1.27.1           Formula_1.2-5            prettyunits_1.2.0
##  [40] KEGGREST_1.48.0       promises_1.3.2           httr_1.4.7
##  [43] rstatix_0.7.2         globals_0.18.0           rstudioapi_0.17.1
##  [46] UCSC.utils_1.4.0      miniUI_0.1.2             DOSE_4.2.0
##  [49] ggalluvial_0.12.5     curl_6.2.2               ncdf4_1.24
##  [52] shinyhelper_0.3.2     randomForest_4.7-1.2     GenomeInfoDbData_1.2.14
##  [55] SparseArray_1.8.0     xtable_1.8-4             doParallel_1.0.17
##  [58] evaluate_1.0.3        S4Arrays_1.8.0           BiocFileCache_2.16.0
##  [61] preprocessCore_1.70.0 hms_1.1.3                bookdown_0.43
##  [64] colorspace_2.1-1      filelock_1.0.3           shinyWidgets_0.9.0
##  [67] magrittr_2.0.3        later_1.4.2              viridis_0.6.5
##  [70] ggtree_3.16.0         lattice_0.22-7           MsCoreUtils_1.20.0
##  [73] future.apply_1.11.3   cowplot_1.1.3            class_7.3-23
##  [76] pillar_1.10.2         nlme_3.1-168             iterators_1.0.14
##  [79] compiler_4.5.0        stringi_1.8.7            gower_1.0.2
##  [82] dendextend_1.19.0     plyr_1.8.9               crayon_1.5.3
##  [85] abind_1.4-8           gridGraphics_0.5-1       bit_4.6.0
##  [88] pcaMethods_2.0.0      fastmatch_1.1-6          textshaping_1.0.1
##  [91] codetools_0.2-20      recipes_1.3.0            bslib_0.9.0
##  [94] e1071_1.7-16          plotly_4.10.4            LaplacesDemon_16.1.6
##  [97] mime_0.13             splines_4.5.0            circlize_0.4.16
## [100] dbplyr_2.5.0          knitr_1.50               blob_1.2.4
## [103] clue_0.3-66           AnnotationFilter_1.32.0  fs_1.6.6
## [106] listenv_0.9.1         mzID_1.46.0              ggsignif_0.6.4
## [109] ggplotify_0.1.2       Matrix_1.7-3             statmod_1.5.0
## [112] svglite_2.2.1         tzdb_0.5.0               lpSolve_5.6.23
## [115] pkgconfig_2.0.3       tools_4.5.0              cachem_1.1.0
## [118] RSQLite_2.3.11        viridisLite_0.4.2        DBI_1.2.3
## [121] impute_1.82.0         fastmap_1.2.0            rmarkdown_2.29
## [124] scales_1.4.0          grid_4.5.0               usethis_3.1.0
## [127] shinydashboard_0.7.3  broom_1.0.8              sass_0.4.10
## [130] patchwork_1.3.0       coda_0.19-4.1            FNN_1.1.4.1
## [133] BiocManager_1.30.25   carData_3.0-5            lbfgs_1.2.1.2
## [136] rpart_4.1.24          farver_2.1.2             yaml_2.3.10
## [139] cli_3.6.5             lifecycle_1.0.4          caret_7.0-1
## [142] mvtnorm_1.3-3         backports_1.5.0          lava_1.8.1
## [145] kernlab_0.9-33        timechange_0.3.0         gtable_0.3.6
## [148] parallel_4.5.0        pROC_1.18.5              ape_5.8-1
## [151] limma_3.64.0          jsonlite_2.0.0           colourpicker_1.3.0
## [154] kableExtra_1.4.0      bit64_4.6.0-1            Rtsne_0.17
## [157] yulab.utils_0.2.0     gdata_3.0.1              jquerylib_0.1.4
## [160] GOSemSim_2.34.0       segmented_2.1-4          shinyjs_2.1.0
## [163] R.utils_2.13.0        timeDate_4041.110        lazyeval_0.2.2
## [166] shiny_1.10.0          BiocWorkflowTools_1.34.0 htmltools_0.5.8.1
## [169] affy_1.86.0           enrichplot_1.28.2        GO.db_3.21.0
## [172] rappdirs_0.3.3        glue_1.8.0               httr2_1.1.2
## [175] XVector_0.48.0        treeio_1.32.0            MALDIquant_1.22.3
## [178] mclust_6.1.1          igraph_2.1.4             R6_2.6.1
## [181] labeling_0.4.3        Spectra_1.18.0           aplot_0.2.5
## [184] ipred_0.9-15          DelayedArray_0.34.1      tidyselect_1.2.1
## [187] sampling_2.10         xml2_1.3.8               car_3.1-3
## [190] future_1.49.0         ModelMetrics_1.2.2.2     BiocStyle_2.36.0
## [193] affyio_1.78.0         data.table_1.17.2        htmlwidgets_1.6.4
## [196] fgsea_1.34.0          RColorBrewer_1.1-3       biomaRt_2.64.0
## [199] rlang_1.1.6           hardhat_1.4.1            prodlim_2025.04.28
## [202] PSMatch_1.12.0



## Data Availability

Raw mass spectrometry data is freely available online through ProteomeXchange via the PRIDE repository under the identifier PXD055123. The data required to run the main workflow and appendix are available from Zenodo under the identifier doi: 10.5281/zenodo.15100485. ^51^ Appendix also available from doi: 10.5281/zenodo.15100485 ^51^ and at the associated GitHub repository https://github.com/CambridgeCentreForProteomics/f1000_subcellular_proteomics .

## Software availability

This workflow was written in the R Statistical programming language and uses freely available open-source software packages from CRAN and Bioconductor. Version numbers for all packages are shown in the Session information section.

Source code available from: https://github.com/CambridgeCentreForProteomics/f1000_subcellular_proteomics . Archived software available from doi: 10.5281/zenodo.15100485. ^51^ Files and appendix are also available from Zenodo under the identifier doi: 10.5281/zenodo.15100485. License: CC-BY 4.0.
