# Supplementary material for: An updated Bioconductor workflow for correlation profiling subcellular proteomics
Source: F1000Res. 2025 Jul 21;14:714. [Version 1] doi: 10.12688/f1000research.165543.1 (PMC12419147; doi:10.12688/f1000research.165543.1)
Supplement: Peer review history

# Reviewer report: Reviewer response for version 1

**DOI:** 10.5256/f1000research.182186.r408868

**Published:** 2025-09-08

**Authors:** Peter Hains

**Affiliations:** 1 Children's Medical Research Institute, Westmead, NSW, Australia

The authors present a workflow for processing subcellular proteomic MS data. The title is somewhat misleading as this reviewer was expecting V2 (for example) of a prior release. This is not quite the case, so the title should be clarified. Overall the work is detailed, practical, adaptable, well written and advanced in nature. A relatively minor criticism is that the user needs to have a relatively high level of skill with R in order to take full advantage of the work described. Given this is the likely target audience this is to be expected.

 The paper is very long, this is both an asset and a liability. Given the length, some sort of index would be helpful for navigation purposes.

 It is good to see the authors have described how to adapt the TMT workflow to more generic DDA, or the increasingly important DIA, data types. The manuscript is replete with method discussion and processing notes that will aid the less familiar analyst.

 The authors go to considerable trouble to ensure readers are aware of the versions of the tools used for the analysis. This is essential given compatibility issues.

 It is the opinion of this reviewer the article should be indexed with minor changes. It will be a useful resource for the scientific community.

Are the conclusions about the tool and its performance adequately supported by the findings presented in the article?

Yes

Is the rationale for developing the new software tool clearly explained?

Yes

Is the description of the software tool technically sound?

Yes

Are sufficient details of the code, methods and analysis (if applicable) provided to allow replication of the software development and its use by others?

Yes

Is sufficient information provided to allow interpretation of the expected output datasets and any results generated using the tool?

Yes

Reviewer Expertise:

Proteomics, mass spectrometry, bioinformatics, data analysis, cancer

I confirm that I have read this submission and believe that I have an appropriate level of expertise to confirm that it is of an acceptable scientific standard.

# Reviewer report: Reviewer response for version 1

**DOI:** 10.5256/f1000research.182186.r405778

**Published:** 2025-09-05

**Authors:** Vanya Bhushan

**Affiliations:** 1 National Institutes of Health, Bethesda, Maryland, USA

The manuscript is well written and has been explained in detail. The authors have covered all bases, and that has improved the manuscript quality. All Bioconductor packages are well explained here, along with the corresponding R code. This will be very helpful in studying spatial-temporal protein dynamics in cells. I recommend indexing the article. These are my few comments to authors:

1. Please provide versions of all the Bioconductor packages (e.g, bandle, MSnbase, pROLOC). Also, please mention the compatible R version with all the packages. 
2. The authors have explained clearly how to make the input data as per the condition/s. 
3. Initial QC based on raw data in both LFQ and TMT-based experiments is well explained (sister workflow). 
4. FDR control at the protein level data is a key feature in determining the protein confidence. The authors have provided both relaxed and stringent options to work with. 
5. The authors need to give more explanation on label-free data filtering. They have explained TMT-labelled samples well, but what about the LFQ samples, where there is a high number of missing values? how to filter the data without losing relevant data from both unstim and treated samples?
6. In case of high missing values in, say, adjacent organelles like lysosomes and Golgi bodies, can they be merged? low protein quant in GB is a problem. Should it be merged with the nearest organelle or removed entirely in case of LFQ data?
7. In the imputation discussion paragraph, the authors have emphasized that their data looked better with KNN imputation. In the case of LFQ data, where missingness is high, will KNN be a fit? Also, 3 replicates per condition is too narrow a window to use KNN imputation. 
8. Concatenation gives equal weight to each replicate/gradient, but what if replicate quality or batch (different PD versions/gradients) differs?
9. How are imputed entries represented in the heatmap (e.g., masking/annotation)? Since imputation can create smoothness, did you compare heatmaps pre- and post-imputation or provide an imputed-value overlay?
10. For compartments having fewer than 10-15 markers, how should they be treated? Should it be merged with the adjacent compartment?
11. How do authors label or ‘leave unassigned’ clusters that don’t match any known compartment or compartments with fewer markers?

Are the conclusions about the tool and its performance adequately supported by the findings presented in the article?

Yes

Is the rationale for developing the new software tool clearly explained?

Yes

Is the description of the software tool technically sound?

Yes

Are sufficient details of the code, methods and analysis (if applicable) provided to allow replication of the software development and its use by others?

Yes

Is sufficient information provided to allow interpretation of the expected output datasets and any results generated using the tool?

Yes

Reviewer Expertise:

immunology, Mass spectrometry based proteomics, structural biology.

I confirm that I have read this submission and believe that I have an appropriate level of expertise to confirm that it is of an acceptable scientific standard.

# Reviewer report: Reviewer response for version 1

**DOI:** 10.5256/f1000research.182186.r403072

**Published:** 2025-09-04

**Authors:** Jeremy G Wideman, Yu-Ping Poh

**Affiliations:** 1 Arizona State University Biodesign Institute (Ringgold ID: 43363), Tempe, Arizona, USA

This manuscript provides a detailed Bioconductor workflow for correlated profiling subcellular proteomics. The authors summarize existing and newly developed tools, *QFeatures* and *BANDLE*, and present them in an accessible, step-by-step workflow covering installation, data quality assessment, missing data imputation, marker protein assignment, and both static and differential localization analyses. The workflow is particularly useful for multi-replicate, multi-condition experiments, making large-scale analyses more approachable in subcellular proteomics.

 We used the workflow from beginning to end on one of our datasets, and we found no obvious issues.

 We have a few comments regarding clarity and emphasis:

1. Redundancy with prior work
  The previous workflows have some redundancy with the current work (e.g., Hutchings et al., 2024). It would be helpful if the authors could clearly articulate the differences and improvements compared to the earlier protocol or alternatively reduce overlapping content.
2. Balance between code and empirical analyses
  While the inclusion of detailed code is valuable, at times the manuscript reads too much like a software manual. We encourage the authors to provide more comparisons from empirical analyses. For example, in the original workflow (Breckels et al., 2018), the analysis started from a protein-level database output, whereas this workflow begins with PSM-level data. Does starting from the PSM level improve quality control? How does it affect downstream results? Addressing such points would strengthen the manuscript.
3. Highlighting the novel contribution (BANDLE)
  Part 3, which introduces the use of *BANDLE*, appears to be the most novel and impactful section of this workflow. The figures illustrating protein translocation under different conditions are particularly compelling. We suggest that the authors emphasize this section more prominently, potentially by condensing some of the earlier, redundant material. This would make the manuscript easier to follow and draw attention to its main contribution.

Are the conclusions about the tool and its performance adequately supported by the findings presented in the article?

Yes

Is the rationale for developing the new software tool clearly explained?

Yes

Is the description of the software tool technically sound?

Yes

Are sufficient details of the code, methods and analysis (if applicable) provided to allow replication of the software development and its use by others?

Yes

Is sufficient information provided to allow interpretation of the expected output datasets and any results generated using the tool?

Yes

Reviewer Expertise:

Genomics, subcellular proteomics, evolution

We confirm that we have read this submission and believe that we have an appropriate level of expertise to confirm that it is of an acceptable scientific standard.
